# Nitric Oxide Antagonizes the Acid Tolerance Response that Protects *Salmonella* against Innate Gastric Defenses

**DOI:** 10.1371/journal.pone.0001833

**Published:** 2008-03-19

**Authors:** Travis J. Bourret, Steffen Porwollik, Michael McClelland, Rui Zhao, Todd Greco, Harry Ischiropoulos, Andrés Vázquez-Torres

**Affiliations:** 1 Department of Microbiology, University of Colorado Health Sciences Center, Aurora, Colorado, United States of America; 2 Sidney Kimmel Cancer Center, San Diego, California, United States of America; 3 Department of Biomolecular Structure, University of Colorado Health Sciences Center, Aurora, Colorado, United States of America; 4 Department of Pediatrics, Children's Hospital of Philadelphia and the University of Pennsylvania, Philadelphia, Pennsylvania, United States of America; 5 Department of Pharmacology, Children's Hospital of Philadelphia and the University of Pennsylvania, Philadelphia, Pennsylvania, United States of America; 6 Children's Hospital of Philadelphia and the University of Pennsylvania, Philadelphia, Pennsylvania, United States of America; Duke University Medical Center, United States of America

## Abstract

**Background:**

Reactive nitrogen species (RNS) derived from dietary and salivary inorganic nitrogen oxides foment innate host defenses associated with the acidity of the stomach. The mechanisms by which these reactive species exert antimicrobial activity in the gastric lumen are, however, poorly understood.

**Methodology/Principal Findings:**

The genetically tractable acid tolerance response (ATR) that enables enteropathogens to survive harsh acidity was screened for signaling pathways responsive to RNS. The nitric oxide (NO) donor spermine NONOate derepressed the Fur regulon that controls secondary lines of resistance against organic acids. Despite inducing a Fur-mediated adaptive response, acidified RNS largely repressed oral virulence as demonstrated by the fact that *Salmonella* bacteria exposed to NO donors during mildly acidic conditions were shed in low amounts in feces and exhibited ameliorated oral virulence. NO prevented *Salmonella* from mounting a *de novo* ATR, but was unable to suppress an already functional protective response, suggesting that RNS target regulatory cascades but not their effectors. Transcriptional and translational analyses revealed that the PhoPQ signaling cascade is a critical ATR target of NO in rapidly growing *Salmonella*. Inhibition of PhoPQ signaling appears to contribute to most of the NO-mediated abrogation of the ATR in log phase bacteria, because the augmented acid sensitivity of *phoQ-*deficient *Salmonella* was not further enhanced after RNS treatment.

**Conclusions/Significance:**

Since PhoPQ-regulated acid resistance is widespread in enteric pathogens, the RNS-mediated inhibition of the *Salmonella* ATR described herein may represent a common component of innate host defenses.

## Introduction

The acidity of the stomach is a primary line of host defense against food borne pathogens. Consequently, achlorhydria and hypochlorhydria associated with clinical syndromes as varied as pernicious anemia, gastric displasia or gastric carcinoma increase the incidence of gastrointestinal infections [1]–[5]. Accordingly, histamine-2 receptor antagonists and proton pump inhibitors that block gastric acidity predispose the host to suffer from an assortment of bacterial, fungal and parasitic infections [6]–[9]. In addition to exerting direct antimicrobial activity, low pH facilitates the nonenzymatic formation of RNS to enhance the antimicrobial barrier of the gastric juice [10]. RNS-dependent innate host defenses have been best characterized in professional phagocytes, in which the inducible NO synthase catalyzes the oxidation of L-arginine to L-citrulline for the generation of copious amounts of NO. In contrast, gastric RNS are primarily derived nonenzymatically from NO_3_^−^ actively concentrated in the salivary glands from the enterosalivary circulation [10]–[12]. Oral commensals on the posterior surface of the tongue reduce NO_3_^−^ to NO_2_^−^ [13], which upon acidification in the gastric lumen is protonated to nitrous acid (HNO_2_). This species is a precursor to a variety of RNS such as NO, nitrogen dioxide (NO_2_^•^) and dinitrogen trioxide (N_2_O_3_). RNS produced at the low pH normally found in the stomach exert potent antimicrobial activity towards several enteropathogens, including the dimorphic fungus *Candida albicans,* and the enteric bacteria *Eschericia coli, Salmonella enterica, Shigella sonnei* and *Yersinia enterocolitica* [10], [14]–[16]. The mechanisms by which RNS mediate broad host defense in the gastric lumen remain, however, largely unknown.

Bacteria that establish close interactions with mammalian hosts experience nutritional limitations, temperature shifts, osmolarity and pH fluctuations, as well as oxidative and nitrosative stresses. Although for most bacteria the harsh acidity of the stomach is a formidable barrier to infection, several pathogenic microorganisms acquired via fecal-oral transmission show innate resistance to the acidity of the gastric lumen. For example, *Shigella* spp. and certain strains of *E. coli* are strong acidophiles remarkable for their innate resistance to low pH [17], [18]. In addition, most members of the enterobacteriaceae family are capable of mounting a genetic program known as the acid tolerance response (ATR) that enhances resistance to extreme acidity. The ATR may be stimulated in the environment upon contact of the pathogen with acidic foods or triggered *in situ* in the gastric lumen in response to rises in pH that accompany the consumption of a meal [18], [19]. The ATR is associated with the expression of more than 50 acid shock proteins [20], [21] controlled by a variety of signaling pathways. For instance, in actively growing *Salmonella*, the alternative sigma factor S (σ^s^) and the ferric uptake regulator (Fur) coordinate the ATR that provides resistance to organic acids, whereas the PhoPQ two component regulatory system directs resistance to inorganic acid stress [22]–[24]. Independently, the response regulator OmpR coordinates the ATR to inorganic acid stress in stationary phase bacteria [25]. Because RNS have been linked to the innate defenses of the stomach, we setout to investigate whether acidified RNS can alter the ability of *Salmonella* to mount an ATR. The studies presented herein have revealed that RNS induce acid sensitivity in rapidly growing *Salmonella* by inhibiting the PhoPQ-dependent ATR.

## Methods

### Bacterial Strains

*Salmonella enterica* serovar Typhimurium strain 14028s ATCC was used throughout this study as wild-type and as a background for the construction of mutant alleles (table 1). Mutations were generated using the method described previously by Datsenko and Wanner [26]. PCR amplification products encompassing the Flp recognition target (FRT)-flanked kanamycin resistance cassette of the pKD13 plasmid were generated using True Fidelity DNA Polymerase (CLP Inc., San Diego, CA) and primers encoding 60 nucleotides of target genes (table S4). The resulting PCR products were *DpnI* digested and electroporated into *S*. Typhimurium strain TT22236 carrying the pTP2223 plasmid expressing the λ red recombinase under Ptac control. Mutations were moved into *S.* Typhimurium strain 14028s by P22-mediated transduction and pseudolysogens eliminated by streaking on Evans blue uranine agar plates. In-frame deletions were generated by recombining the two FRT sites flanking the kanamycin resistance cassette with the Flp recombinase encoded by the temperature sensitive pCP20 plasmid. The mutations were confirmed by PCR analysis. Transcriptional *lacZY* fusions were constructed by the pCP20-mediated integration of the pCE36 plasmid encoding a promoterless *lacZY* gene into unique FRT scars of selected genes [27]. *Salmonella* strain AV0475 carrying a Δ*phoQ::FRT* mutant allele was complemented with the low-copy vector pWSK29 expressing a wild-type *phoQ* allele amplified from the Δ*phoP::FRT Salmonella* strain AV0474. C-terminal 3×FLAG-tagged fusions to *phoP* and *phoQ* were constructed as described previously [28] using the template plasmid pSUB11 and primer sets listed in table S4.

**Table 1:** Bacterial Strains and Plasmids

Strains	Description	Reference
*Salmonella* Typhimurium strain 14028s	Wild-type	ATCC
AV0474	Δ*phoP::FRT*	This study
AV0475	Δ*phoQ::FRT*	This study
AV0560	Δ*phoQ::FRT pWSK29*::*phoQ*	This study
AV0611	Δ*lpxO::lacZ*	This study
AV0614	Δ*phoQ::FRT* Δ*lpxO::lacZ*	This study
AV0322	*ompR::Tn10* in 14028s	This study from [68]
AV0473	Δ*fur::FRT*	This study
AV06108	Δ*rpoS::km*	This study
AV06115	*phoP::3xFLAG-FRT*	This study
AV07131	*phoQ::3xFLAG-FRT*	This study
AV0202	*spiC::lacZ*	[41]
IR715	Wild-type nalidixic acid^r^	[69]
**Plasmids**		
pCP20	*bla cat cI*857 λP_R_ *flp* pSC101 oriTS	[70]
pKD13	*bla* FRT *ahp* FRT PS1 PS4 oriR6K	[26]
pWSK29	*bla lacZ* oripSC101	[71]
pSUB11	*3xFLAG* FRT *ahp* FRT *bla* R6K*ori*V	[28]
pCE36	*ahp* FRT *lacZY^+^* t*_his_* oriR6K	[27]

### Acid Tolerance Assays

Acid tolerance assays were performed as previously described [24] with slight modifications. Briefly, overnight *Salmonella* cultures grown in Luria Bertani (LB) broth at 37°C with shaking were subcultured 1:50 in minimal EG medium (0.2 g/L MgSO_4_, 2 g/L C_6_H_8_O_7_-H_2_O, 10 g/L K_2_HPO_4_, 3.5 g/L Na(NH_4_)HPO_4_-4H_2_O, and 4 g/L D-glucose) [29], pH 7.0. The cultures were grown to an OD_600nm _of 0.4 (∼2×10^8^ CFU/ml). Selected groups of *Salmonella* tagged for adaptation were cultured in fresh EG medium, pH 4.4 for 2 h at 37°C with shaking. EG medium was used in our assays because it has been widely employed to study the ATR of *Salmonella* [24], [30]. Unless specified, the NO donor spermine NONOate (Cayman Chemical, Ann Arbor, MI) or NaNO_2_^−^ were added at the beginning of the adaptation period. Adapted cultures were then pelleted by centrifugation to remove spent EG medium along with spermine NONOate and NaNO_2_^−^. Adapted and nonadapted cultures were acid challenged in fresh EG medium, pH 3.0. The number of *Salmonella* surviving at various timepoints after acid challenge were enumerated on LB agar plates. Percent survival was calculated as (CFU t_n_/CFU t_0_)×100.

### Mouse Infections

Six- to 8-week old C57BL/6 mice bred in our animal facility according to Institutional Animal Care and Use Committee guidelines were used to assess the effect of acidified spermine NONOate on *Salmonella* oral virulence. Briefly, mice were inoculated with ∼5×10^5^ CFU/mouse of the nalidixic acid resistant *S.* Typhimurium strain IR715 (table 1) grown to mid-log phase in EG medium, pH 7.0 (nonadapted) or with controls cultured for one additional hour in EG medium, pH 4.4 in the presence or absence of 250 µM spermine NONOate (adapted vs. adapted+NO). The mice were not starved before oral challenge. The dosis employed for the oral challenge studies, which is close to the oral LD_50_ of wild-type *S*. Typhimurium, has been used by multiple investigators [31]–[33]. Survival of mice was monitored over time and bacterial shedding was determined by plating fecal samples on LB agar plates supplemented with 50 µg/ml nalidixic acid.

### RNA Isolation

RNA was isolated from nonadapted *Salmonella* cultures grown to an OD_600nm_ of 0.4 in 20 ml EG medium, pH 7.0 or from controls adapted for 1 h in EG medium, pH 4.4 in the presence or absence of 250 µM spermine NONOate. Samples for transcriptional analysis were collected after one hour of adaptation because this time period was required for the expression of a protective ATR. *Salmonella* cultures were mixed with a 5 ml phenol (5%)/ethanol (95%) solution and placed on ice for 20 min. RNA was extracted from bacteria using the SV Total RNA Isolation kit. Complementary cDNA was synthesized from 1 µg of total RNA at 42°C for 30 min using MMLV reverse transcriptase, Rnasin, 4mM dNTPS, and 1.2 µg/ml random hexanucleotides (all reagents from Promega). Transcription of selected PhoPQ-regulated genes, *hmpA* and *rpoD* was determined by standard PCR using the synthesized cDNAs and the primers listed in table S4.

### Microarray Analysis

Microarray analysis was performed using a *Salmonella* whole ORF PCR product microarray [34]. Fluorescently labeled cDNAs were generated using Superscript-II reverse transcriptase in a reaction containing 1.2 µg/ml of random hexanucleotides, 4 µl Cy3- or Cy5-labelled dUTP (Amersham, Piscataway, NJ) and biased nucleotides (25mM dCTP, 25mM dATP, 25mM dGTP, and 10mM dTTP) and 50 µg of total RNA. Contaminating RNA was removed by hot alkali treatment and cDNAs purified using the Qiagen PCR Purification kit (Qiagen, Valencia, CA). Equal amounts of oppositely labeled cDNAs were hybridized to the *Salmonella* array. Microarrays scanned on a Genepix 4000A® microarray scanner were analyzed using the Genepix software (Molecular Devices, Sunnyvale, CA). Cy3 and Cy5 median signal intensities were derived by subtracting background intensity from spot-boundary signal intensities. Differential gene expression was calculated from three independent experiments and statistical significance determined utilizing the Significance Analysis for Microarrays (SAM) software package (Stanford University, http://www-stat.stanford.edu/tibs/SAM) [35].

### β-galactosidase assays

Isogenic strains harboring *lacZY* transcriptional fusions grown to OD_600nm_ of 0.4 in EG medium, pH 7.0 were adapted for 2 h in EG medium, pH 4.4 in the presence or absence of 250 µM spermine NONOate. The expression of the *lacZY* transcriptional fusions were quantified spectrophotometrically as β-galactosidase enzymatic activity using the substrate o-nitrophenyl-β-D-galactopyranoside. β-galactosidase activity is expressed in Miller units using the equation: 1,000×[(OD_420nm_−1.75×OD_550nm_)/(T_(min)_ ×V_(ml)_×OD_600nm_)] [36].

#### Oxygen consumption

Log-phase *Salmonella* were grown under acid tolerance assay conditions in EG medium, pH 4.4 in the presence or absence of 250 µM spermine NONOate. Consumption of oxygen was recorded with an oxygen probe using a free radical analyzer (WPI Inc., Sarasota, FL).

### Western blots

Bacterial strains harboring 3×FLAG fusions were grown under the acid tolerance assay conditions described above. Two milliliters of bacterial cultures grown to OD_600_ of 0.4 (2×10^8^ CFU/ml) were pelleted by centrifugation and resuspended in 500 µL of alkaline lysis buffer (25 mM Tris, 100 mM SDS, and 128 mM NaOH). The protein concentration in the crude lysates was estimated using the BCA Protein Assay (Pierce, Rockford, IL) and the samples were normalized to a concentration of 100 µg/ml. The specimens were subjected to 10% SDS-PAGE, transferred to nitrocellulose membranes, and probed with the anti-FLAG M2 monoclonal antibody (Sigma-Aldrich) followed by a horseradish peroxidase-conjugated anti-mouse IgG secondary antibody. Detection was carried out using the Enhanced Chemiluminescence Kit (GE Healthcare, Piscataway, NJ) on a Molecular Imager Fx (BioRad, Hercules, CA).

#### Statistical analysis

Data are expressed as mean±SEM. The data were analyzed using a paired Student's *t* test. To determine statistical significance between multiple comparisons, one-way analysis of variance (ANOVAs) were performed, followed by a Bonferroni posttest. Data were considered statistically significant when *p* was <0.05.

## Results

### RNS sensitize *Salmonella* to acid stress

**Figure 1:**
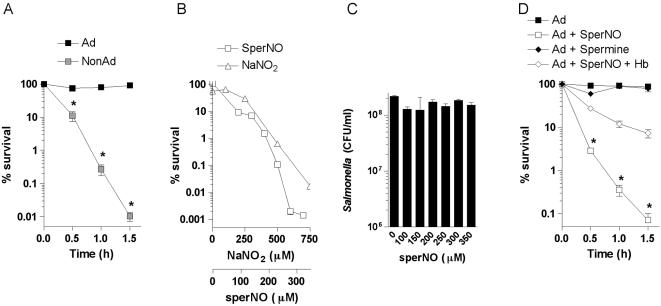
Sensitivity of *Salmonella* to acid stress upon exposure to NO donors. (A) *Salmonella* grown to an OD_600_ of 0.4 in EG medium, pH 7.0 were acid challenged in fresh EG medium, pH 3.0 (NonAd). Selected groups of mid-log phase cells were adapted (Ad) for 2 h in EG medium, pH 4.4 at 37°C. The % of surviving bacteria was estimated over time. (B) Effects of RNS on the ability of *Salmonella* to mount an ATR were determined by adding NO donors spermine NONOate (sperNO) and NaNO_2_ to bacteria during the 2 h adaptation period. The NO donors were removed by pelleting the bacteria before 1 h of challenge in EG medium, pH 3.0. (C) The effects that NO generated from sperNO exert on the viability of *Salmonella* grown for 2 h in EG medium, pH 4.4 can be seen in panel C. The effect that 10 µM of the NO-scavenger hemoglobin (Hb) had on the sperNO-inhibitable ATR is shown in panel D. The spermine base was used as a negative control. The chemicals were added for 2 h during the adaptation in EG medium, pH 4.4. The data represent the mean±SEM of 4-16 independent observations from 2–4 separate experiments. *, *p*<0.001 compared to adapted controls.

As predicted [20], [21], *Salmonella* adapted for 2 h in EG medium, pH 4.4 exhibited increased survival after 1.5 h of acid challenge in fresh EG medium, pH 3.0 (fig. 1A). In agreement with published investigations [20], [21], [24], [30], [37], between 55 and 90% of the bacteria adapted at pH 4.4 survived acid challenge at pH 3.0. Salivary NO_2_^−^ and NO_3_^−^ concentrations ranging from 400 to 1,890 µM generate several RNS with potent antimicrobial activity in the gastric juice [10]. Because the mechanisms for the broad antimicrobial activity exhibited by acidified RNS are poorly understood, we examined the effect that NO donors have on the ability of actively growing *Salmonella* to mount a productive ATR. Both spermine NONOate and NaNO_2_ abrogated in a dose dependent manner the acid resistance of adapted *Salmonella* (fig. 1B). Addition of 250 µM spermine NONOate or 500 µM NO_2_^−^ during the adaptation period resulted in a 100-fold decrease in *Salmonella* survival upon challenge in EG medium, pH 3.0 (fig. 1B). Henceforth, 500 µM NaNO_2_ or 250 µM spermine NONOate were used throughout the remainder of our investigations. These RNS were not directly bactericidal at pH 4.4 because the viability of *Salmonella* was unaffected even after treatment with 350 µM spermine NONOate (fig. 1C). Similar to pH 4.4, spermine NONOate induced bacteriostasis (not shown). The acid sensitivity resulting from spermine NONOate treatment was dependent on the release of NO, as indicated by the fact that the parent compound spermine lacked inhibitory activity (fig 1D). Furthermore, the NO scavenger ferrous hemoglobin significantly (*p*<0.001) antagonized the inhibitory effects of spermine NONOate. These results indicate that NO and NO_2_^−^ are not directly bactericidal under moderately acidic conditions normally encountered in the stomach following the consumption of a meal [19], but instead suggest that nitrogen oxides suppress the adaptive ATR that protects *Salmonella* from the rigors of the extreme acidity normally found in the stomach.

### RNS abrogate the *Salmonella* ATR

**Figure 2:**
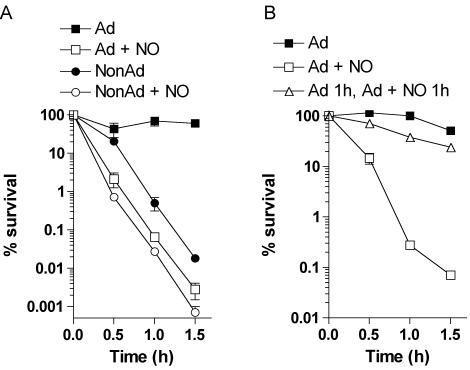
RNS inhibit the *Salmonella* ATR. The susceptibility of adapted (Ad) and nonadapted (NonAd) *Salmonella* cultures to acid challenge in EG medium, pH 3.0 was determined as described in figure 1. (A) Selected groups of Ad and NonAd bacterial cultures were treated with 250 µM spermine NONOate (NO) for 2 h before acid challenge. (B) The acid sensitivity of Ad and Ad+NO *Salmonella* was compared to controls grown for 1 h in EG medium, pH 4.4 before exposure to 250 µM spermine NONOate for 1 h (Ad 1 h, Ad+NO 1 h). The data represent the mean±SEM of 4–8 independent observations from 2–3 separate experiments.

To ascertain whether the acid sensitivity induced by RNS is dependent on the inhibition of the ATR, nonadapted and adapted *Salmonella* were treated for 2 h with 250 µM spermine NONOate before challenge in EG medium, pH 3.0. Both groups of bacteria exhibited similar acid sensitivity upon challenge at pH 3.0 (fig. 2A). Interestingly, nonadapted cells survived in EG medium, pH 3.0 significantly better than nonadapted controls treated with spermine NONOate (fig. 2A), an observation that may reflect the ability of bacteria to establish a weak ATR when directly acid-challenged in EG medium, pH 3.0. In contrast, *Salmonella* preadapted in EG medium, pH 4.4 for 1 h prior to exposure to spermine NONOate remained acid resistant (fig 2B). The fact that NO was unable to suppress an already functional protective response strongly argues in favor of a model in which RNS target ATR regulatory cascades but not their effectors.

### Inhibition of the ATR by RNS reduces *Salmonella* oral virulence

**Figure 3:**
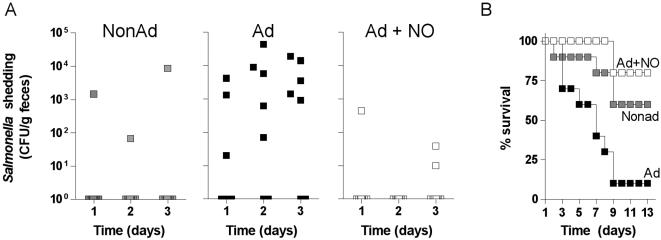
RNS suppress the increased oral virulence of ATR-adapted *Salmonella*. C57BL/6 mice were inoculated orally with ∼5×10^5^ CFU of NonAd, Ad and Ad+NO *Salmonella* grown as described in figure 1. (A) Fecal shedding of nalidixic acid resistant *Salmonella* was monitored in individual mice for 3 days after oral inoculation. Panel B shows the % of mice that survived after oral challenge with *Salmonella*. Data represent 10 mice per group from 2 separate experiments.

To determine whether the acid sensitivity seen in *Salmonella* treated with spermine NONOate affects passage through the gastrointestinal tract, C57BL/6 mice were infected per orally with ∼5×10^5^ CFU of the nalidixic acid resistant *Salmonella* strain IR175 grown under the acid tolerance assay conditions described above. This inoculum, which has been used by multiple investigators, is close to the oral LD_50_ for wild-type *S*. Typhimurium [31]–[33]. Shedding of *Salmonella* in the feces shortly after oral challenge was used as a marker for successful passage through the gastrointestinal tract. A higher percentage of mice challenged with *Salmonella* adapted in EG medium, pH 4.4 shed bacteria in feces than controls challenged with nonadapted bacteria (one-way ANOVA, *p*<0.01) (fig. 3A). To the best of our knowledge, these data demonstrate for the first time that bacteria that have mounted an ATR exhibit higher oral virulence. Treatment of *Salmonella* with 250 µM spermine NONOate during culture in EG medium, pH 4.4 significantly (one-way ANOVA, *p*<0.01) abrogated the increased shedding seen in adapted controls (fig. 3A). Fecal shedding of *Salmonella* reflected oral virulence. About 10% of mice infected with acid adapted *Salmonella* survived after 9 days of infection, whereas survival rates reached 60% to 80% in mice infected with either nonadapted or spermine NONOate-treated, adapted controls (fig. 3B).

### Effect of RNS on the transcriptional responses of *Salmonella* cultured in pH 4.4

**Figure 4:**
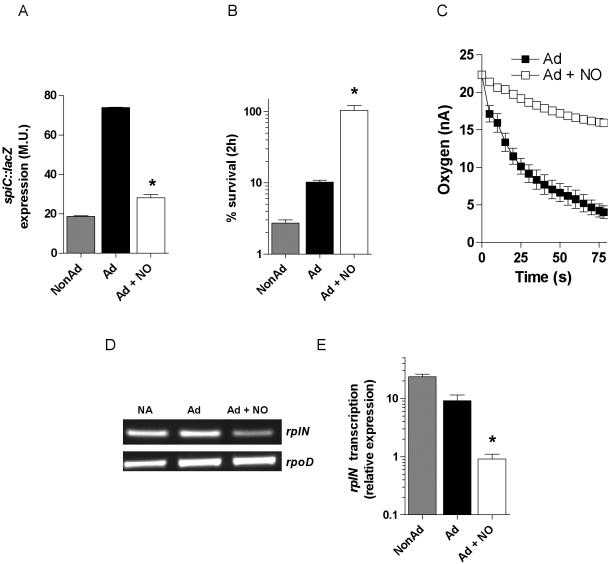
Responses of *Salmonella* to acidified RNS. The acid-inducible expression of the SPI2-encoded *spiC::lacZ* transcriptional fusion was compared in nonadapted (NonAd), adapted (Ad) and adapted+250 µM spermine NONOate (Ad+NO) *Salmonella* cultures (A). The survival of NonAd, Ad and Ad+NO *Salmonella* cultures exposed for 1 h to 400 µM H_2_O_2_ in PBS is shown in panel B. Oxygen consumption was monitored in Ad and Ad+NO *Salmonella* cultures (C). The transcription of the ribosomal protein-encoding gene *rplN* relative to the housekeeping gene *rpoD* was monitored by semi-quantitative (D) and quantitative RT-PCR (E). Relative expression is represented as the ratio of rplN/rpoD transcripts. Data represent the mean±SEM of 3–6 independent observations. *, p<0.05 by one-way ANOVA.

Microarray analysis was used to identify loci differentially transcribed in response to acidified RNS. The SAM software package [35] that ascribes statistical significance based upon the false discovery rate and q-values revealed that 1,760 of 4,350 coding sequences are differentially transcribed in response to 250 µM spermine NONOate in *Salmonella* grown for 1 h in EG medium, pH 4.4. When the analyses were restricted to loci modified ≥2-fold, 644 and 575 genes were found to be induced and repressed, respectively, upon spermine NONOate treatment (table S1). NO-induced genes belonged to metabolic pathways involved in iron and nitrogen metabolism, the SOS response, motility, adherence and invasion, whereas repressed groups included genes of the *Salmonella* pathogenecity island-2 (SPI-2), resistance to oxidative stress, the electron transport chain (ETC), transcriptional and translational machinery, cysteine biosynthesis, and the PhoP regulon (table 2). Iron acquisition genes encoded by the *sitABCD* operon and the *iroA* locus were differentially induced in response to spermine NONOate (table S2), likely reflecting their derepression upon nitrosylation of the iron prosthetic group of Fur [38]–[40]. Acidified spermine NONOate also induced the SOS regulator *lexA*, the universal stress protein *uspA* and several loci involved in DNA repair, while upregulating transcription of *hmpA* (flavohemoglobin), *narJ* (nitrate reductase) and *nrfA* and *nrfC* (nitrite reductases) involved in NO detoxification and nitrogen metabolism (table S2). Additionally, NO stimulated the expression of invasion genes, including the transcriptional activator *hilA* and the *invA, invB, invC, sopB, sopE2* and *sicA* structural and effector components of SPI-1 (table S2). As predicted [41], 25 genes from the SPI-2 regulon representing loci encoded inside and outside the pathogenicity island were found to be repressed by acidified spermine NONOate (table S3). Transcriptional analysis of *Salmonella* strain AV0212 encoding *spiC::lacZ* confirmed the repression of SPI-2 expression by acidified RNS. (fig. 4A). The stomach engenders oxidative stress upon *Salmonella* infection [42]. It is therefore not surprising that the *Salmonella* ATR confers cross-protection from oxidative stress [43]. Unexpectedly, acidified spermine NONOate repressed the transcription of antioxidant genes encoding glutaredoxin (*grxB* and *grxC*), superoxide dismutase A (*sodA*), catalase E (*katE*) and homocysteine biosynthesis (*metC* and *metL*). Despite their inhibition, spermine NONOate-treated *Salmonella* were highly resistant to H_2_O_2_ (fig. 4B), likely reflecting transient NO-mediated respiratory arrest [44]. In addition to inhibiting the enzymatic activity of terminal cytochrome oxidases [44], acidified spermine NONOate repressed transcription of the *nuo, sdh* and *cyo* operons encoding for the complex I NADH dehydrogenase, the complex II succinate dehydrogenase and the complex III cytochrome oxidase *bo* of the ETC. Together with the NO-mediated nitrosylation of terminal cytochromes [44], transcriptional inhibition of the ETC may contribute to the respiratory arrest associated with exposure to NO (fig. 4C). Moreover, spermine NONOate also inhibited the expression of the *atp* operon encoding the terminal F_0_F_1_ ATPase (table S3). In turn, acidified spermine NONOate appears to induce the stringent response, as indicated by the fact that the three major ribosomal protein operons (S10, α, and spv), multiple tRNA synthetases, protein elongation factors and RNA polymerase subunits *rpoB* and *rpoC* were downregulated (table S3). The NO-mediated transcriptional repression of the translational machinery was confirmed by semi-quantitative and quantitative RT-PCR analyses of the ribosomal protein encoded by *rplN* (fig. 4D & E).

**Table 2:** Classification of NO-responsive genes in Salmonella cultured in EG medium pH 4.4

Functional Gene Group	No. of Genes
***Induced***	
Iron acquisition	10
SOS response	9
Nitrogen Metabolism	6
Invasion, Adherence, and Motility	
SPI1	9
Flagella	8
Fimbriae	23
***Repressed***	
OmpR regulon	3
SPI2 regulon	25
Transcriptional-Translational Machinery	
Ribosomal Proteins	39
tRNA synthetases	13
RNA polymerase	3
Other	15
Cysteine Biosynthesis	12
Oxidative Stress	7
Respiratory Chain	25

### RNS suppress PhoPQ-dependent signaling

**Figure 5:**
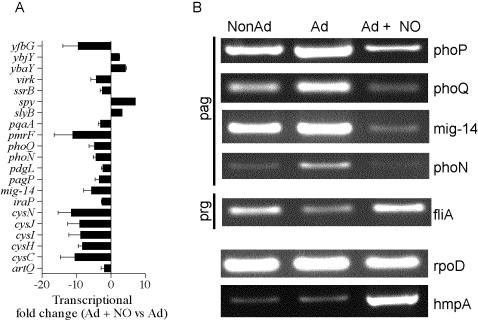
Acid-inducible PhoPQ-dependent gene transcription is repressed by RNS. Panel A shows the expression of PhoP-activated genes in rapidly growing *Salmonella* cultured for 1 h in EG medium, pH 4.4 in the presence (Ad+NO) or absence (Ad) of 250 µM spermine NONOate. Microarray data are represented as the mean fold change±SD from 3 independent experiments. (B) Transcription of PhoP-activated genes (pag), PhoP-repressed genes (prg), the control housekeeping *rpoD* gene and the NO-inducible *hmpA* gene were assessed by RT-PCR of RNA isolated from NonAd, Ad, and Ad+NO bacterial cultures grown as described in figure 1.

The essential role that the PhoPQ two component regulatory system plays in controlling the ATR is demonstrated by the fact that 1) PhoP is an acid shock protein and 2) strains harboring *phoP* or *phoQ* mutations are exquisitely acid sensitive [24], [45]. Because PhoPQ signaling has been shown to dominate the log phase ATR studied herein [24], [46], we examined in more detail the effect that acidified spermine NONOate has on PhoP-dependent gene transcription. Most members of the PhoP regulon, such as *phoQ*, *phoN*, *virK*, *pagP*, *pqaA*, *cysJIH* and *cysCND* [47], [48], were repressed by NO in *Salmonella* grown in EG medium, pH 4.4 (fig. 5A). The few PhoP-activated genes that were not repressed by NO may be a consequence of a differential regulation of these loci in EG medium, pH 4.4. RT-PCR analysis independently showed an NO-dependent downregulation in the transcription of PhoP-activated genes *phoP*, *phoQ*, *mig-14* and *phoN,* while RNS treatment did not affect (e.g., *rpoD*) or even increased (e.g., *hmpA*) the expression of other loci (fig. 5B). According to the idea that NO represses PhoPQ signaling, transcription of the PhoP-repressed genes (prg) *fliA* (fig. 5B), *fliC* and *hilA* was upregulated in response to spermine NONOate treatment (table S2). The low levels at which prg are normally expressed [49] may have contributed to the fact that our array analysis did not show a broader derepression of the PhoP regulon.

**Figure 6:**
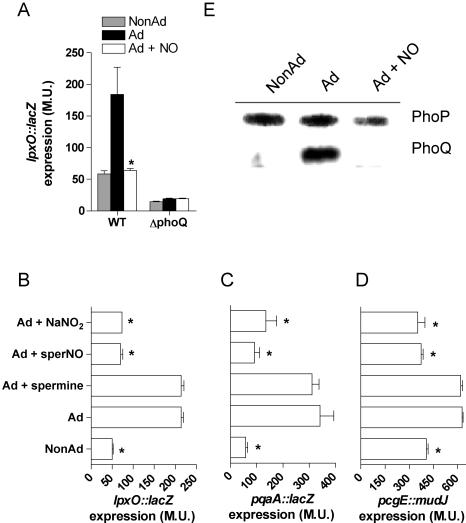
Suppression of PhoPQ-dependent gene transcription is mediated by nitrogen oxides and requires an intact signal transduction cascade. The effect of spermine NONOate on the PhoPQ-dependent induction of the *lpxO::lacZ* transcriptional fusion is shown in panel A. β∼-galactosidase activity (Miller Units, M.U.) is represented as the mean±SEM of 4–6 independent observations from 2–3 separate experiments. *, *p*<0.002 compared to adapted controls. The acid-inducible expression of the PhoP-activated loci *lpxO*, *pqaA* and *pcgE* were monitored in the presence or absence of 250 µM spermine, 250 µM spermine NONOate or 500 µM NaNO_2_ (B–D). (E) The expression of C-terminal 3×FLAG-tagged PhoP and PhoQ proteins was monitored in NonAd, Ad, and Ad+NO *Salmonella* cultures grown as described in figure 1.

The effect that NO has on PhoP-mediated transcription were independently studied using *lacZ* transcriptional fusions. Consistent with the DNA arrays and RT-PCR analysis, NO repressed the expression of *lpxO::lacZ*, *pqaA::lacZ* and *pcgE::mudJ* (fig. 6A–D). In the absence of the sensor kinase PhoQ, *lpxO::lacZ* was not only unresponsive to a drop in pH but its basal levels of expression were unaffected by spermine NONOate (fig. 6A). The suppressive effects of spermine NONOate on PhoP-dependent gene transcription appear to be directly related to the production of RNS because the polyamine base spermine did not suppress the acid-induced expression of the PhoP-activated loci *lpxO*, *pqaA*, and *pcgE* (fig. 6B–D). Furthermore, these loci were also repressed upon exposure of *Salmonella* to 500 µM NaNO_2 _in EG medium, pH 4.4 (fig. 6B–D). Because the expression of the PhoP regulon depends on enzymatic activity and abundance of the PhoQ sensor kinase and the PhoP response regulator, protein levels of the components of this two-component regulatory system were monitored in western blots of *Salmonella* strains harboring PhoP or PhoQ C-terminal 3×FLAG epitope tags. The amount of PhoP and PhoQ increased by 1.3- and 4.9-fold after adaptation of *Salmonella* for 2 h in EG medium, pH 4.4 (fig. 6E). Consistent with the transcriptional profiles, acidified spermine NONOate reduced *Salmonella* PhoP and PhoQ protein levels by 2- and 9.4-fold, respectively. Collectively, these data demonstrate that nitrogen oxides repress the acid-inducible, PhoPQ signaling cascade.

### RNS reversed the survival advantage associated with the PhoPQ-dependent ATR

**Figure 7:**
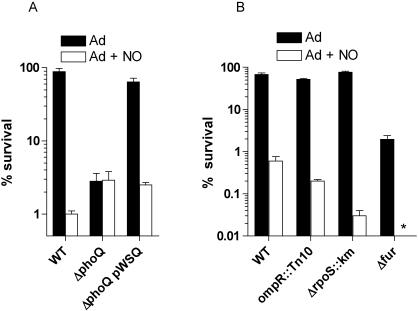
Acidified RNS suppress the PhoPQ-dependent ATR. Killing of wild-type *Salmonella* and its isogenic *ΔphoQ::FRT* control in EG medium, pH 3.0 is shown in panel A. The *ΔphoQ::FRT* mutation was complemented with a wild-type allele expressed from pWSQ. Selected groups of bacteria were treated with 250 µM spermine NONOate (Ad+NO) during the adaptation in EG medium, pH 4.4. Panel B compares the acid sensitivity of Ad and Ad+NO groups of *Salmonella* strains isogenic for *ΔrpoS::km*, *ompR::Tn10* and *Δfur::FRT*. The data represent the mean±SEM of 6–9 independent observations from 2–3 separate experiments. *, *p*<0.05 compared to adapted controls. **, no viable cells were detected after the adapted+NO group was challenged for 1 h in EG medium, pH 3.0.

The PhoPQ two component regulatory system dominates the adaptive response of rapidly growing *Salmonella* exposed to inorganic acid stress. We therefore tested whether the NO-mediated inhibition of the PhoP regulon is responsible for the RNS-dependent repression of the ATR seen herein using log phase *Salmonella*. Acid-adapted, *ΔphoQ* mutant *Salmonella* was as acid sensitive as wild-type bacteria grown in EG medium, pH 4.4 in the presence of 250 µM spermine NONOate (fig. 7A). Remarkably, the intrinsic hypersusceptibility of the *ΔphoQ* strain to acid stress was not increased further after spermine NONOate treatment. The phenotype exhibited by the *ΔphoQ* mutant appears to be specific, because the low copy plasmid pWSQ harboring a wild-type *phoQ* allele under the control of its native *phoPQ* promoter not only restored the ability of rapidly growing *Salmonella* to mount an ATR but also reestablished its sensitivity to spermine NONOate (fig. 7A). In contrast to the *ΔphoQ* defective allele, mutations in the ATR regulators *rpoS* and *ompR* had little effect on the ability of actively growing *Salmonella* to mount an ATR (fig. 7B). The ATR mounted by an *ompR*-deficient strain was as susceptible to RNS-mediated inhibition as that of wild-type controls. However, spermine NONOate-treated, acid adapted *ΔrpoS* mutant *Salmonella* were 10-fold more susceptible to acid stress than wild-type controls. Similarly, the partial protective response seen in a *Salmonella* strain bearing a defective *fur* allele was completely eliminated after spermine NONOate treatment. Since *rpoS* and *fur* coordinate the ATR of rapidly growing bacteria in response to organic acids [22], [23], these data suggest that spermine NONOate-treated log phase *Salmonella* set up a secondary line of defense in response to organic acids arising from fermentative pathways.

## Discussion

The host defenses associated with the extreme gastric acidity are potentiated through the nonenzymatic generation of RNS [50]. The studies presented herein were designed to shed light into the mechanisms by which acidified RNS contribute to innate immunity against the enteropathogen *Salmonella*. Our data demonstrate that NO congeners prevent *Salmonella* from mounting a functional ATR. The importance of the RNS-mediated repression of the ATR is demonstrated by the fact that NO not only suppresses the increased oral virulence of *Salmonella* preadapted in a mild acidic environment, but it also decreases fecal shedding. NO-mediated inhibition of the ATR is likely to contribute to the antimicrobial activity of nitrogen oxides that are either added as food preservatives or concentrated in gastric juice from enterosalivary circulation.

Acidified RNS prevent the induction of the *Salmonella* ATR as demonstrated by the fact that the survival advantage exhibited by *Salmonella* pre-adapted in mildly acidic conditions could not be reversed upon subsequent RNS treatment. In other words, acidified NO congeners cannot repress a pre-established ATR, strongly suggesting that RNS target ATR signaling cascades but not their effectors. The NO-mediated inhibition of the log phase ATR appears to be associated with interference of PhoPQ signaling. This idea is supported by the fact that the hypersensitivity of the *ΔphoQ* mutant to acid stress was not further enhanced by RNS treatment. Moreover, complementation of *ΔphoQ* with a wild-type allele not only restored the ability of rapidly growing *Salmonella* to mount a protective ATR, but also reestablished its sensitivity to NO. Translational analysis revealed decreased levels of both PhoP and PhoQ upon RNS treatment. Expression levels of *phoP* were consistently higher than those of *phoQ*, perhaps reflecting higher basal levels of *phoP* expression from the PhoQ-independent constitutive promoter [51]. The reduced expression of *phoQ* may also be associated with the characteristic mRNA instability of sensory histidine kinases [52]. Together, the decreased levels of PhoP and PhoQ are likely to be responsible for the overall changes in transcription of the PhoP regulon seen after RNS treatment. RNS overwhelmingly inhibited transcription of PhoP-activated genes. Induction of several PhoP-repressed genes including the SPI-1 transcriptional activator *hilA*, and *fliA* and *fliC* components of the flagellar type III secretion systems [47], [53], [54] further support a model in which RNS target PhoPQ signaling. However, our data have not revealed whether the inhibition of PhoPQ signaling is a consequence of NO-mediated modifications of the sensor kinase or response regulator or is indirectly related to the interference of RNS with upstream signaling cascades. The PhoPQ two-component regulatory system coordinates acid resistance in organisms as diverse as the enteropathogens *Salmonella enterica, Escherichia coli* and *Yersinia*, and the plant pathogen *Erwinia chrysanthemi* [48], [55]–[57]. Therefore, RNS-mediated inhibition of PhoPQ signaling may form an intrinsic component of the host response against a multitude of bacteria.

*Salmonella* could in turn sense RNS in the gastric lumen to activate an adaptive response that promotes fitness. According to this idea, our studies have revealed that nitrogen oxides stimulate secondary lines of acid resistance by derepressing the Fur regulon. Although Fur appears to contribute to the ATR independently from its role in iron acquisition [23], it remains possible that nitrosylation of iron prosthetic groups of Fur in the nitrosative environment of the stomach may induce the ATR. Support for this hypothesis stems from the fact that Fur-regulated genes were induced in response to acidified RNS and by the fact that in the absence of Fur, NO-treated *Salmonella* were completely killed after 1 h of acid challenge. Nitrogen oxides encountered in the stomach may also help *Salmonella* express virulence factors needed in lower parts of the gastrointestinal tract. The derepression of motility, adherence and invasion genes seen after exposure of *Salmonella* to RNS could foster the ability of *Salmonella* to colonize the small intestine. Despite promoting expression of genes involved in colonization of lower parts of the gastrointestinal tract while derepressing Fur-regulated secondary lines of acid defense, our studies indicate that the overwhelming role of RNS is to decrease *Salmonella* fitness. Accordingly, RNS prevented the gain in oral virulence seen upon adaptation of *Salmonella* in mildly acidic conditions.

NO inhibited respiration in our ATR assay conditions (fig. 4C), likely reflecting the temporal nitrosylation of metal centers in terminal cytochrome oxidases *bo* and *bd* [44], [58], [59]. Unexpectedly, our studies discovered that acidified RNS also repress transcription of the *nuo*, *sdh* and *cyo* operons encoding complexes I, II and III of the ETC, as well as the *atp* operon coding for the F_0_F_1_ ATPase. The two pronged inhibition of the ETC and F_0_F_1_ ATPase is likely to contribute to the acid sensitivity of RNS-treated bacteria, since the buffering capacity associated with the F_0_F_1_ ATPase working in reverse and the proton-translocating components of the ETC will be compromised. In accord with this hypothesis, *atp* mutants have been shown to be extraordinarily susceptible to acid stress [20], [21]. Inhibition of the ETC may have contributed to the decreased survival in EG medium, pH 3.0 of nonadapted *Salmonella* that were pretreated with NO during culture in EG medium, pH 7.0.

The PhoPQ signaling serves multiple roles in the pathogenic cycle of *Salmonella*. In addition to playing a critical role in the ATR, the PhoPQ two component regulatory system promotes resistance to antimicrobial peptides and oxidiative stress [60], [61], while controlling intracellular survival of *Salmonella* within professional phagocytes [62], [63]. The repression of PhoPQ signaling by acidified RNS reported here may also affect *Salmonella* residing within IFNγ-primed macrophages, because activated phagocytes sustain nitrosative chemistry similar to that found in the stomach [64]. Of interest, SPI-2 transcription was equally repressed by nitrosative stress of IFNγ-primed phagocytes [41] or acidified nitrogen oxides that are typically generated in the gastric juice (work herein). Since PhoPQ signaling has been associated with transcription of SPI-2 genes [65]–[67], the studies presented herein raise the intriguing possibility that RNS-inhibitable PhoPQ signaling may contribute to the repression of SPI-2 genes by the nitrosative stress engendered either in the gastric lumen or in the phagosome of IFNγ-primed phagocytes [64]. Future investigations will be needed in order to clarify this possibility.

In summary, our studies are consistent with a model in which RNS contribute to the innate host defenses of the stomach via the targeted inhibition of the PhoPQ-dependent ATR. Because PhoPQ-regulated acid resistance is conserved in multiple enteric pathogens, the RNS-mediated inhibition of PhoPQ signaling may represent a general antimicrobial mechanism of the innate gastric barrier.

## Supporting Information

Table S1 (0.76 MB DOC)

Table S2 (0.07 MB DOC)

Table S3 (0.11 MB DOC)

Table S4 (0.04 MB DOC)
