# Supplementary material for: Nitric Oxide Antagonizes the Acid Tolerance Response that Protects Salmonella against Innate Gastric Defenses
Source: PLoS One. 2008 Mar 19;3(3):e1833. doi: 10.1371/journal.pone.0001833 (PMC2266805; doi:10.1371/journal.pone.0001833)
Supplement: Table S1 — (0.76 MB DOC) [file pone.0001833.s001.doc]

**Table S1**

***Salmonella* genes differentially expressed ≥ 2-fold in response to RNS under adapting pH 4.4**

Genes upregulated

| **STM gene ID no.** | **Gene Name** | **Description** | **Fold change** | **SD** |
| --- | --- | --- | --- | --- |
| STM1808 | *STM1808* | putative cytoplasmic protein | 257.116 | 0.001 |
| STM4399 | *ytfE* | putative cell morphogenesis | 188.629 | 0.002 |
| STM2777 | *iroN* | TonB-dependent siderophore receptor protein | 179.205 | 0.002 |
| STM4552 | *STM4552* | putative inner membrane protein | 141.518 | 0.006 |
| STM0727 | *STM0727* | putative cytoplasmic protein | 81.421 | 0.304 |
| STM2556 | *hmpA* | dihydropteridine reductase 2 and nitric oxide dioxygenase activity | 72.095 | 0.005 |
| STM2698 | *STM2698* | Fels-2 prophage: similar to gpE in phage P2 | 64.482 | 0.303 |
| STM1271 | *yeaR* | putative cytoplasmic protein | 55.981 | 0.007 |
| STM4215 | *STM4215* | putative cytoplasmic protein | 54.270 | 0.256 |
| STM1092 | *orfX* | putative cytoplasmic protein | 53.441 | 0.300 |
| STM1214 | *ycfR* | putative outer membrane protein | 46.557 | 0.008 |
| STM1250 | *STM1250* | putative cytoplasmic protein | 45.266 | 0.031 |
| STM3361 | *yhcN* | putative outer membrane protein | 39.242 | 0.018 |
| STM3911 | *STM3911* | putative inner membrane protein | 38.708 | 0.172 |
| STM2804 | *STM2804* | putative cytoplasmic protein | 35.997 | 0.056 |
| STM2861 | *sitA* | Salmonella iron transporter: fur regulated | 34.441 | 0.004 |
| STM0299 | *safA* | putative outer membrane protein | 31.198 | 0.099 |
| STM2798 | *ygaP* | putative rhodanese-related sulfurtransferase | 31.021 | 0.022 |
| STM1882 | *yebG* | DNA damage-inducible gene in SOS regulon, dependent on cyclic AMP and H-NS | 28.732 | 0.007 |
| STM1705 | *osmB* | osmotically inducible lipoprotein | 27.118 | 0.034 |
| STM2805 | *nrdH* | glutaredoxin-like protein; hydrogen donor | 26.699 | 0.027 |
| STM1722 | *trpL* | trp operon leader peptide | 25.908 | 0.532 |
| STM2773 | *iroB* | putative glycosyl transferase, related to UDP-glucuronosyltransferase | 25.237 | 0.027 |
| STM4240 | *yjbJ* | putative cytoplasmic protein | 24.853 | 0.055 |
| STM2797 | *STM2797* | putative regulatory protein, arsR family | 22.794 | 0.069 |
| STM1251 | *STM1251* | putative molecular chaperone (small heat shock protein) | 22.031 | 0.003 |
| STM1071 | *sulA* | suppressor of lon; inhibitor of cell division and FtsZ ring formation upon DNA damage/inhibition, HslVU and Lon involved in its turnover | 21.480 | 0.010 |
| STM2862 | *sitB* | Salmonella iron transporter: fur regulated | 18.677 | 0.007 |
| STM2983 | *ygdI* | putative lipoprotein | 17.591 | 0.054 |
| STM3362 | *STM3362* | putative periplasmic protein | 17.151 | 0.037 |
| STM2408 | *mntH* | Nramp family, manganese/divalent cation transport prortein | 17.011 | 0.010 |
| STM0759 | *ybgS* | putative homeobox protein | 16.954 | 0.022 |
| STM2860 | *ygbA* | putative cytoplasmic protein | 16.816 | 0.076 |
| STM3325 | *yrbL* | putative cytoplasmic protein | 16.790 | 0.022 |
| STM3777 | *STM3777* | putative cytoplasmic protein | 16.664 | 0.028 |
| STM3236 | *yhaK* | putative cytoplasmic protein | 16.424 | 0.011 |
| STM0474 | *ybaJ* | putative cytoplasmic protein | 15.506 | 0.025 |
| STM1272 | *yoaG* | putative cytoplasmic protein | 15.332 | 0.133 |
| STM0378 | *yaiY* | putative inner membrane protein | 15.053 | 0.068 |
| STM0823 | *ybiJ* | putative periplasmic protein | 14.817 | 0.046 |
| STM0467 | *ffs* | signal recognition particle, RNA component | 14.732 | 0.146 |
| STM2806 | *nrdI* | stimulates ribonucleotide reduction | 14.177 | 0.022 |
| STM3941 | *STM3941* | putative inner membrane protein | 13.771 | 0.392 |
| STM0200 | *stfG* | putative minor fimbrial subunit; putative adhesin | 13.722 | 0.234 |
| STM4276 | *STM4276* | putative cytoplasmic protein | 13.663 | 0.279 |
| STM4440 | *STM4440* | putative cytoplasmic protein | 13.607 | 0.136 |
| STM1212 | *ycfJ* | putative outer membrane lipoprotein | 13.588 | 0.036 |
| STM3809 | *ibpA* | small heat shock protein | 13.416 | 0.008 |
| STM2407 | *ypeC* | putative periplasmic protein | 13.409 | 0.101 |
| STM3080 | *STM3080* | putative mannitol dehydrogenase | 13.406 | 0.217 |
| STM1130 | *STM1130* | putative inner membrane protein | 13.311 | 0.097 |
| STM1019 | *STM1019* | Gifsy-2 prophage | 13.070 | 0.010 |
| STM2630 | *STM2630* | Gifsy-1 prophage | 13.061 | 0.081 |
| STM0501 | *ybbK* | putative inner membrane protein | 13.041 | 0.035 |
| STM3517 | *STM3517* | putative DNA-damage-inducibile protein, resembles dinJ | 13.009 | 0.044 |
| STM1926 | *STM1926* | putative cytoplasmic protein | 12.988 | 0.256 |
| STM3778 | *STM3778* | putative helix-turn-helix protein | 12.467 | 0.030 |
| STM2400 | *STM2400* | putative inner membrane protein | 12.267 | 0.049 |
| STM1998 | *umuD* | error-prone repair: SOS-response transcriptional repressors (LexA homologs, RecA-mediated autopeptidases) | 11.898 | 0.058 |
| STM1249 | *STM1249* | putative periplasmic protein | 11.826 | 0.024 |
| STM1809 | *STM1809* | putative cytoplasmic protein | 11.786 | 0.056 |
| STM4124 | *oxyS* | stable RNA induced by oxidative stress | 11.582 | 0.266 |
| STM3808 | *ibpB* | small heat shock protein | 11.515 | 0.010 |
| STM4575 | *STM4575* | putative outer membrane protein | 11.210 | 0.020 |
| STM4031 | *STM4031* | putative cytoplasmic protein | 11.190 | 0.098 |
| STM4218 | *STM4218* | putative inner membrane protein | 11.072 | 0.384 |
| STM0614 | *ybdQ* | putative Universal stress protein UspA and related nucleotide-binding protein | 11.034 | 0.093 |
| STM1787 | *STM1787* | hydrogenase-1 large subunit | 10.709 | 0.322 |
| STM2717 | *STM2717* | Fels-2 prophage: similar to gpX in phage P2 | 10.593 | 0.175 |
| STM2733 | *STM2733* | Fels-2 prophage: similar to E. coli retron Ec67 | 10.593 | 0.210 |
| STM4573 | *stjC* | putative fimbrial chaparone protein | 10.415 | 0.242 |
| STM3516 | *STM3516* | putative cytoplasmic protein | 10.337 | 0.085 |
| STM1360 | *ydiN* | putative MFS family transport protein | 10.273 | 0.339 |
| STM3552 | *yhhA* | putative outer membrane protein | 10.095 | 0.036 |
| STM4237 | *lexA* | SOS response regulator, transcriptional repressor (LexA family) | 10.071 | 0.029 |
| STM2515 | *ratA* | putative outer membrane protein | 9.983 | 0.041 |
| STM4528 | *STM4528* | putative inner membrane protein | 9.957 | 0.065 |
| STM0905 | *STM0905* | Fels-1 prophage | 9.931 | 0.293 |
| STM2807 | *nrdE* | ribonucleoside diphosphate reductase 2, alpha subunit | 9.872 | 0.030 |
| PSLT102 | *traS* | conjugative transfer: surface exclusion | 9.853 | 0.034 |
| STM4424 | *STM4424* | putative endonuclease | 9.841 | 0.216 |
| STM2808 | *nrdF* | ribonucleoside-diphosphate reductase 2, beta subunit | 9.742 | 0.041 |
| STM2401 | *ddg* | cold shock-induced palmitoleoyl transferase | 9.682 | 0.081 |
| STM1141 | *csgE* | curli production assembly/transport component, 2nd curli operon | 9.506 | 0.363 |
| PSLT107 | *PSLT107* | putative cytoplasmic protein | 9.289 | 0.061 |
| STM2132 | *STM2132* | pseudogene; frameshift | 9.263 | 0.250 |
| STM3135 | *STM3135* | putative mannonate hydrolase | 9.199 | 0.037 |
| STM2186 | *STM2186* | putative NADPH-dependent glutamate synthase beta chain or related oxidoreductase | 9.163 | 0.428 |
| STM3688 | *STM3688* | putative cytoplasmic protein | 8.947 | 0.217 |
| STM0283 | *STM0283* | putative inner membrane protein | 8.767 | 0.504 |
| STM4574 | *STM4574* | putative outer membrane protein | 8.763 | 0.025 |
| STM1161 | *yceP* | putative cytoplasmic protein | 8.743 | 0.182 |
| STM3677 | *sgbE* | L-ribulose-5-phosphate 4-epimerase | 8.723 | 0.327 |
| STM0945 | *clpA* | ATP-binding subunit of serine protease | 8.669 | 0.028 |
| STM3590 | *uspB* | universal stress protein B, involved in stationary-phase resistance to ethanol | 8.605 | 0.034 |
| STM2730 | *STM2730* | Fels-2 prophage: similar to retron in E coli | 8.564 | 0.199 |
| STM2863 | *sitC* | Salmonella iron transporter: fur regulated | 8.526 | 0.025 |
| STM1285 | *yeaG* | putative Ser protein kinase | 8.481 | 0.012 |
| STM1959 | *fliC* | flagellar biosynthesis; flagellin, filament structural protein | 8.475 | 0.027 |
| STM2751 | *STM2751* | putative PTS enzyme III glucitol | 8.408 | 0.194 |
| STM2684 | *recN* | protein used in recombination and DNA repair | 8.287 | 0.036 |
| STM2734 | *STM2734* | Fels-2 prophage: hypothetical protein | 8.280 | 0.192 |
| STM2944 | *ygcB* | putative helicase | 8.279 | 0.148 |
| STM1020 | *STM1020* | Gifsy-2 prophage | 8.267 | 0.088 |
| STM1132 | *STM1132* | putative sugar transort protein | 8.180 | 0.360 |
| STM1782 | *ychH* | putative inner membrane protein | 8.118 | 0.086 |
| STM0520 | *STM0520* | putative permease | 8.116 | 0.287 |
| STM2726 | *STM2726* | Fels-2 prophage: hypothetical protein | 8.060 | 0.195 |
| STM0313 | *dinP* | DNA polymerase IV, devoid of proofreading, damage-inducible protein P | 8.031 | 0.050 |
| PSLT105 | *trbH* | conjugative transfer | 7.989 | 0.061 |
| STM1652 | *ynaF* | putative universal stress protein | 7.984 | 0.010 |
| STM3083 | *STM3083* | putative Mannitol dehydrogenase | 7.912 | 0.328 |
| STM1240 | *envF* | putative envelope lipoprotein | 7.857 | 0.198 |
| STM1101 | *hpaG* | 4-hydroxyphenylacetate catabolism | 7.795 | 0.180 |
| STM0470 | *rpmJ2* | putative 50S ribosomal protein L36 (second copy) | 7.672 | 0.220 |
| STM1881 | *yebF* | putative periplasmic protein | 7.541 | 0.030 |
| STM1994 | *STM1994* | putative inner membrane protein | 7.462 | 0.076 |
| STM2718 | *STM2718* | Fels-2 prophage: similar to gpQ in phage 186 | 7.434 | 0.243 |
| STM1268 | *STM1268* | putative cytoplasmic protein | 7.400 | 0.206 |
| STM2732 | *STM2732* | Fels-2 prophage: similar to protein in phage 186 | 7.366 | 0.202 |
| STM0473 | *hha* | hemolysin expression modulating protein (involved in environmental regulation of virulence factors) | 7.338 | 0.015 |
| STM0144 | *ppdD* | putative major component of type IV pilin, prelipin peptidase dependent protein | 7.298 | 0.132 |
| STM4401 | *ytfG* | paral putative reductase | 7.243 | 0.239 |
| STM4429 | *STM4429* | putative cytoplasmic protein | 7.205 | 0.175 |
| STM3906 | *STM3906* | putative cytoplasmic protein | 7.113 | 0.028 |
| STM4435 | *STM4435* | putative cytoplasmic protein | 7.097 | 0.174 |
| STM1308 | *spy* | periplasmic protein related to spheroblast formation | 7.095 | 0.024 |
| STM4076 | *ydeZ* | putative ABC superfamily (membrane), sugar transport protein | 7.052 | 0.104 |
| STM3953 | *yigF* | putative inner membrane protein | 7.047 | 0.308 |
| STM2703 | *aSTM2703* | Fels-2 prophage: similar to invertase (pin) in phage E14 | 6.957 | 0.221 |
| STM1564 | *yddX* | putative cytoplasmic protein | 6.922 | 0.221 |
| STM4442 | *STM4442* | putative cytoplasmic protein | 6.897 | 0.194 |
| STM3575 | *yhhN* | putative inner membrane protein | 6.860 | 0.019 |
| STM4436 | *STM4436* | putative endonuclease | 6.830 | 0.411 |
| STM2044 | *pduH* | Propanediol utilization: diol dehydratase reactivation | 6.800 | 0.314 |
| STM2189 | *mglA* | ABC superfamily (atp_bind), galactose (methyl-galactoside) transport protein | 6.785 | 0.242 |
| STM0278 | *STM0278* | putative periplasmic protein | 6.729 | 0.163 |
| STM1355 | *ydiP* | putative transcription regulator, AraC family | 6.662 | 0.211 |
| STM2774 | *iroC* | putative ATP binding cassette (ABC) transporter | 6.661 | 0.089 |
| STM3762 | *cigR* | putative inner membrane protein | 6.660 | 0.064 |
| PSLT055 | *samA* | mutagenesis by UV and mutagens; related to umuDC operon | 6.654 | 0.033 |
| PSLT106 | *PSLT106* | homologue of mvpA, Shigella flexneri | 6.636 | 0.059 |
| STM4461 | *pyrL* | pyrBI operon leader peptide | 6.603 | 0.213 |
| STM2649 | *trxC* | thioredoxin 2, redox factor | 6.556 | 0.046 |
| STM1007 | *STM1007* | Gifsy-2 prophage | 6.553 | 0.195 |
| STM2864 | *sitD* | Salmonella iron transporter: fur regulated | 6.550 | 0.046 |
| STM3510 | *yhgH* | putative amidophosphoribosyltransferase | 6.546 | 0.029 |
| STM2101 | *wcaK* | putative galactokinase in colanic acid gene cluster | 6.540 | 0.230 |
| STM4434 | *STM4434* | putative permease | 6.528 | 0.478 |
| STM2135 | *STM2135* | putative inner membrane protein | 6.491 | 0.084 |
| STM4030 | *STM4030* | putative cytoplasmic protein | 6.481 | 0.096 |
| STM1128 | *STM1128* | putative sodium/glucose cotransporter | 6.397 | 0.046 |
| STM4402 | *ytfH* | putative transcriptional regulator | 6.367 | 0.023 |
| STM0550 | *fimY* | putative regulatory protein | 6.366 | 0.208 |
| STM0275 | *STM0275* | putative cytoplasmic protein | 6.351 | 0.205 |
| STM0300 | *safB* | putative fimbriae assembly chaparone | 6.337 | 0.196 |
| STM0586 | *fes* | enterochelin esterase | 6.286 | 0.063 |
| STM1104 | *hpaF* | 4-hydroxyphenylacetate catabolism | 6.263 | 0.517 |
| STM0531 | *ylbF* | putative cytoplasmic protein | 6.253 | 0.115 |
| STM0725 | *STM0725* | putative glycosyltransferase, cell wall biogenesis | 6.213 | 0.220 |
| STM4216 | *STM4216* | putative inner membrane protein | 6.203 | 0.308 |
| STM4054 | *STM4054* | putative dicarboxylate-binding periplasmic protein | 6.194 | 0.135 |
| STM2607 | *STM2607* | Gifsy-1 prophage: similar to head to tail joining protein | 6.180 | 0.114 |
| STM2179 | *STM2179* | putative sugar transporter | 6.160 | 0.167 |
| STM3052 | *STM3052* | putative outer membrane protein | 6.093 | 0.053 |
| STM4045 | *rhaD* | rhamnulose-1-phosphate aldolase | 6.067 | 0.057 |
| STM2701 | *STM2701* | Fels-2 prophage: similar to tail sheath proteins in phage P2 | 6.066 | 0.455 |
| STM1369 | *sufA* | putative HesB-like domain | 6.044 | 0.021 |
| STM1010 | *aSTM2631* | Gifsy-1 prophage | 6.029 | 0.122 |
| STM3690 | *STM3690* | putative inner membrane lipoprotein | 6.006 | 0.192 |
| STM1129 | *STM1129* | putative inner membrane protein | 6.006 | 0.282 |
| STM4032 | *STM4032* | putative acetyl esterase | 5.968 | 0.076 |
| STM3138 | *STM3138* | putative methyl-accepting chemotaxis protein | 5.958 | 0.291 |
| STM2595 | *STM0917* | Fels-1 prophage; putative minor tail protein | 5.934 | 0.098 |
| STM0772 | *gpmA* | phosphoglyceromutase 1 | 5.927 | 0.023 |
| STM1553 | *STM1553* | pseudogene; frameshift | 5.922 | 0.088 |
| STM1239 | *STM1239* | putative cytoplasmic protein | 5.914 | 0.164 |
| STM2783 | *nxiA* | putative nickel transporter | 5.907 | 0.033 |
| STM0037 | *STM0037* | putative cytoplasmic protein | 5.879 | 0.266 |
| STM4013 | *STM4013* | putative membrane-associated, metal-dependent hydrolase | 5.871 | 0.069 |
| STM1011 | *STM1011* | Gifsy-2 prophage | 5.860 | 0.083 |
| STM1140 | *csgF* | curli production assembly/transport component, 2nd curli operon | 5.823 | 0.084 |
| STM2764 | *STM2764* | putative integrase core domain | 5.815 | 0.198 |
| STM4087 | *glpF* | MIP channel, glycerol diffusion | 5.815 | 0.108 |
| STM2977 | *fucK* | L-fuculokinase | 5.784 | 0.147 |
| STM1912 | *flhE* | flagellar protein | 5.773 | 0.299 |
| STM2720 | *STM2720* | Fels-2 prophage: similar to gpN, major capsid, in phage P2 | 5.764 | 0.429 |
| STM0374 | *yaiV* | putative inner membrane protein | 5.740 | 0.140 |
| STM0573 | *STM0573* | putative inner membrane protein | 5.705 | 0.244 |
| STM0009 | *yaaH* | putative regulator | 5.700 | 0.390 |
| STM2832 | *srlA* | PTS family, glucitol/sorbitol-specific enzyme IIC component,one of two IIC components | 5.666 | 0.199 |
| STM2118 | *wza* | putative polysaccharide export protein, outer membrane | 5.640 | 0.227 |
| STM0561 | *STM0561* | Sensor protein | 5.624 | 0.164 |
| STM2904 | *STM2904* | putative ABC-type transport system | 5.623 | 0.021 |
| STM1494 | *STM1494* | ABC-type transport systems, permease components | 5.582 | 0.199 |
| STM4425 | *STM4425* | putative dehydrogenase | 5.565 | 0.174 |
| STM4229 | *malE* | ABC superfamily (bind_prot) maltose transport protein, substrate recognition for transport and chemotaxis | 5.558 | 0.168 |
| STM4060 | *cpxP* | periplasmic repressor of cpx regulon by interaction with CpxA, rescue from transitory stresses | 5.556 | 0.173 |
| STM0831 | *dps* | stress response DNA-binding protein; starvation induced resistance to H2O2 | 5.511 | 0.045 |
| STM2695 | *STM2695* | Fels-2 prophage: similar to late control gene in phage | 5.502 | 0.166 |
| STM4508 | *trpS2* | putative tryptophanyl-tRNA synthetase | 5.498 | 0.042 |
| STM3077 | *yggG* | putative Zn-dependent proteases with possible chaperone function | 5.495 | 0.168 |
| STM2888 | *spaR* | surface presentation of antigens; secretory proteins | 5.477 | 0.230 |
| STM4053 | *STM4053* | putative C4-dicarboxylate transport system | 5.448 | 0.278 |
| STM2700 | *STM2700* | Fels-2 prophage: similar to genes in P2-like phages | 5.412 | 0.258 |
| STM0944 | *yljA* | putative cytoplasmic protein | 5.411 | 0.071 |
| STM3016 | *araE* | MFS family, L-arabinose: proton symport protein (low-affinity transporter) | 5.405 | 0.142 |
| STM3022 | *STM3022* | putative transport protein | 5.373 | 0.080 |
| STM2768 | *STM2768* | putative transposase | 5.364 | 0.175 |
| STM0008 | *mog* | putative molybdochetalase in molybdopterine biosynthesis | 5.363 | 0.038 |
| STM1551 | *STM1551* | putative cytoplasmic protein | 5.352 | 0.055 |
| STM3995 | *yihD* | putative cytoplasmic protein | 5.337 | 0.070 |
| STM1997 | *umuC* | error-prone repair: component of DNA polymerase V with UmuD' | 5.317 | 0.065 |
| STM0330 | *STM0330* | putative 3-isopropylmalate isomerase (dehydratase), subunit with LeuC | 5.298 | 0.119 |
| STM0665 | *gltI* | ABC superfamily (bind_prot), glutamate/aspartate transporter | 5.294 | 0.047 |
| STM0277 | *STM0277* | putative cytoplasmic protein | 5.290 | 0.187 |
| STM1178 | *flgF* | flagellar biosynthesis, cell-proximal portion of basal-body rod | 5.289 | 0.276 |
| STM4408 | *msrA* | peptide methionine sulfoxide reductase | 5.279 | 0.077 |
| STM4257 | *STM4257* | putative inner membrane or exported | 5.279 | 0.181 |
| STM3476 | *nirC* | FNT family, nitrite transport protein | 5.270 | 0.332 |
| STM2231 | *STM2231* | homologue of msgA; ssrB-regulated factor | 5.255 | 0.128 |
| STM4482 | *idnT* | GntP family, L-idonate transport protein | 5.250 | 0.201 |
| STM0897 | *STM0897* | Fels-1 prophage | 5.234 | 0.193 |
| STM2775 | *iroD* | Similar to enterochelin esterase of E. coli (Fes) | 5.233 | 0.301 |
| STM0853 | *yliH* | putative cytoplasmic protein | 5.218 | 0.032 |
| STM3081 | *STM3081* | putative malate/L-lactate dehydrogenase | 5.210 | 0.254 |
| STM3255 | *STM3255* | putative phosphotransferase system fructose-specific component IIB | 5.200 | 0.220 |
| STM1305 | *astD* | succinylglutamic semialdehyde dehydrogenase | 5.198 | 0.244 |
| STM4259 | *STM4259* | putative ABC exporter outer membrane component homolog | 5.194 | 0.121 |
| STM4266 | *soxR* | redox-sensing transcriptional activator SoxR, contains iron-sulfur center for redox-sensing (MerR family) | 5.185 | 0.047 |
| STM1916 | *cheY* | chemotaxis regulator, transmits chemoreceptor signals to flagelllar motor components | 5.163 | 0.167 |
| STM2007 | *STM2007* | putative TPR repeat protein | 5.163 | 0.136 |
| STM0577 | *STM0577* | putative transport protein, PTS system | 5.144 | 0.131 |
| STM2664 | *STM2664* | pseudogene; two in-frame stops relative to E. coli b2596 | 5.128 | 0.198 |
| STM0017 | *STM0017* | putative protein | 5.122 | 0.197 |
| STM2771 | *fljB* | Flagellar synthesis: phase 2 flagellin (filament structural protein) | 5.109 | 0.134 |
| STM0521 | *ybbV* | putative cytoplasmic protein | 5.103 | 0.290 |
| STM0343 | *STM0343* | putative Diguanylate cyclase/phosphodiesterase domain 1 | 5.084 | 0.206 |
| STM4253 | *STM4253* | putative outer membrane lipoprotein | 5.078 | 0.189 |
| STM2997 | *ppdC* | prepilin peptidase dependent protein C, putative component in type IV pilin biogenesis | 5.015 | 0.163 |
| STM4593 | *sthB* | putative fimbrial usher protein | 4.989 | 0.371 |
| STM0731 | *STM0731* | putative inner membrane protein | 4.974 | 0.029 |
| STM4075 | *ydeY* | putative ABC superfamily (membrane), sugar transport protein | 4.968 | 0.082 |
| STM2220 | *yejG* | putative cytoplasmic protein | 4.949 | 0.049 |
| STM4260 | *STM4260* | membrane permease, predicted cation efflux pump | 4.941 | 0.497 |
| STM3154 | *STM3154* | putative ATP-dependent RNA helicase-like protein | 4.938 | 0.165 |
| STM0020 | *STM0020* | putative cytoplasmic protein | 4.892 | 0.187 |
| STM4529 | *STM4529* | putative cytoplasmic protein | 4.885 | 0.367 |
| STM0830 | *glnH* | ABC superfamily (bind_prot), glutamine high-affinity transporter | 4.885 | 0.013 |
| STM4306 | *STM4306* | putative anaerobic dimethyl sulfoxide reductase, subunit B | 4.879 | 0.261 |
| STM3389 | *envR* | transcriptional repressor for envCD (acrEF) (TetR/AcrR family) | 4.869 | 0.115 |
| STM4454 | *treB* | pseudogene; frameshift | 4.854 | 0.208 |
| STM2705 | *STM2705* | Fels-2 prophage | 4.854 | 0.121 |
| STM0298 | *STM0298* | putative integrase core domain | 4.836 | 0.191 |
| STM2169 | *yohC* | paral putative transport protein | 4.824 | 0.045 |
| STM0552 | *fimW* | putative fimbrial protein | 4.808 | 0.145 |
| STM2031 | *cbiE* | synthesis of vitamin B12 adenosyl cobalamide precursor | 4.805 | 0.321 |
| STM0363 | *STM0363* | putative transcription regulator, AraC family | 4.802 | 0.101 |
| STM3591 | *uspA* | universal stress protein A | 4.799 | 0.119 |
| PSLT018 | *pefA* | plasmid-encoded fimbriae; major fimbrial subunit | 4.798 | 0.124 |
| STM2109 | *gmd* | GDP-D-mannose dehydratase in colanic acid gene cluster | 4.794 | 0.167 |
| STM1382 | *orf408* | putative regulatory protein, deoR family | 4.789 | 0.175 |
| STM2177 | *STM2177* | putative flutathione S-transferase | 4.789 | 0.068 |
| STM1142 | *csgD* | putative transcriptional regulator (LuxR/UhpA family) | 4.783 | 0.135 |
| PSLT028 | *ccdB* | toxin addiction system: toxin | 4.763 | 0.028 |
| STM1306 | *astB* | succinylarginine dihydrolase | 4.760 | 0.130 |
| STM0538 | *STM0538* | putative outer membrane protein | 4.760 | 0.247 |
| STM0560 | *STM0560* | pseudogene; in-frame stop following codon 56 | 4.742 | 0.098 |
| STM3120 | *STM3120* | putative transcriptional regulator, LysR family | 4.734 | 0.039 |
| STM4233 | *ubiC* | chorismate pyruvate lyase | 4.714 | 0.021 |
| STM0295 | *STM0295* | putative cytoplasmic protein | 4.701 | 0.249 |
| PSLT054 | *samB* | mutagenesis by UV and mutagens; related to umuDC operon | 4.690 | 0.052 |
| STM1111 | *yccD* | putative cytoplasmic protein | 4.682 | 0.036 |
| STM2586 | *STM2586* | Gifsy-1 prophage: similar to phage tail assembly protein | 4.678 | 0.158 |
| STM3558 | *STM3558* | homology to death -on-curing protein of phage P1 | 4.669 | 0.156 |
| STM1690 | *pspA* | phage shock protein; negative regulatory gene for the psp opreon | 4.669 | 0.049 |
| STM2631 | *STM2631* | Gifsy-1 prophage | 4.652 | 0.196 |
| STM4251 | *yjbR* | putative cytoplasmic protein | 4.652 | 0.090 |
| STM3780 | *STM3780* | putative fructose-bisphosphate aldolase class-II | 4.630 | 0.157 |
| STM3126 | *STM3126* | putative amino acid transporter | 4.629 | 0.203 |
| STM3982 | *fadA* | 3-ketoacyl-CoA thiolase; (thiolase I, acetyl-CoA transferase), in complex with FadB catalyzes EC 2.3.1.16 reaction | 4.627 | 0.136 |
| STM0472 | *maa* | maltose o-acetyltransferase | 4.622 | 0.108 |
| STM3082 | *STM3082* | putative zinc-binding dehydrogenase | 4.606 | 0.181 |
| STM0074 | *caiT* | putative BCCT family, betaine/carnitine/choline transport protein | 4.589 | 0.229 |
| PSLT087 | *PSLT087* | conjugative transfer: | 4.584 | 0.322 |
| STM1550 | *STM1550* | putative cytoplasmic protein | 4.583 | 0.052 |
| STM3029 | *stdA* | putative fimbrial-like protein | 4.571 | 0.283 |
| STM4419 | *STM4419* | sugar (and other) transporter | 4.564 | 0.147 |
| STM4039 | *STM4039* | putative inner membrane lipoprotein | 4.549 | 0.190 |
| PSLT078 | *traL* | conjugative transfer: assembly | 4.512 | 0.207 |
| STM1661 | *ydaA* | putative universal stress protein | 4.489 | 0.013 |
| STM4250 | *yjbQ* | putative cytoplasmic protein | 4.487 | 0.073 |
| STM3028 | *stdB* | putative outer membrane usher protein | 4.483 | 0.173 |
| STM2876 | *hilA* | invasion genes transcription activator | 4.473 | 0.413 |
| STM2769 | *STM2769* | putative transposase | 4.468 | 0.068 |
| STM0335 | *STM0335* | putative outer membrane protein | 4.453 | 0.232 |
| STM1162 | *dinI* | DNA damage-inducible protein I, inhibits UmuD processing | 4.445 | 0.140 |
| STM1374 | *ynhA* | putative SufE protein probably involved in Fe-S center assembly | 4.442 | 0.066 |
| STM3160 | *STM3160* | putative inner membrane protein | 4.439 | 0.146 |
| STM0902 | *STM0902* | Fels-1 prophage | 4.403 | 0.190 |
| STM2795 | *ygaU* | putative LysM domain | 4.400 | 0.048 |
| STM4194 | *yjbD* | putative cytoplasmic protein | 4.394 | 0.132 |
| STM3945 | *STM3945* | pseudogene; 2 frameshifts | 4.390 | 0.180 |
| STM1081 | *STM1081* | putative outer membrane protein | 4.383 | 0.457 |
| STM1304 | *astA* | arginine succinyltransferase | 4.383 | 0.142 |
| STM3650 | *STM3650* | putative periplasmic or exported protein | 4.378 | 0.083 |
| STM2435 | *pdxK* | pyridoxal-pyridoxamine kinase/hydroxymethylpyrimidine kinase | 4.375 | 0.036 |
| STM0777 | *STM0777* | putative inner membrane protein | 4.368 | 0.332 |
| STM0592 | *fepD* | ABC superfamily (membrane), ferric enterobactin (enterochelin) transporter | 4.349 | 0.228 |
| PSLT027 | *ccdA* | toxin addiction system: antidote | 4.336 | 0.070 |
| STM0324 | *STM0324* | putative inner membrane protein | 4.328 | 0.122 |
| STM4376 | *yjfC* | putative glutathionylspermidine synthase | 4.317 | 0.257 |
| STM0660 | *STM0660* | putative cytoplasmic protein | 4.316 | 0.085 |
| STM3137 | *STM3137* | putative uronate isomerase | 4.306 | 0.073 |
| STM0062 | *citX2* | putative cytoplasmic protein | 4.304 | 0.129 |
| STM0651 | *STM0651* | putative permease | 4.302 | 0.107 |
| STM2198 | *STM2198* | putative regulatory protein | 4.298 | 0.110 |
| STM4378 | *yjfN* | putative inner membrane protein | 4.288 | 0.055 |
| STM2569 | *yfhB* | putatative phosphoserine phosphatase | 4.287 | 0.069 |
| STM0721 | *STM0721* | putative glycosyl transferase | 4.284 | 0.255 |
| STM0458 | *STM0458* | putative cysteine synthase/cystathionine beta-synthase | 4.264 | 0.138 |
| STM0011 | *yaaI* | putative periplasmic protein | 4.261 | 0.136 |
| STM4418 | *STM4418* | sugar (and other) transporter | 4.259 | 0.060 |
| STM4086 | *glpK* | glycerol kinase | 4.255 | 0.101 |
| STM3957 | *pldA* | outer membrane phospholipase A | 4.244 | 0.093 |
| STM4181 | *yjaB* | putative acetyltransferase | 4.230 | 0.078 |
| STM2484 | *STM2484* | putative inner membrane protein | 4.227 | 0.048 |
| STM1145 | *csgC* | putative curli production protein | 4.209 | 0.186 |
| PSLT082 | *traP* | conjugative transfer | 4.157 | 0.176 |
| STM1373 | *sufS* | selenocysteine lyase | 4.147 | 0.058 |
| STM2886 | *sicA* | surface presentation of antigens; secretory proteins | 4.140 | 0.105 |
| STM0010 | *htgA* | positive regulator for sigma H (sigma 32) promoters, permitting growth at high temperature | 4.134 | 0.116 |
| STM4403 | *cpdB* | 2':3'-cyclic-nucleotide 2'-phosphodiesterase | 4.134 | 0.077 |
| STM2776 | *iroE* | putative hydrolase of the alpha/beta superfamily | 4.129 | 0.085 |
| STM0936 | *hcr* | NADH oxidoreductase for hcp gene product | 4.125 | 0.086 |
| STM0085 | *yabF* | putative NAD(P)H oxidoreductase | 4.116 | 0.095 |
| STM2905 | *STM2905* | putative acetyltransferase | 4.104 | 0.024 |
| STM1091 | *sopB* | Salmonella outer protein: homologous to ipgD of Shigella | 4.100 | 0.330 |
| STM2462 | *eutJ* | paral putative heatshock protein (Hsp70) | 4.094 | 0.538 |
| STM0081 | *STM0081* | putative secreted protein | 4.093 | 0.116 |
| STM1868 | *mig-3* | Homology to phage-tail assembly proteins | 4.078 | 0.063 |
| STM1689 | *pspB* | phage shock protein; regulatory gene, activates expression of psp operon with PspC | 4.065 | 0.143 |
| STM1927 | *yecG* | putative universal stress protein | 4.063 | 0.027 |
| STM2735 | *STM2735* | Fels-2 prophage: hypothetical protein | 4.052 | 0.228 |
| STM4514 | *yjiH* | putative inner membrane protein | 4.050 | 0.128 |
| STM1981 | *fliR* | putative flagellar biosynthetic protein | 4.031 | 0.157 |
| STM3166 | *STM3166* | putative cation transporter | 4.026 | 0.084 |
| STM1505 | *rspA* | putative dehydratase, starvation sensing protein | 4.009 | 0.178 |
| STM2178 | *STM2178* | putative 1,2-dioxygenase protein | 4.001 | 0.131 |
| PSLT072 | *PSLT072* | putative transglycosylase | 3.998 | 0.074 |
| STM1869 | *STM1869* | Homology to phage-tail assembly proteins | 3.996 | 0.231 |
| PSLT056 | *PSLT056* | putative cytoplasmic protein | 3.990 | 0.122 |
| STM2292 | *yfaX* | putative transcriptional regulator | 3.988 | 0.086 |
| STM0630 | *crcB* | high-copy crc-csp restores normal chromosome condensation in presence of camphor or mukB mutations | 3.977 | 0.051 |
| STM3544 | *yhhW* | putative cytoplasmic protein | 3.975 | 0.054 |
| STM1211 | *ndh* | respiratory NADH dehydrogenase 2; cupric reductase | 3.961 | 0.062 |
| STM2469 | *eutP* | putative ethanolamine utilization protein | 3.953 | 0.319 |
| STM2760 | *STM2760* | putative integrase | 3.952 | 0.178 |
| STM1179 | *flgG* | flagellar biosynthesis, cell-distal portion of basal-body rod | 3.942 | 0.212 |
| STM4396 | *ytfB* | putative cell envelope opacity-associated protein A | 3.933 | 0.028 |
| STM2064 | *phsB* | Hydrogen sulfide production: iron- sulfur subunit; electron transfer | 3.914 | 0.167 |
| STM1918 | *cheR* | glutamate methyltransferase, response regulator for chemotaxis | 3.903 | 0.365 |
| STM1688 | *pspC* | phage shock protein; regulatory gene, activates expression of psp operon with PspB | 3.890 | 0.099 |
| STM2398 | *pgtC* | Phosphoglycerate transport: protein for signal transmission | 3.868 | 0.199 |
| STM0462 | *glnK* | regulatory protein, P-II 2, for nitrogen assimilation by glutamine synthetase, regulates GlnL (NRII) and GlnE (ATase) | 3.860 | 0.015 |
| STM2142 | *yegT* | putative MFS family transport protein | 3.851 | 0.321 |
| STM4008 | *STM4008* | putative cytoplasmic protein | 3.849 | 0.061 |
| STM0434 | *apbA* | ketopantoate reductase | 3.844 | 0.062 |
| PSLT045 | *rlgA* | putative resolvase | 3.843 | 0.087 |
| STM4202 | *STM4202* | putative phage baseplate protein | 3.841 | 0.175 |
| STM2644 | *yfiE* | putative transcriptional regulator, LysR family | 3.835 | 0.068 |
| STM1506 | *rspB* | putative dehydrogenase | 3.834 | 0.115 |
| STM0012 | *dnaK* | chaperone Hsp70 in DNA biosynthesis/cell division | 3.819 | 0.055 |
| STM3159 | *exbB* | uptake of enterochelin; tonB-dependent uptake of B colicins | 3.793 | 0.047 |
| STM1098 | *hpaC* | 4-hydroxyphenylacetate catabolism | 3.791 | 0.045 |
| STM1371 | *sufC* | putative ABC superfamily (atp_bind) transport protein | 3.783 | 0.025 |
| STM0549 | *fimZ* | fimbrial protein Z, putative transcriptional regulator (LuxR/UhpA family) | 3.779 | 0.102 |
| STM0594 | *fepB* | ABC superfamily (peri_perm), ferric enterobactin (enterochelin) tranporter | 3.777 | 0.070 |
| STM4295 | *adiY* | transcriptional activator of adiA (AraC/XylS family) | 3.767 | 0.132 |
| STM4204 | *STM4204* | putative inner membrane protein | 3.763 | 0.110 |
| STM1015 | *STM1015* | Gifsy-2 prophage; ATPase involved in DNA replication initiation | 3.759 | 0.200 |
| STM2891 | *spaO* | surface presentation of antigens; secretory proteins | 3.759 | 0.315 |
| STM1144 | *csgA* | curlin major subunit, coiled surface structures; cryptic | 3.754 | 0.155 |
| STM2896 | *invA* | invasion protein | 3.745 | 0.135 |
| STM4516 | *yjiN* | putative inner membrane protein | 3.740 | 0.113 |
| STM0369 | *prpC* | putative citrate synthase | 3.730 | 0.107 |
| STM0097 | *polB* | DNA polymerase II and and 3' --> 5' exonuclease | 3.723 | 0.045 |
| STM1920 | *cheW* | purine-binding chemotaxis protein; regulation | 3.687 | 0.170 |
| STM0525 | *glxK* | glycerate kinase II | 3.673 | 0.091 |
| PSLT026 | *PSLT026* | putative periplasmic protein | 3.672 | 0.120 |
| STM2008 | *STM2008* | putative periplasmic protein | 3.667 | 0.308 |
| STM0018 | *STM0018* | putative exochitinase | 3.665 | 0.172 |
| STM1545 | *STM1545* | putative multidrug efflux protein | 3.652 | 0.164 |
| STM0619 | *citG* | putative modifier of citrate lyase protein | 3.647 | 0.257 |
| STM4011 | *STM4011* | putative inner membrane protein | 3.646 | 0.141 |
| STM0241 | *cutF* | pseudogene; frameshift relative to E. coli cutF (SW:CUTF_ECOLI); copper homeostasis protein | 3.634 | 0.089 |
| STM2740 | *STM2740* | Fels-2 prophage: similar to int protein in phage CP4-57 | 3.625 | 0.166 |
| STM3983 | *fadB* | 3-hydroxyacyl-coA dehydrogenase (EC 1.1.1.35) of 4-enzyme FadB protein | 3.623 | 0.131 |
| STM1346 | *ydiE* | putative cytoplasmic protein | 3.622 | 0.078 |
| STM3559 | *yhhV* | putative cytoplasmic protein | 3.614 | 0.295 |
| STM0912 | *aSTM0912* | Fels-1 prophage; protease subunits of ATP-dependent proteases, ClpP family | 3.613 | 0.092 |
| STM3540 | *yhgN* | putative inner membrane protein | 3.610 | 0.291 |
| STM1174 | *flgB* | flagellar biosynthesis, cell-proximal portion of basal-body rod | 3.603 | 0.177 |
| STM1499 | *STM1499* | putative dimethyl sulphoxide reductase, chain A1 | 3.601 | 0.083 |
| STM0252 | *rrlH* | 23S rRNA | 3.596 | 0.038 |
| STM3623 | *yhjT* | putative inner membrane protein | 3.594 | 0.101 |
| STM4572 | *stjB* | putative fimbrial usher protein | 3.593 | 0.105 |
| PSLT021 | *PSLT021* | pseudogene; two frameshifts relative to E. coli probable site-specific recombinase (SW:INAF_ECOLI) | 3.591 | 0.288 |
| STM0937 | *hcp* | hybrid cluster protein; similar to prismane-protein homolog | 3.591 | 0.100 |
| STM0969 | *ycaM* | putative APC family, amino-acid transporter | 3.591 | 0.153 |
| STM2796 | *yqaE* | putative YqaE family transport protein | 3.590 | 0.040 |
| STM2063 | *phsC* | Hydrogen sulfide production: membrane anchoring protein | 3.586 | 0.085 |
| STM0031 | *STM0031* | putative transcription regulator | 3.579 | 0.168 |
| STM3640 | *lpfA* | long polar fimbria | 3.572 | 0.188 |
| STM4277 | *nrfA* | nitrite reductase periplasmic cytochrome c(552) | 3.564 | 0.256 |
| STM1103 | *hpaD* | 4-hydroxyphenylacetate catabolism | 3.559 | 0.048 |
| STM4507 | *uxuR* | transcriptional repressor for uxu operon | 3.558 | 0.082 |
| STM4518 | *STM4518* | putative inner membrane protein | 3.550 | 0.157 |
| STM3049 | *yqfA* | putative hemolysin | 3.538 | 0.047 |
| STM1675 | *STM1675* | putative short-chain alcohol dehydrogenase | 3.532 | 0.087 |
| STM2866 | *sprB* | transcriptional regulator | 3.524 | 0.186 |
| STM1967 | *STM1967* | putative 50S ribosomal protein | 3.523 | 0.210 |
| STM2894 | *invC* | surface presentation of antigens; secretory proteins | 3.521 | 0.200 |
| STM3343 | *STM3343* | putative cytoplasmic protein | 3.506 | 0.072 |
| STM0730 | *gltA* | citrate synthase | 3.502 | 0.077 |
| STM2161 | *STM2161* | putative inner membrane protein | 3.487 | 0.294 |
| STM4223 | *yjbF* | putative outer membrane lipoprotein | 3.484 | 0.172 |
| STM4114 | *pflD* | putative pyruvate formate lyase II | 3.480 | 0.209 |
| STM4389 | *yjfY* | putative outer membrane protein | 3.479 | 0.085 |
| STM1861 | *aSTM1861* | putative cytoplasmic protein | 3.477 | 0.216 |
| STM1121 | *ymdF* | putative cytoplasmic protein | 3.475 | 0.137 |
| STM2865 | *avrA* | putative inner membrane protein | 3.474 | 0.026 |
| STM3832 | *STM3832* | putative permease | 3.474 | 0.134 |
| STM1896 | *STM1896* | putative cytoplasmic protein | 3.471 | 0.123 |
| STM1245 | *STM1245* | pseudogene; in-frame stop following codon 4 | 3.469 | 0.304 |
| STM0909 | *STM0909* | Fels-1 prophage | 3.463 | 0.187 |
| STM2176 | *STM2176* | putative glutathione S-transferase | 3.461 | 0.031 |
| STM0364 | *foxA* | ferrioxamine receptor | 3.444 | 0.140 |
| STM2233 | *STM2233* | putative cytoplasmic protein | 3.441 | 0.292 |
| STM2202 | *yeiH* | putative inner membrane protein | 3.440 | 0.121 |
| STM3769 | *STM3769* | putative phosphotransferase system enzyme II | 3.440 | 0.176 |
| STM3526 | *glpD* | sn-glycerol-3-phosphate dehydrogenase (aerobic) | 3.432 | 0.058 |
| STM2672 | *yfiN* | putative diguanylate cyclase/phosphodiesterase | 3.429 | 0.084 |
| STM2201 | *yeiE* | putative transcriptional regulator, LysR family | 3.413 | 0.141 |
| STM0240 | *yaeJ* | putative-tRNA hydrolase domain | 3.405 | 0.163 |
| STM3133 | *STM3133* | putative amidohydrolase | 3.399 | 0.098 |
| STM3652 | *STM3652* | putative cytoplasmic protein | 3.395 | 0.102 |
| STM2686 | *yfjF* | putative cytoplasmic protein | 3.391 | 0.351 |
| STM3621 | *yhjR* | putative cytoplasmic protein | 3.390 | 0.100 |
| STM0989 | *STM0989* | mukF protein (killing factor KicB) | 3.390 | 0.070 |
| STM4297 | *melR* | regulator of melibiose operon (AraC/XylS family) | 3.390 | 0.060 |
| STM1277 | *yeaO* | putative cytoplasmic protein | 3.389 | 0.039 |
| STM1012 | *STM1012* | Gifsy-2 prophage; probable regulatory protein | 3.389 | 0.211 |
| STM2789 | *STM2789* | putative cytoplasmic protein | 3.383 | 0.199 |
| STM3365 | *yhcQ* | putative membrane located multidrug resistance protein | 3.373 | 0.177 |
| STM4404 | *cysQ* | affects pool of 3'-phosphoadenosine-5'-phosphosulfate in pathway of sulfite synthesis | 3.369 | 0.041 |
| STM1279 | *yeaM* | putative regulator (AraC/XylS family) | 3.368 | 0.226 |
| STM3882 | *rbsA* | ABC superfamily (atp_bind), D-ribose high-affinity transport protein | 3.367 | 0.267 |
| STM1120 | *ycdF* | pseudogene; in-frame stops following codons 5 and 21 | 3.358 | 0.040 |
| STM3074 | *STM3074* | putative ABC-type cobalt transport system, ATPase component | 3.349 | 0.238 |
| STM2393 | *yfdC* | putative transport | 3.325 | 0.086 |
| STM1813 | *ycgL* | putative cytoplasmic protein | 3.324 | 0.040 |
| STM1370 | *sufB* | putative ABC transporter | 3.320 | 0.053 |
| STM1810 | *STM1810* | putative cytoplasmic protein | 3.319 | 0.242 |
| STM1803 | *dadA* | D-amino acid dehydrogenase subunit | 3.283 | 0.080 |
| STM3281 | *nlpI* | lipoprotein, cell division | 3.281 | 0.035 |
| STM4488 | *STM4488* | putative integrase | 3.272 | 0.083 |
| STM0527 | *allC* | allantoate amidohydrolase | 3.263 | 0.158 |
| STM4597 | *STM4597* | putative periplasmic protein | 3.248 | 0.425 |
| STM2486 | *STM2486* | putative inner membrane protein | 3.241 | 0.035 |
| STM2390 | *yfcZ* | putative cytoplasmic protein | 3.239 | 0.082 |
| STM1788 | *STM1788* | putative Ni/Fe-hydrogenase 1 b-type cytochrome subunit | 3.237 | 0.090 |
| STM1445 | *slyB* | putative outer membrane lipoprotein | 3.235 | 0.034 |
| STM4588 | *creB* | response regulator in two-component regulatory system with CreC (OmpR family) | 3.234 | 0.105 |
| STM2877 | *iagB* | cell invasion protein | 3.234 | 0.151 |
| STM1674 | *STM1674* | putative bacterial regulatory helix-turn-helix proteins, araC family | 3.229 | 0.149 |
| STM2639 | *rseA* | anti sigma E (sigma 24) factor, negative regulator | 3.227 | 0.020 |
| STM1482 | *ydgF* | putative membrane transporter of cations and cationic drugs | 3.220 | 0.210 |
| STM0100 | *STM0100* | putative cytoplasmic protein | 3.215 | 0.113 |
| STM3775 | *STM3775* | putative glycosyl hydrolase family | 3.213 | 0.153 |
| STM4373 | *yjfK* | putative cytoplasmic protein | 3.204 | 0.151 |
| STM3827 | *dgoT* | MFS family, D-galactonate transport protein | 3.198 | 0.287 |
| STM1255 | *STM1255* | putative ABC transporter periplasmic binding protein | 3.191 | 0.118 |
| STM4342 | *frdB* | fumarate reductase, anaerobic, Fe-S protein subunit | 3.191 | 0.158 |
| STM1085 | *yccA* | putative TEGT family carrier/transport protein | 3.185 | 0.023 |
| STM3215 | *yqjI* | putative transcriptional regulator | 3.183 | 0.056 |
| STM0309 | *yafH* | putative acyl-CoA dehydrogenase | 3.180 | 0.145 |
| STM3787 | *uhpT* | MFS family, hexose phosphate transport protein | 3.170 | 0.119 |
| STM2610 | *STM2610* | Gifsy-1 prophage | 3.162 | 0.108 |
| STM3908 | *ilvY* | positive regulator for ilvC (LysR family) | 3.161 | 0.132 |
| STM3355 | *STM3355* | putative tartrate dehydratase alpha subunit | 3.157 | 0.136 |
| STM1791 | *STM1791* | putative hydrogenase-1 protein | 3.150 | 0.148 |
| PSLT101 | *traG* | conjugative transfer: assembly abd aggregate stability | 3.143 | 0.072 |
| STM3687 | *mtlR* | repressor for mtl | 3.142 | 0.107 |
| STM1880 | *yebE* | putative inner membrane protein | 3.140 | 0.117 |
| STM4279 | *nrfC* | putative nitrite reductase; formate-dependent, Fe-S centers | 3.136 | 0.153 |
| STM4258 | *STM4258* | putative methyl-accepting chemotaxis protein | 3.132 | 0.220 |
| STM2833 | *srlE* | PTS family, glucitol/sorbitol-specific IIB component, one of two IIC components | 3.125 | 0.050 |
| STM3771 | *STM3771* | putative phosphotransferase system enzyme IIB | 3.113 | 0.195 |
| STM0566 | *STM0566* | putative inner membrane protein | 3.108 | 0.115 |
| STM0457 | *cof* | putative hydrolase | 3.105 | 0.098 |
| STM0143 | *hofB* | putative integral membrane protein involved in biogenesis of fimbriae (type IV pilin), protein transport, DNA uptake | 3.099 | 0.211 |
| STM1325 | *ydiZ* | putative cytoplasmic protein | 3.092 | 0.051 |
| STM2803 | *STM2803* | putative regulatory protein, gntR family | 3.087 | 0.090 |
| STM2895 | *invB* | surface presentation of antigens; secretory proteins | 3.086 | 0.115 |
| STM1996 | *cspB* | putative cold-shock protein | 3.081 | 0.103 |
| STM3392 | *yhdV* | putative outer membrane lipoprotein | 3.076 | 0.058 |
| STM2406 | *STM2406* | putative oxidoreductase | 3.074 | 0.050 |
| STM1375 | *ynhG* | putative LysM domain | 3.054 | 0.052 |
| STM0500 | *ybbJ* | putative Membrane protein implicated in regulation of membrane protease activity | 3.051 | 0.149 |
| STM3279 | *mtr* | HAAAP family, tryptophan-specific transport protein | 3.049 | 0.131 |
| STM1855 | *sopE2* | TypeIII-secreted protein effector: invasion-associated protein | 3.047 | 0.153 |
| STM3106 | *ansB* | periplasmic L-asparaginase II | 3.036 | 0.180 |
| PSLT098 | *traQ* | conjugative transfer: fimbrial synthesis | 3.034 | 0.169 |
| STM1147 | *STM1147* | putative ACR related to the C-terminal domain of histone macroH2A1 | 3.032 | 0.092 |
| STM2840 | *STM2840* | putative flavoprotein | 3.032 | 0.105 |
| PSLT084 | *traV* | conjugative transfer: assembly | 3.031 | 0.230 |
| STM2687 | *yfjG* | putative Oligoketide cyclase/lipid transport protein | 3.031 | 0.033 |
| STM4367 | *yjeB* | putative negative regulator | 3.028 | 0.029 |
| STM2395 | *pgtE* | Phosphoglycerate transport: outer membrane protein E | 3.026 | 0.073 |
| STM1569 | *fdnH* | formate dehydrogenase-N, Fe-S beta subunit, nitrate-inducible | 3.025 | 0.135 |
| PSLT035 | *PSLT035* | putative transposase | 3.022 | 0.024 |
| STM2613 | *STM2613* | Gifsy-1 prophage | 3.021 | 0.285 |
| STM2660 | *clpB* | ATP-dependent protease, Hsp 100, part of novel multi-chaperone system with DnaK, DnaJ, and GrpE | 3.012 | 0.048 |
| STM0807 | *ybhL* | putative permease | 3.010 | 0.021 |
| STM3132 | *STM3132* | putative xylanase/chitin deacetylase | 3.007 | 0.094 |
| STM3099 | *yggR* | putative protein transport | 3.007 | 0.173 |
| STM0430 | *phnR* | 2-aminoethylphosphonate transport, repressor | 3.005 | 0.193 |
| STM0109 | *yabN* | paral putative periplasmic binding protein of transport system | 3.004 | 0.092 |
| STM4207 | *STM4207* | putative phage baseplate component | 3.003 | 0.195 |
| STM1106 | *hpaI* | 4-hydroxyphenylacetate catabolism | 3.002 | 0.199 |
| STM1662 | *ynaJ* | putative inner membrane protein | 2.989 | 0.018 |
| STM0563 | *STM0563* | putative transcriptional regulator (AraC/XylS family) | 2.976 | 0.164 |
| STM0201 | *STM0201* | putative outer membrane protein | 2.976 | 0.115 |
| STM3900 | *ilvL* | ilvGEDA operon leader peptide | 2.968 | 0.113 |
| STM0437 | *STM0437* | putative periplasmic protein | 2.966 | 0.101 |
| STM1324 | *STM1324* | putative cytoplasmic protein | 2.961 | 0.065 |
| STM1309 | *STM1309* | putative nuclease subunit of the excinuclease complex | 2.959 | 0.110 |
| STM2373 | *STM2373* | putative cytoplasmic protein | 2.956 | 0.197 |
| STM1372 | *sufD* | required for stability of iron-sulfur component of FhuF | 2.942 | 0.071 |
| STM0146 | *ampD* | N-acetyl-anhydromuramyl-L-alanine amidase | 2.941 | 0.107 |
| STM0971 | *STM0971* | putative cytoplasmic protein | 2.938 | 0.279 |
| STM2070 | *yeeZ* | putative dehydratase | 2.937 | 0.027 |
| STM3594 | *prlC* | oligopeptidase A | 2.934 | 0.072 |
| STM0464 | *tesB* | acyl-CoA thioesterase II | 2.934 | 0.094 |
| STM3119 | *STM3119* | putative monoamine oxidase | 2.925 | 0.096 |
| STM3710 | *rfaD* | ADP-L-glycero-D-mannoheptose-6-epimerase | 2.919 | 0.030 |
| STM2478 | *STM2478* | putative beta-lactamase class C | 2.909 | 0.046 |
| STM1762 | *narJ* | nitrate reductase 1, delta subunit, chaperone required for molybdenum cofactor assembly in nitrate reductase 1 | 2.908 | 0.237 |
| STM0156 | *STM0156* | putative periplasmic protein | 2.906 | 0.328 |
| STM1960 | *fliD* | flagellar biosynthesis; filament capping protein; enables filament assembly | 2.905 | 0.115 |
| STM4088 | *yiiU* | putative cytoplasmic protein | 2.902 | 0.054 |
| STM4034 | *fdhE* | putative formate dehydrogenase formation protein ? Mn_fn | 2.901 | 0.058 |
| STM0411 | *yajD* | putative cytoplasmic protein | 2.897 | 0.025 |
| STM1620 | *STM1620* | putative oxidase | 2.896 | 0.166 |
| STM2275 | *STM2275* | putative regulatory protein, gntR family | 2.886 | 0.196 |
| STM1993 | *yedJ* | putative hydrolase | 2.880 | 0.117 |
| STM0256 | *yafC* | putative transcriptional regulator, LysR family | 2.878 | 0.141 |
| STM0261 | *gloB* | hydroxyacylglutathione hydrolase | 2.871 | 0.056 |
| STM1102 | *hpaE* | 4-hydroxyphenylacetate catabolism | 2.865 | 0.133 |
| STM3262 | *STM3262* | transcriptional regulator of sugar metabolism | 2.859 | 0.118 |
| PSLT005 | *tap* | replication of plasmid | 2.857 | 0.145 |
| PSLT047 | *PSLT047* | putative cytoplasmic protein | 2.848 | 0.044 |
| STM0780 | *STM0780* | putative outer membrane or exported | 2.844 | 0.184 |
| STM0327 | *STM0327* | putative cytoplasmic protein | 2.840 | 0.091 |
| STM2175 | *STM2175* | putative monooxygenase | 2.838 | 0.062 |
| STM3622 | *yhjS* | putative cytoplasmic protein | 2.829 | 0.056 |
| STM3136 | *STM3136* | putative D-mannonate oxidoreductase | 2.825 | 0.129 |
| PSLT099 | *trbB* | conjugative transfer | 2.813 | 0.117 |
| STM1802 | *dadX* | alanine racemase 2, catabolic | 2.797 | 0.113 |
| STM2640 | *rpoE* | sigma E (sigma 24 ) factor of RNA polymerase, response to periplasmic stress | 2.794 | 0.044 |
| STM3404 | *smg* | putative cytoplasmic protein | 2.794 | 0.030 |
| STM1112 | *cbpA* | curved DNA-binding protein | 2.788 | 0.043 |
| STM2190 | *mglB* | ABC superfamily (peri_perm), galactose transport protein | 2.787 | 0.072 |
| STM0272 | *STM0272* | putative ATPase with chaperone activity; homologue of Yersinia clpB | 2.784 | 0.084 |
| STM3122 | *STM3122* | putative arylsulfatase | 2.783 | 0.071 |
| STM3888 | *yieP* | putative regulatory protein, gntR family | 2.781 | 0.032 |
| STM0585 | *fepA* | outer membrane porin, receptor for ferric enterobactin (enterochelin) and colicins B and D | 2.778 | 0.051 |
| STM4007 | *glnA* | glutamine synthetase | 2.774 | 0.123 |
| STM4587 | *creA* | putative periplasmic protein | 2.772 | 0.033 |
| STM0575 | *STM0575* | putative inner membrane protein | 2.768 | 0.142 |
| STM0195 | *stfA* | putative fimbrial subunit | 2.762 | 0.143 |
| STM4542 | *yjjA* | putative outer membrane protein | 2.759 | 0.127 |
| STM3176 | *ygiW* | putative outer membrane protein | 2.739 | 0.030 |
| STM2851 | *hycC* | hydrogenase 3, membrane subunit (part of FHL complex) | 2.720 | 0.172 |
| PSLT042 | *PSLT042* | putative integrase protein | 2.716 | 0.050 |
| STM2809 | *proV* | ABC superfamily (atp_bind), glycine/betaine/proline transport protein | 2.715 | 0.168 |
| STM4066 | *STM4066* | putative sugar kinase, ribokinase family | 2.714 | 0.078 |
| STM1590 | *yncA* | putative acyltransferase | 2.701 | 0.040 |
| STM4380 | *yjfP* | putative hydrolase of the alpha/beta superfamily | 2.693 | 0.082 |
| STM0857 | *STM0857* | putative acyl-CoA dehydrogenase | 2.682 | 0.135 |
| STM1143 | *csgB* | minor curlin subunit precursor, nucleator for assembly of adhesive surface organelles | 2.675 | 0.166 |
| STM0262 | *yafS* | putative SAM-dependent methyltransferase | 2.666 | 0.045 |
| STM2358 | *STM2358* | putative cytoplasmic protein | 2.666 | 0.128 |
| STM3753 | *sugR* | ATP binding protein | 2.659 | 0.017 |
| STM3458 | *yheR* | putative NAD(P)H oxidoreductase | 2.641 | 0.041 |
| STM0776 | *galE* | UDP-galactose 4-epimerase | 2.641 | 0.087 |
| STM3625 | *yhjV* | putative HAAAP family transport protein | 2.627 | 0.067 |
| STM1181 | *flgI* | putative flagella basal body protein | 2.574 | 0.201 |
| STM2799 | *stpA* | DNA-binding protein with chaperone activity | 2.562 | 0.035 |
| STM1470 | *tus* | replication termination protein | 2.559 | 0.085 |
| STM0802 | *moaA* | molybdopterin biosynthesis, protein A | 2.557 | 0.089 |
| STM1697 | *STM1697* | putative Diguanylate cyclase/phosphodiesterase domain 2 | 2.552 | 0.117 |
| PSLT048 | *tlpA* | alpha-helical coiled coil protein | 2.550 | 0.024 |
| STM3144 | *hybF* | putative hydrogenase expression/formation protein | 2.533 | 0.064 |
| STM2955 | *STM2955* | putative transcriptional regulators containing the CopG/Arc/MetJ DNA-binding domain and a metal-binding domain | 2.532 | 0.051 |
| STM2359 | *STM2359* | putative amino acid transporter | 2.532 | 0.085 |
| PSLT015 | *orf5* | putative outer membrane protein | 2.522 | 0.112 |
| STM3587 | *yhiI* | paral putative membrane protein | 2.521 | 0.159 |
| STM0941 | *ybjY* | paral putative membrane protein | 2.513 | 0.060 |
| STM4415 | *fbp* | fructose-bisphosphatase | 2.513 | 0.019 |
| STM4199 | *STM4199* | putative cytoplasmic protein | 2.505 | 0.107 |
| STM3637 | *lpfD* | long polar fimbrial operon protein | 2.504 | 0.157 |
| STM3031 | *STM3031* | ail and ompX homologue | 2.498 | 0.039 |
| STM1710 | *pgpB* | phosphatidylglycerophosphate phosphatase B | 2.491 | 0.061 |
| STM4532 | *yjiY* | putative carbon starvation protein | 2.471 | 0.071 |
| STM2036 | *pocR* | Propanediol utilization: transcriptional regulation, AraC family | 2.468 | 0.093 |
| PSLT103 | *traT* | conjugative transfer: surface exclusion | 2.457 | 0.052 |
| STM3131 | *STM3131* | putative cytoplasmic protein | 2.431 | 0.139 |
| STM1158 | *STM1158* | putative inner membrane protein | 2.425 | 0.064 |
| STM3280 | *deaD* | cysteine sulfinate desulfinase | 2.419 | 0.103 |
| STM3511 | *yhgI* | putative Thioredoxin-like proteins and domain | 2.414 | 0.025 |
| STM1642 | *acpD* | acyl carrier protein phosphodiesterase | 2.411 | 0.127 |
| STM0421 | *yajO* | putative oxidoreductase / K + channel protein | 2.394 | 0.059 |
| STM1524 | *yneI* | putative succinate-semialdehyde dehydrogenase | 2.383 | 0.069 |
| STM1079 | *yccV* | putative inner membrane protein | 2.382 | 0.044 |
| STM1157 | *yceI* | putative secreted protein | 2.360 | 0.050 |
| STM1610 | *ydcK* | putative nucleoside-diphosphate-sugar pyrophosphorylases involved in lipopolysaccharide biosynthesis | 2.359 | 0.046 |
| STM0787 | *hutI* | Imidazolonepropionase | 2.352 | 0.097 |
| STM0803 | *moaB* | molybdopterin biosynthesis, protein B | 2.344 | 0.065 |
| STM4033 | *STM4033* | putative bacterial regulatory protein, merR family | 2.340 | 0.065 |
| STM0353 | *STM0353* | putative cation transport ATPase | 2.338 | 0.089 |
| STM3881 | *rbsD* | D-ribose high-affinity transport system; membrane-associated protein | 2.321 | 0.130 |
| STM4037 | *fdoG* | formate dehydrogenase | 2.318 | 0.053 |
| STM4077 | *yneA* | putative ABC superfamily (peri_perm), sugar transport protein | 2.317 | 0.152 |
| STM4189 | *yjbB* | putative PNaS family transport protein | 2.307 | 0.104 |
| STM3969 | *yigN* | putative inner membrane protein | 2.279 | 0.062 |
| STM3907 | *STM3907* | putative cytoplasmic protein | 2.277 | 0.128 |
| STM2069 | *yeeY* | putative transcriptional regulator, LysR family | 2.271 | 0.064 |
| STM1348 | *ydiA* | putative inner membrane protein | 2.257 | 0.032 |
| STM1154 | *yceE* | putative MFS family transport protein | 2.254 | 0.051 |
| STM0267 | *STM0267* | putative cytoplasmic protein | 2.251 | 0.100 |
| STM3568 | *rpoH* | sigma H (sigma 32) factor of RNA polymerase; transcription of heat shock proteins induced by cytoplasmic stress | 2.182 | 0.030 |
| STM0481 | *priC* | primosomal replication protein N | 2.147 | 0.072 |
| **Genes downregulated** | | | | |
| STM3597 | *gor* | glutathione oxidoreductase | -2.030 | 0.510 |
| STM0242 | *proS* | proline tRNA synthetase | -2.001 | 0.285 |
| STM2512 | *xseA* | exonuclease VII, large subunit | -2.004 | 0.620 |
| STM3449 | *yheL* | putative oxidation of intracellular sulfur | -2.006 | 0.352 |
| STM2312 | *elaA* | putative acyltransferase | -2.001 | 0.298 |
| STM0392 | *rdgC* | putative exonuclease involved in removal of stalled replication fork | -2.030 | 1.969 |
| STM2779 | *aSTM2779* | putative inner membrane protein | -2.019 | 0.482 |
| STM3109 | *yggH* | putative S-adenosylmethionine-dependent methyltransferase | -2.019 | 0.186 |
| STM2365 | *folC* | multifunctional folylpolyglutamate synthase; dihydrofolate synthase, also has formylTHF polyglutamate synthase activity | -2.031 | 0.396 |
| STM1123 | *STM1123* | putative periplasmic protein | -2.031 | 0.671 |
| STM1761 | *narI* | nitrate reductase 1, cytochrome b(NR), gamma subunit | -2.008 | 1.286 |
| STM1740 | *yciU* | putative cytoplasmic protein | -2.012 | 0.498 |
| STM1192 | *plsX* | putative fatty acid/phospholipid synthesis protein | -2.034 | 0.542 |
| STM2778 | *STM2778* | pseudogene; frameshift | -2.046 | 0.646 |
| STM3837 | *dnaN* | DNA polymerase III, beta-subunit | -2.047 | 0.365 |
| STM4029 | *yiiD* | putative acetyltransferase | -2.048 | 0.521 |
| STM3972 | *aarF* | putative regulator in ubiquinone biosynthesis | -2.050 | 0.420 |
| STM1282 | *yeaK* | putative cytoplasmic protein | -2.056 | 0.436 |
| STM0698 | *pgm* | phosphoglucomutase | -2.060 | 0.405 |
| STM0049 | *lytB* | regulates the activity of guanosine 3',5'-bispyrophosphate synthetase I (RelA) | -2.065 | 0.432 |
| STM1459 | *STM1459* | putative oxidoreductase, inner membrane protein | -2.066 | 1.339 |
| PSLT053 | *parB* | plasmid partition protein B | -2.067 | 0.224 |
| STM0589 | *fepE* | ferric enterobactin (enterochelin) transporter | -2.068 | 0.586 |
| STM2366 | *accD* | acetylCoA carboxylase, beta subunit | -2.072 | 0.398 |
| STM4027 | *rbn* | tRNA processing exoribonuclease BN | -2.080 | 0.437 |
| STM3013 | *lysA* | diaminopimelate decarboxylase | -2.081 | 0.831 |
| STM0960 | *ftsK* | cell division protein, required for cell division and chromosome partitioning | -2.086 | 0.538 |
| STM1540 | *STM1540* | putative hydrolase | -2.094 | 0.601 |
| STM0796 | *bioC* | biotin biosynthesis; reaction prior to pimeloyl CoA | -2.097 | 1.368 |
| STM0307 | *STM0307* | homology to Shigella VirG protein | -2.097 | 0.754 |
| STM3496 | *yrfG* | putative hydrolase | -2.098 | 0.342 |
| STM0398 | *phoR* | sensory kinase in two-component regulatory system with PhoB, regulates pho regulon | -2.098 | 0.403 |
| STM0747 | *tolA* | tol protein, membrane spanning protein | -2.099 | 0.671 |
| STM1639 | *cybB* | cytochrome b(561) | -2.114 | 0.823 |
| STM1185 | *rne* | RNase E | -2.118 | 0.761 |
| STM4148 | *nusG* | component in transcription antitermination | -2.120 | 0.856 |
| STM1714 | *topA* | DNA topoisomerase type I, omega protein | -2.125 | 0.309 |
| STM3947 | *dapF* | diaminopimelate epimerase | -2.125 | 0.305 |
| STM0092 | *surA* | peptidyl-prolyl cis-trans isomerase, survival protein | -2.126 | 0.493 |
| STM3584 | *nikR* | nickel-responsive transcriptional regulator | -2.129 | 1.066 |
| STM3921 | *wecC* | UDP-N-acetyl-D-mannosaminuronic acid dehydrogenase | -2.129 | 0.337 |
| STM3346 | *yhcM* | putative ATPase | -2.130 | 0.965 |
| STM3140 | *yghU* | putative glutathione S-transferase | -2.142 | 0.150 |
| STM1902 | *yecD* | putative isochorismatase | -2.143 | 0.538 |
| STM2096 | *rfbD* | TDP-rhamnose synthetase | -2.155 | 0.278 |
| STM2543 | *nifS* | putative aminotransferase class-V | -2.156 | 0.582 |
| STM0713 | *ybgK* | putative carboxylase | -2.175 | 0.415 |
| STM0778 | *modF* | putative ABC superfamily (atp_bind), molybdenum transporter | -2.182 | 0.773 |
| STM3997 | *dsbA* | periplasmic protein disulfide isomerase I | -2.189 | 0.773 |
| STM0962 | *ycaJ* | paral putative polynucleotide enzyme | -2.195 | 0.653 |
| STM0150 | *aroP* | APC family, aromatic amino acid transporter | -2.198 | 0.558 |
| STM3067 | *yggB* | putative membrane protein, involved in stability of MscS mechanosensitive channel | -2.206 | 0.536 |
| STM3922 | *rffG* | dTDP-glucose 4,6-dehydratase | -2.220 | 1.390 |
| STM1890 | *yebA* | putative Peptidase | -2.220 | 0.658 |
| STM1717 | *yciK* | putative oxoacyl-(acyl carrier protein) reductase | -2.232 | 0.495 |
| STM3086 | *speA* | arginine decarboxylase | -2.232 | 0.570 |
| STM0737 | *sucB* | 2-oxoglutarate dehydrogenase (dihydrolipoyltranssuccinase E2 component) | -2.239 | 0.796 |
| STM0221 | *uppS* | undecaprenyl pyrophosphate synthetase (di-trans,poly-cis-decaprenylcistransferase) | -2.240 | 0.617 |
| STM1186 | *STM1186* | pseudogene; in-frame stop following codon 97; no start near coli start | -2.242 | 0.468 |
| STM1226 | *potA* | ABC superfamily (atp_bind), spermidine/putrescine transporter | -2.243 | 1.101 |
| STM2122 | *udk* | uridine/cytidine kinase | -2.245 | 0.611 |
| STM0733 | *sdhD* | succinate dehydrogenase, hydrophobic subunit | -2.252 | 0.957 |
| STM2663 | *yfiO* | putative lipoprotein | -2.258 | 0.634 |
| STM0128 | *murG* | UDP-N-acetylglucosamine:N-acetylmuramyl-(pentapeptide) pyrophosphoryl-undecaprenol N-acetylglucosamine transferase | -2.259 | 0.431 |
| PSLT038 | *spvC* | Salmonella plasmid virulence: hydrophilic protein | -2.267 | 2.070 |
| STM3501 | *envZ* | sensory histidine kinase in two-component regulatory system with OmpR | -2.276 | 0.344 |
| STM3318 | *yhbN* | putative ABC superfamily (bind_prot) transport protein | -2.276 | 0.236 |
| STM2098 | *galF* | putative glucose-1-phosphate uridylyltransferase (UDP-glucose pyrophosphorylase), non-catalytic subunit | -2.277 | 0.358 |
| STM3718 | *rfaI* | UDP-D-galactose:(glucosyl)lipopolysaccharide-alpha-1,3-D-galactosyltransferase | -2.280 | 0.448 |
| STM1700 | *fabI* | enoyl-[acyl-carrier-protein] reductase (NADH) | -2.281 | 0.194 |
| STM0487 | *htpG* | chaperone Hsp90, heat shock protein C 62.5 | -2.287 | 0.908 |
| STM1347 | *aroH* | 3-deoxy-D-arabinoheptulosonate-7-phosphate synthase (DAHP synthetase), tryptophan repressible | -2.299 | 1.901 |
| STM1291 | *yeaA* | putative domain frequently associated with peptide methionine sulfoxide reductase | -2.301 | 0.687 |
| STM1746 | *oppA* | ABC superfamily (periplasm), oligopeptide transport protein with chaperone properties | -2.310 | 0.389 |
| STM0122 | *ftsI* | division specific transpeptidase, penicillin-binding protein 3 re | -2.311 | 0.526 |
| STM0933 | *ybjT* | putative nucleoside-diphosphate-sugar epimerase | -2.318 | 0.393 |
| STM1734 | *yciC* | putative inner membrane protein | -2.318 | 0.534 |
| STM1534 | *STM1534* | putative hydrogenase | -2.328 | 2.429 |
| STM4121 | *argC* | N-acetyl-gamma-glutamylphosphate reductase | -2.331 | 0.741 |
| STM0795 | *bioF* | 7-keto-8-aminopelargonic acid synthetase | -2.336 | 0.890 |
| STM0316 | *pepD* | aminoacyl-histidine dipeptidase (peptidase D) | -2.338 | 0.719 |
| STM1450 | *pdxY* | pyridoxal kinase 2/pyridoxine kinase | -2.340 | 0.415 |
| STM2929 | *ispF* | 2C-methyl-d-erythritol-2,4-cyclodiphosphate synthase | -2.341 | 2.513 |
| STM3917 | *rho* | transcription termination factor Rho; polarity suppressor | -2.341 | 0.800 |
| STM2370 | *pdxB* | erythronate-4-phosphate dehyrogenase | -2.341 | 0.640 |
| STM0322 | *proA* | gamma-glutamylphosphate reductase | -2.344 | 2.401 |
| STM1479 | *pntA* | pyridine nucleotide transhydrogenase (proton pump), alpha subunit | -2.350 | 0.587 |
| STM3296 | *hflB* | ATP-dependent zinc-metallo protease | -2.354 | 0.783 |
| STM0090 | *ksgA* | S-adenosylmethionine-6-N',N'-adenosyl (rRNA) dimethyltransferase; kasugamycin resistance | -2.355 | 0.577 |
| STM3877 | *asnA* | asparagine synthetase A | -2.356 | 0.804 |
| STM4175 | *purD* | phosphoribosylglycinamide synthetase (GAR synthetase) | -2.359 | 0.877 |
| STM1400 | *sseC* | Secretion system effector | -2.361 | 0.156 |
| STM1078 | *STM1078* | putative cytoplasmic protein | -2.363 | 0.256 |
| STM1599 | *pdgL* | Periplasmic dipeptidase for D-ala-D-ala digestion in peptidoglycan | -2.363 | 0.355 |
| STM0093 | *imp* | Organic solvent tolerance protein | -2.376 | 0.677 |
| STM3483 | *rpe* | D-ribulose-5-phosphate 3-epimerase | -2.378 | 0.523 |
| STM1830 | *manX* | Sugar Specific PTS family, mannose-specific enzyme IIAB | -2.386 | 0.800 |
| STM3482 | *gph* | phosphoglycolate phosphatase | -2.391 | 0.429 |
| STM0837 | *ybiS* | putative periplasmic protein | -2.402 | 0.611 |
| STM0132 | *ftsA* | ATP-binding cell division protein, septation process, complexes with FtsZ, associated with junctions of inner and outer membranes | -2.405 | 0.194 |
| STM0260 | *dniR* | transcriptional regulator for nitrite reductase (cytochrome c552) | -2.407 | 0.468 |
| STM1266 | *STM1266* | putative transcriptional regulator | -2.409 | 1.063 |
| STM0972 | *STM0972* | homologous to secreted protein sopD | -2.420 | 0.273 |
| STM1638 | *STM1638* | putative SAM-dependent methyltransferases | -2.421 | 2.218 |
| STM4026 | *yihX* | paral putative enzyme | -2.423 | 0.582 |
| STM3979 | *ubiB* | FMN reductase | -2.424 | 0.738 |
| STM3854 | *pstB* | ABC superfamily (atp_bind), high-affinity phosphate transporter | -2.434 | 1.203 |
| STM2081 | *gnd* | gluconate-6-phosphate dehydrogenase, decarboxylating | -2.434 | 0.707 |
| STM2226 | *yejK* | nucleotide associated protein, present in spermidine nucleoids | -2.434 | 0.304 |
| STM1537 | *STM1537* | putative Ni/Fe-hydrogenase 1 b-type cytochrome subunit | -2.437 | 0.971 |
| STM1737 | *tonB* | energy transducer; uptake of iron, cyanocobalimin; sensitivity to phages, colicins | -2.442 | 0.749 |
| STM0441 | *cyoC* | cytochrome o ubiquinol oxidase subunit III | -2.450 | 0.651 |
| STM3370 | *cafA* | RNase G | -2.454 | 0.633 |
| STM1153 | *msyB* | acidic protein suppresses mutants lacking function of protein export | -2.460 | 0.825 |
| STM4364 | *hflC* | with HflK, part of modulator for protease specific for FtsH phage lambda cII repressor | -2.463 | 0.477 |
| STM0452 | *cypD* | peptidyl prolyl isomerase | -2.467 | 0.458 |
| STM0153 | *aceF* | pyruvate dehydrogenase, dihydrolipoyltransacetylase component | -2.472 | 0.826 |
| STM1539 | *STM1539* | putative hydrogenase-1 small subunit | -2.476 | 0.894 |
| STM1207 | *ycfM* | putative outer membrane lipoprotein | -2.476 | 0.688 |
| STM3286 | *infB* | protein chain initiation factor IF-2 | -2.476 | 0.588 |
| STM3874 | *gidA* | associate with glucose-inhibited division | -2.479 | 0.560 |
| STM1135 | *ycdW* | paral putative oxidoreductase | -2.498 | 1.010 |
| STM3730 | *dfp* | flavoprotein affecting synthesis of DNA and pantothenate metabolism | -2.498 | 0.202 |
| STM1651 | *nifJ* | putative pyruvate-flavodoxin oxidoreductase | -2.501 | 0.434 |
| STM0443 | *cyoA* | cytochrome o ubiquinol oxidase subunit II | -2.504 | 0.830 |
| STM2583 | *lepA* | GTP-binding elongation factor | -2.505 | 0.389 |
| STM2061 | *sbmC* | DNA gyrase inhibitor | -2.509 | 1.466 |
| STM0383 | *iraP* |  | -2.515 | 0.230 |
| STM3323 | *yhbJ* | putative P-loop-containing kinase | -2.516 | 0.962 |
| STM1391 | *ssrB* | Secretion system regulator: transcriptonal activator, homologous with degU/uvrY/bvgA | -2.520 | 0.312 |
| STM0211 | *yaeH* | putative cytoplasmic protein | -2.531 | 0.726 |
| STM0440 | *cyoD* | cytochrome o ubiquinol oxidase subunit IV | -2.532 | 0.719 |
| STM2542 | *nifU* | NifU homologs involved in Fe-S cluster formation | -2.536 | 0.903 |
| STM2141 | *fbaB* | 3-oxoacyl-[acyl-carrier-protein] synthase I | -2.539 | 1.297 |
| STM1796 | *treA* | trehalase, periplasmic | -2.550 | 0.475 |
| STM2536 | *pepB* | putative aminopeptidase | -2.560 | 0.393 |
| STM1448 | *pdxH* | pyridoxine 5'-phosphate oxidase | -2.561 | 0.665 |
| STM3320 | *rpoN* | sigma N (sigma 54) factor of RNA polymerase, pleiotrophic functions (nitrogen metabolism, formate degradation, phage shock response) | -2.569 | 0.481 |
| STM1334 | *infC* | protein chain initiation factor IF-3 | -2.575 | 0.928 |
| STM0736 | *sucA* | 2-oxoglutarate dehydrogenase (decarboxylase component) | -2.576 | 0.892 |
| STM0213 | *dapD* | 2,3,4,5-tetrahydropyridine-2-carboxylate N-succinyltransferase | -2.577 | 0.944 |
| STM0439 | *cyoE* | protohaeme IX farnesyltransferase (haeme O biosynthesis) | -2.586 | 0.497 |
| STM2074 | *hisB* | bifunctional: imidazoleglycerol-phosphate dehydratase; histidinol-phosphatase | -2.590 | 1.352 |
| STM1195 | *fabG* | 3-oxoacyl-[acyl-carrier-protein] reductase | -2.600 | 0.682 |
| STM3282 | *pnp* | polynucleotide phosphorylase, member of mRNA degradosome | -2.602 | 0.834 |
| STM2347 | *yfcE* | putative phosphoesterase | -2.606 | 0.765 |
| STM3872 | *atpI* | membrane-bound ATP synthase subunit, F1-F0-type proton-ATPase | -2.614 | 0.492 |
| STM0738 | *sucC* | succinyl-CoA synthetase, beta subunit | -2.618 | 0.876 |
| STM3076 | *tktA* | transketolase 1 isozyme | -2.624 | 0.570 |
| STM2415 | *gltX* | glutamate tRNA synthetase, catalytic subunit | -2.628 | 0.483 |
| STM0125 | *mraY* | phospho-N-acetylmuramoyl-pentapeptide transferase | -2.632 | 0.651 |
| STM0694 | *fldA* | flavodoxin 1 | -2.642 | 0.797 |
| STM3715 | *rfaZ* | lipopolysaccharide core biosynthesis | -2.642 | 0.509 |
| STM2091 | *rfbG* | LPS side chain defect: CDP glucose 4,6-dehydratase | -2.646 | 0.720 |
| STM1935 | *ftn* | cytoplasmic ferritin | -2.647 | 2.757 |
| STM1756 | *purU* | formyltetrahydrofolate hydrolase | -2.649 | 0.564 |
| STM0667 | *ybeX* | putative CBS domain-containing protein | -2.654 | 0.988 |
| STM4293 | *yjdB* | putative integral membrane protein | -2.678 | 0.935 |
| STM2652 | *pssA* | phosphatidylserine synthase (CDP-diacylglycerol-serine O-phosphatidyltransferase) | -2.679 | 0.908 |
| STM2137 | *STM2137* | putative cytoplasmic protein | -2.685 | 0.782 |
| STM0748 | *tolB* | tol protein required for outer membrane integrity, uptake of group A colicins, and translocation of phage DNA to cytoplasm, may be part of multiprotein peptidoglycan recycling complex (Two domains) | -2.690 | 0.509 |
| STM0136 | *secA* | preprotein translocase; secretion protein of IISP family | -2.691 | 0.937 |
| STM0126 | *murD* | UDP-N-acetylmuramoylalanine-D-glutamate ligase | -2.701 | 0.709 |
| STM0641 | *ybeA* | putative cytoplasmic protein | -2.721 | 1.177 |
| STM0320 | *phoE* | outer membrane pore protein e (e,ic,nmpab) | -2.725 | 0.684 |
| STM0974 | *focA* | putative FNT family, formate transporter (formate channel 1) | -2.726 | 0.836 |
| STM3724 | *kdtA* | 3-deoxy-D-manno-octulosonic-acid transferase (KDO transferase) | -2.727 | 1.084 |
| STM0436 | *yajR* | putative MFS family transporter | -2.737 | 0.687 |
| STM1422 | *ssaU* | Secretion system apparatus: homology with YscU of the secretion system of Yersinia | -2.749 | 2.576 |
| STM2511 | *guaB* | IMP dehydrogenase | -2.749 | 0.572 |
| STM4494 | *STM4494* | putative ABC-type sugar/spermidine/putrescine transport systems, ATPase component | -2.752 | 0.641 |
| STM0108 | *tbpA* | thiamine-binding periplasmic protein | -2.759 | 0.822 |
| STM1193 | *fabH* | 3-oxoacyl-[acyl-carrier-protein] synthase III; acetylCoA ACP transacylase | -2.763 | 0.453 |
| STM2346 | *STM2346* | putative NTP pyrophosphohydrolase | -2.786 | 0.563 |
| STM3857 | *pstS* | ABC superfamily (bind_prot), high-affinity phosphate transporter | -2.788 | 0.333 |
| STM3101 | *yggT* | putative integral membran resistance protein | -2.794 | 0.613 |
| STM0711 | *ybgI* | putative cytoplasmic protein | -2.810 | 0.673 |
| STM4092 | *hslV* | peptidase component of the HslUV protease | -2.811 | 0.710 |
| STM0647 | *rlpB* | a minor lipoprotein | -2.815 | 1.083 |
| STM3645 | *yiaD* | putative outer membrane lipoprotein | -2.819 | 0.917 |
| STM1197 | *fabF* | 3-oxoacyl-[acyl-carrier-protein] synthase II | -2.830 | 0.852 |
| STM0794 | *bioB* | biotin synthetase | -2.833 | 0.318 |
| STM3401 | *aroE* | dehydroshikimate reductase | -2.833 | 1.089 |
| STM1408 | *ssaI* | Secretion system apparatus | -2.839 | 1.279 |
| STM0223 | *yaeL* | putative membrane-associated Zn-dependent protease | -2.840 | 0.898 |
| STM2213 | *yeiU* | putative permease | -2.841 | 0.609 |
| STM0605 | *ybdN* | putative 3'-phosphoadenosine 5'-phosphosulfate sulfotransferase (PAPS reductase)/FAD synthetase | -2.841 | 0.796 |
| STM4091 | *hslU* | ATPase component of the HslUV protease | -2.847 | 0.816 |
| STM1410 | *STM1410* | putative cytoplasmic protein | -2.859 | 0.250 |
| STM2488 | *nlpB* | lipoprotein-34 | -2.887 | 1.000 |
| STM3731 | *dut* | deoxyuridinetriphosphatase | -2.887 | 0.647 |
| STM3374 | *mreB* | rod shape-determining protein; HSP70 class molecular chaperones involved in cell morphogenesis | -2.890 | 0.827 |
| STM0760 | *aroG* | 3-deoxy-D-arabinoheptulosonate-7-phosphate synthase (DAHP synthetase, phenylalanine repressible) | -2.893 | 1.048 |
| STM0670 | *miaB* | methylthiolation of isopentenylated A37 derivatives in rRNA | -2.913 | 0.430 |
| STM0940 | *ybjX* | homologue of virK | -2.915 | 0.806 |
| STM3840 | *rnpA* | RNase P, protein component (protein C5), processes tRNA, 4.5S RNA | -2.919 | 1.418 |
| STM3385 | *fis* | site-specific DNA inversion stimulation factor | -2.935 | 1.979 |
| STM4541 | *mdoB* | phosphoglycerol transferase I | -2.936 | 1.293 |
| STM1127 | *STM1127* | putative transcriptional regulator | -2.937 | 0.566 |
| STM2138 | *STM2138* | putative cytoplasmic protein | -2.958 | 0.806 |
| STM1544 | *pqaA* | PhoPQ-regulated protein | -2.972 | 0.697 |
| STM0084 | *STM0084* | putative sulfatase | -2.986 | 0.959 |
| STM4495 | *STM4495* | putative type II restriction enzyme, methylase subunit | -2.987 | 0.610 |
| STM0124 | *murF* | D-alanine:D-alanine-adding enzyme | -2.990 | 0.896 |
| STM4362 | *hflX* | putative GTP-ase, together with HflCK possibly involved in phage lambda cII repressor stability | -3.005 | 0.942 |
| STM1427 | *cfa* | cyclopropane fatty acyl phospholipid synthase | -3.005 | 0.886 |
| STM4226 | *yjbA* | putative inner membrane protein | -3.007 | 0.636 |
| STM0226 | *lpxD* | UDP-3-O-(3-hydroxymyristoyl)-glucosamine n-acyltransferase | -3.012 | 0.777 |
| STM3723 | *rfaQ* | lipopolysaccharide core biosynthesis; modification of heptose region of core | -3.012 | 0.629 |
| STM0152 | *aceE* | pyruvate dehydrogenase, decarboxylase component | -3.014 | 0.944 |
| STM0357 | *mod* | DNA methylase; restriction system | -3.015 | 0.837 |
| STM0442 | *cyoB* | cytochrome o ubiquinol oxidase subunit I | -3.018 | 1.045 |
| STM2440 | *cysM* | cysteine synthase B (O-acetylserine sulfhydrolase B) | -3.031 | 1.261 |
| STM1773 | *ychA* | putative transcriptional regulator | -3.043 | 1.288 |
| STM4192 | *STM4192* | putative cytoplasmic protein | -3.046 | 0.865 |
| STM4248 | *tyrB* | tyrosine aminotransferase, tyrosine repressible | -3.051 | 1.262 |
| STM2385 | *yfcB* | putative methylase | -3.068 | 1.356 |
| STM2767 | *STM2767* | putative Superfamily I DNA and RNA helicase | -3.079 | 1.119 |
| STM3852 | *yieH* | putative phosphatase/phosphohexomutase | -3.084 | 1.614 |
| STM4475 | *valS* | valine tRNA synthetase | -3.086 | 0.838 |
| STM3498 | *yrfI* | heat shock protein 33, redox regulated chaparone | -3.086 | 0.427 |
| STM2369 | *usg* | putative aspartate-semialdehyde dehydrogenase | -3.089 | 1.186 |
| STM0123 | *murE* | UDP-N-acetylmuramoylalanyl-D-glutamate 2,6-diaminopimelate ligase | -3.095 | 0.877 |
| STM4348 | *psd* | phosphatidylserine decarboxylase | -3.107 | 0.921 |
| STM0007 | *talB* | transaldolase B | -3.109 | 0.725 |
| STM0997 | *ycbL* | putative Metallo-beta-lactamase | -3.110 | 0.721 |
| STM4157 | *STM4157* | putative cytoplasmic protein | -3.116 | 0.906 |
| STM3402 | *yrdC* | putative translation factor | -3.117 | 1.008 |
| STM2092 | *rfbF* | LPS side chain defect: glucose-1-phosphate cytidylyltransferase | -3.124 | 0.896 |
| STM3161 | *metC* | cystathionine beta-lyase (beta-cystathionase) | -3.126 | 0.530 |
| STM3537 | *glgX* | glycosyl hydrolase | -3.130 | 0.909 |
| STM4581 | *yjjK* | putative ABC superfamily (atp_bind) transport protein | -3.143 | 0.275 |
| STM1527 | *STM1527* | putative inner membrane protein | -3.146 | 0.941 |
| STM2090 | *rfbH* | LPS side chain defect: CDP-6deoxy-D-xylo-4-hexulose-3-dehydrase | -3.154 | 0.919 |
| STM4082 | *yiiQ* | putative periplasmic protein | -3.156 | 2.092 |
| STM2450 | *amiA* | N-acetylmuramoyl-l-alanine amidase I | -3.172 | 0.962 |
| STM0319 | *crl* | transcriptional regulator of cryptic csgA gene for curli surface fibers | -3.172 | 1.114 |
| STM2087 | *rfbV* | LPS side chain defect: abequosyltransferase | -3.173 | 2.275 |
| STM4123 | *argH* | argininosuccinate lyase | -3.173 | 0.710 |
| STM0993 | *mukE* | putative chromosome partitioning | -3.181 | 0.930 |
| STM2451 | *hemF* | coproporphyrinogen III oxidase | -3.197 | 0.618 |
| STM3231 | *yqjK* | putative inner membrane protein | -3.201 | 0.652 |
| STM1409 | *ssaJ* | Secretion system apparatus: homology with the yscJ/mxiJ/prgK family of lipoproteins | -3.210 | 0.456 |
| STM3742 | *spoT* | bifunctional : (p)ppGpp synthetase II; also guanosine-3',5'-bis pyrophosphate 3'-pyrophosphohydrolase | -3.214 | 1.014 |
| STM2992 | *argA* | N-alpha-acetylglutamate synthase (amino-acid acetyltransferase) | -3.223 | 0.923 |
| STM4152 | *rplL* | 50S ribosomal subunit protein L7/L12 | -3.233 | 1.017 |
| STM2489 | *dapA* | dihydrodipicolinate synthase | -3.235 | 1.114 |
| STM1699 | *ycjE* | putative cytoplasmic protein | -3.249 | 4.520 |
| STM1449 | *tyrS* | tyrosine tRNA synthetase | -3.252 | 0.443 |
| STM1393 | *ssaB* | Secretion system apparatus | -3.258 | 0.426 |
| STM4329 | *mopB* | chaperone Hsp10, affects cell division | -3.258 | 1.028 |
| STM2582 | *lepB* | leader peptidase (signal peptidase I), serine protease | -3.286 | 0.746 |
| STM4063 | *sbp* | ABC superfamily (bind_prot), sulfate transport protein | -3.287 | 0.792 |
| STM3905 | *ilvA* | threonine deaminase | -3.292 | 0.550 |
| STM0697 | *seqA* | negative modulator of initiation of replication, inhibits open complex formation, mutation in gene alters cell membrane | -3.305 | 1.239 |
| STM1004 | *pncB* | nicotinate phosphoribosyltransferase | -3.313 | 0.928 |
| STM0735 | *sdhB* | succinate dehydrogenase, Fe-S protein | -3.323 | 1.322 |
| STM0418 | *nusB* | transcription termination; L factor | -3.324 | 1.354 |
| STM1478 | *ydgH* | putative periplasmic protein | -3.328 | 0.603 |
| STM4477 | *pepA* | aminopeptidase A | -3.329 | 0.906 |
| STM1194 | *fabD* | malonyl-CoA-[acyl-carrier-protein] transacylase | -3.330 | 0.670 |
| STM3484 | *dam* | DNA adenine methylase | -3.346 | 0.594 |
| STM3321 | *yhbH* | putative sigma N modulation factor | -3.360 | 0.725 |
| STM4363 | *hflK* | with HflC, part of modulator for protease specific for FtsH phage lambda cII repressor | -3.366 | 0.978 |
| STM3964 | *metR* | regulator for metE and metH (LysR family) | -3.371 | 3.747 |
| STM1196 | *acpP* | acyl carrier protein | -3.372 | 0.946 |
| STM4549 | *STM4549* | putative cytoplasmic protein | -3.380 | 0.908 |
| STM2930 | *ispD* | 4-phosphocytidyl-2C-methyl-D-erythritol synthase | -3.381 | 1.207 |
| STM0628 | *pagP* | PhoPQ-activated gene | -3.401 | 1.210 |
| STM1845 | *prc* | carboxy-terminal protease for penicillin-binding protein 3 | -3.401 | 0.917 |
| STM2869 | *orgA* | putative flagellar biosynthesis/type III secretory pathway protein | -3.404 | 0.663 |
| STM3068 | *fba* | fructose-bisphosphate aldolase | -3.421 | 1.203 |
| STM3725 | *kdtB* | phosphopantetheine adenylyltransferase | -3.429 | 0.885 |
| STM3481 | *trpS* | tryptophan tRNA synthetase | -3.431 | 0.372 |
| STM1548 | *STM1548* | putative S-adenosylmethionine:tRNA-ribosyltransferase-isomerase | -3.441 | 0.449 |
| STM1318 | *katE* | catalase; hydroperoxidase HPII(III), RpoS dependent | -3.458 | 1.959 |
| STM1995 | *ompS1* | putative porin | -3.507 | 0.301 |
| STM3502 | *ompR* | response regulator in two-component regulatory system with EnvZ, affecting transcription of ompC and ompF (OmpR family) | -3.511 | 1.163 |
| STM4394 | *rplI* | 50S ribosomal subunit protein L9 | -3.535 | 0.661 |
| STM1270 | *yeaS* | paral putative transport protein | -3.535 | 1.417 |
| STM3523 | *glpR* | transcriptional repressor of the glp operon (DeoR family) | -3.563 | 1.102 |
| STM2584 | *gogB* | Gifsy-1 prophage: leucine-rich repeat protein | -3.570 | 1.338 |
| STM1836 | *STM1836* | putative penicillin-binding protein-3 | -3.577 | 1.173 |
| STM4493 | *STM4493* | putative cytoplasmic protein | -3.582 | 0.849 |
| STM0186 | *dksA* | dnaK suppressor protein | -3.595 | 1.140 |
| STM3567 | *livJ* | ABC superfamily (bind_prot), branched-chain amino acid transporter, high-affinity | -3.596 | 1.716 |
| STM1702 | *rnb* | RNase II, mRNA degradation | -3.598 | 0.924 |
| STM4366 | *purA* | adenylosuccinate synthetase | -3.599 | 1.372 |
| STM1150 | *mdoG* | periplasmic glucans biosynthesis protein | -3.612 | 0.993 |
| STM2079 | *wzzB* | regulator of length of O-antigen component of lipopolysaccharide chains | -3.623 | 0.887 |
| STM1396 | *ssaE* | Secretion system effector | -3.642 | 2.871 |
| STM3472 | *ppiA* | peptidyl-prolyl cis-trans isomerase A (rotamase A) | -3.659 | 1.037 |
| STM0166 | *speE* | spermidine synthase (putrescine aminopropyltransferase) | -3.681 | 0.721 |
| STM1294 | *ansA* | cytoplasmic L-asparaginase I | -3.686 | 1.350 |
| STM1701 | *yciW* | putative cytoplasmic protein | -3.695 | 4.955 |
| STM0745 | *tolQ* | tol protein, membrane-spanning inner membrane proteins, required for outer membrane integrity, uptake of group A colicins, and translocation of phage DNA to cytoplasm | -3.708 | 1.191 |
| STM3288 | *yhbC* | putative cytoplasmic protein | -3.709 | 1.569 |
| STM1538 | *STM1538* | putative hydrogenase-1 large subunit | -3.722 | 1.550 |
| STM1528 | *STM1528* | putative outer membrane protein | -3.725 | 1.164 |
| STM3041 | *prfB* | peptide chain release factor RF-2 | -3.740 | 1.014 |
| STM3322 | *ptsN* | sugar specific PTS family, enzyme IIA, also regulates N metabolism | -3.740 | 0.834 |
| STM3667 | *yiaJ* | transcriptional repressor (IclR family) | -3.754 | 0.602 |
| STM3655 | *glyS* | glycine tRNA synthetase, beta subunit | -3.757 | 1.112 |
| STM1846 | *proQ* | activator of proP | -3.762 | 1.384 |
| STM0417 | *ribH* | riboflavin synthase, beta chain | -3.776 | 1.129 |
| STM0218 | *pyrH* | uridylate kinase | -3.791 | 1.400 |
| STM3360 | *argR* | repressor of arg regulon (ArgR familiy) | -3.799 | 0.720 |
| STM4118 | *yijP* | putative Integral membrane protein | -3.800 | 1.295 |
| STM2331 | *yfbQ* | putative aminotransferase (ortho), paral putative regulator | -3.804 | 1.530 |
| STM1365 | *ydiJ* | paral putative oxidase | -3.809 | 0.710 |
| STM2641 | *nadB* | quinolinate synthetase, B protein | -3.811 | 0.715 |
| STM4334 | *efp* | elongation factor P (EF-P) | -3.825 | 1.506 |
| STM3898 | *yifE* | putative LysR type transcriptional regulator with pssR | -3.837 | 2.182 |
| STM2095 | *rfbA* | dTDP-glucose pyrophosphorylase | -3.860 | 1.707 |
| STM2538 | *fdx* | [2FE-2S] ferredoxin, electron carrer protein, believed to be involved in assembly of Fe-S clusters | -3.860 | 1.018 |
| STM0852 | *yliG* | putative Fe-S oxidoreductases family 1 | -3.861 | 1.214 |
| STM1899 | *yebC* | putative cytoplasmic protein | -3.876 | 0.942 |
| STM2316 | *nuoN* | NADH dehydrogenase I chain N | -3.877 | 1.703 |
| STM1336 | *rplT* | 50S ribosomal subunit protein L20 | -3.878 | 1.022 |
| STM3054 | *gcvH* | glycine cleavage complex protein H, carrier of aminomethyl moiety via covalently bound lipoyl cofactor | -3.886 | 4.252 |
| STM0397 | *phoB* | response regulator in two-component regulatory system with PhoR (or CreC), regulates pho regulon (OmpR family) | -3.902 | 0.731 |
| STM4102 | *STM4102* | putative inner membrane protein | -3.908 | 5.708 |
| STM0792 | *ybhB* | putative Phospholipid-binding protein | -3.911 | 1.268 |
| STM3307 | *murA* | UDP-N-acetylglucosamine 1-carboxyvinyltransferase | -3.912 | 0.829 |
| PSLT046 | *PSLT046* | putative carbonic anhydrase | -3.944 | 0.897 |
| STM3108 | *yggL* | putative cytoplasmic protein | -3.952 | 0.720 |
| STM2318 | *nuoL* | NADH dehydrogenase I chain L | -3.966 | 0.897 |
| STM4330 | *mopA* | chaperone Hsp60 with peptide-dependent ATPase activity, affects cell division | -3.991 | 1.490 |
| STM1223 | *potC* | ABC superfamily (membrane), spermidine/putrescine transporter | -4.038 | 2.063 |
| STM3717 | *rfaJ* | UDP-D-glucose:(galactosyl)lipopolysaccharide glucosyltransferase | -4.045 | 0.977 |
| STM0998 | *aspC* | aspartate aminotransferase | -4.050 | 1.310 |
| STM3702 | *grxC* | glutaredoxin 3 | -4.059 | 1.275 |
| STM1529 | *STM1529* | putative inner membrane protein | -4.072 | 0.783 |
| STM0141 | *guaC* | GMP reductase | -4.097 | 1.210 |
| STM2062 | *dacD* | DD-carboxypeptidase, penicillin-binding protein 6b | -4.117 | 3.317 |
| STM1419 | *yscR* | Secretion system apparatus: homology with YscR of the secretion system of Yersinia | -4.117 | 0.779 |
| STM1900 | *ntpA* | dATP pyrophosphohydrolase | -4.129 | 3.836 |
| STM2510 | *guaA* | GMP synthetase | -4.136 | 2.627 |
| STM0746 | *tolR* | tol protein, role in outer membrane integrity, uptake of group A colicins (TonB independent), and translocation of phage DNA to cytoplasm | -4.176 | 1.015 |
| STM1814 | *minC* | cell division inhibitor; activated MinC inhibits FtsZ ring formation | -4.177 | 1.151 |
| STM1816 | *minE* | cell division topological specificity factor, reverses MinC inhibition of FtsZ ring formation | -4.177 | 2.153 |
| STM0130 | *ddlB* | D-alanine-D-alanine ligase B, affects cell division | -4.205 | 1.465 |
| STM3359 | *mdh* | malate dehydrogenase | -4.218 | 1.645 |
| STM1533 | *STM1533* | putative hydrogenase | -4.235 | 1.419 |
| STM4268 | *yjcD* | putative xanthine/uracil permease family | -4.256 | 2.534 |
| STM4150 | *rplA* | 50S ribosomal subunit protein L1, regulates synthesis of L1 and L11 | -4.257 | 1.338 |
| STM0113 | *leuA* | 2-isopropylmalate synthase | -4.261 | 1.935 |
| STM2089 | *rfbJ* | LPS side chain defect: CDP-abequose synthase | -4.280 | 1.117 |
| STM3871 | *atpB* | membrane-bound ATP synthase, F0 sector, subunit a, important for FO assembly | -4.291 | 0.510 |
| STM2781 | *virK* | virulence gene; homologous sequence to virK in Shigella | -4.294 | 1.548 |
| STM2083 | *rfbK* | LPS side chain defect: phosphomannomutase | -4.308 | 1.170 |
| STM4393 | *rpsR* | 30S ribosomal subunit protein S18 | -4.315 | 0.986 |
| STM0602 | *ybdH* | putative glycerol dehydrogenase | -4.316 | 0.437 |
| STM0797 | *bioD* | dethiobiotin synthetase | -4.333 | 2.143 |
| STM4414 | *ppa* | inorganic pyrophosphatase | -4.339 | 1.793 |
| STM1857 | *STM1857* | putative acetyltransferase | -4.339 | 2.433 |
| STM3040 | *lysS* | lysine tRNA synthetase, constitutive | -4.341 | 0.696 |
| STM4319 | *phoN* | non-specific acid phosphatase | -4.389 | 0.723 |
| STM0407 | *secD* | preprotein translocase, IISP family, part of the channel | -4.398 | 1.103 |
| STM4188 | *metH* | B12-dependent homocysteine-N5-methyltetrahydrofolate transmethylase, repressor of metE and metF | -4.404 | 1.643 |
| STM0244 | *rcsF* | regulator in colanic acid synthesis; overexpression confers mucoid phenotype, increases capsule synthesis | -4.419 | 0.910 |
| STM0849 | *yliB* | putative ABC transporter periplasmic binding protein | -4.423 | 1.064 |
| STM2565 | *purG* | phosphoribosylformylglycinamidine synthetase | -4.430 | 4.894 |
| STM0224 | *yaeT* | putative outer membrane antigen | -4.431 | 1.464 |
| STM2322 | *nuoH* | NADH dehydrogenase I chain H | -4.440 | 1.686 |
| STM0978 | *aroA* | 3-enolpyruvylshikimate-5-phosphate synthetase | -4.448 | 1.991 |
| STM1631 | *sseJ* | Salmonella translocated effector: regulated by SPI-2 | -4.469 | 0.289 |
| STM0648 | *leuS* | leucine tRNA synthetase | -4.475 | 1.017 |
| STM2296 | *ais* | aluminum inducible protein | -4.480 | 1.630 |
| STM4458 | *yjgF* | putative translation initiation inhibitor | -4.488 | 0.719 |
| STM2673 | *rplS* | 50S ribosomal subunit protein L19 | -4.488 | 1.742 |
| STM0536 | *ppiB* | peptidyl-prolyl cis-trans isomerase B (rotamase B) | -4.500 | 0.926 |
| STM0891 | *artP* | ABC superfamily (atp&memb), arginine transport system | -4.502 | 0.628 |
| STM2327 | *nuoB* | NADH dehydrogenase I chain B | -4.519 | 1.391 |
| STM0247 | *abc* | putative ABC superfamily (atp_bind) transport system | -4.556 | 0.686 |
| STM1411 | *ssaK* | Secretion system apparatus | -4.631 | 0.314 |
| STM0129 | *murC* | L-alanine adding enzyme, UDP-N-acetyl-muramate:alanine ligase | -4.667 | 1.341 |
| STM1891 | *znuA* | ABC superfamily (bind_prot) high affinity Zn transport protein | -4.695 | 1.338 |
| STM0887 | *artJ* | ABC superfamily (bind_prot), arginine 3rd transport system | -4.715 | 1.720 |
| STM3869 | *atpF* | membrane-bound ATP synthase, F0 sector, subunit b | -4.740 | 1.162 |
| STM2323 | *nuoG* | NADH dehydrogenase I chain G | -4.748 | 0.925 |
| STM1230 | *phoQ* | sensory kinase protein in two-component regulatory system with PhoP, ligand is Mg+ | -4.763 | 1.622 |
| STM0154 | *lpdA* | lipoamide dehydrogenase (NADH); component of 2-oxodehydrogenase and pyruvate complexes; L protein of glycine cleavage complex second part | -4.797 | 1.759 |
| STM0977 | *serC* | 3-phosphoserine aminotransferase / phosphohydroxythreonine transaminase | -4.819 | 1.469 |
| STM2338 | *pta* | phosphotransacetylase | -4.819 | 1.588 |
| STM1341 | *btuE* | ABC superfamily (binding protein), vitamin B12 transport protein | -4.853 | 0.826 |
| STM3868 | *atpH* | membrane-bound ATP synthase, F1 sector, delta-subunit | -4.856 | 0.950 |
| STM1401 | *sseD* | Secretion system effector | -4.859 | 0.248 |
| STM1536 | *STM1536* | putative hydrogenase maturation protease | -4.877 | 1.975 |
| STM0246 | *yaeE* | putative ABC superfamily (membrane) transport protein | -4.917 | 1.232 |
| STM1535 | *STM1535* | putative hydrogenase protein | -4.939 | 2.036 |
| STM0973 | *pflB* | pyruvate formate lyase I, induced anaerobically | -4.945 | 1.775 |
| STM0312 | *yafK* | putative periplasmic protein | -4.953 | 1.698 |
| STM1377 | *lpp* | murein lipoprotein, links outer and inner membranes | -4.963 | 8.571 |
| STM4101 | *metL* | aspartokinase II in bifunctional enxyme: aspartokinase II; homoserine dehydrogenase II | -4.970 | 1.451 |
| STM1165 | *grxB* | glutaredoxin 2 | -5.005 | 1.262 |
| STM4081 | *tpiA* | triosephosphate isomerase | -5.041 | 0.935 |
| STM2325 | *nuoE* | NADH dehydrogenase I chain E | -5.065 | 1.470 |
| STM2155 | *metG* | methionine tRNA synthetase | -5.097 | 2.189 |
| STM3656 | *glyQ* | glycine tRNA synthetase, alpha subunit | -5.104 | 1.842 |
| STM0184 | *pcnB* | poly(A) polymerase I | -5.201 | 1.991 |
| STM2988 | *mltA* | membrane-bound lytic murein transglycosylase A | -5.216 | 1.628 |
| STM2084 | *rfbM* | LPS side chain defect: mannose-1-phosphate guanylyltransferase | -5.219 | 1.309 |
| STM2555 | *glyA* | serine hydroxymethyltransferase | -5.221 | 1.692 |
| STM1274 | *yeaQ* | putative inner membrane protein | -5.222 | 5.999 |
| STM4064 | *cdh* | CDP-diacylglycerol phosphotidylhydrolase | -5.224 | 1.515 |
| STM0980 | *cmk* | cytidine monophosphate (CMP) kinase | -5.231 | 0.966 |
| STM2287 | *STM2287* | putative cytoplasmic protein | -5.263 | 0.727 |
| STM2991 | *amiC* | N-acetylmuramoyl-L-alanine amidase | -5.281 | 1.169 |
| STM4119 | *ppc* | phosphoenolpyruvate carboxylase | -5.285 | 1.933 |
| STM3893 | *9S* | regulatory RNA | -5.325 | 3.296 |
| STM3866 | *atpG* | membrane-bound ATP synthase, F1 sector, gamma-subunit | -5.380 | 0.690 |
| STM2931 | *ygbQ* | putative Septum formation initiator | -5.380 | 2.465 |
| STM1815 | *minD* | cell division inhibitor, a membrane ATPase, activates MinC, directs division apparatus to middle of cell by oscillating from one half to other | -5.380 | 0.743 |
| STM1572 | *nmpC* | new outer membrane protein; predicted bacterial porin | -5.383 | 1.036 |
| STM2952 | *eno* | enolase | -5.421 | 1.875 |
| STM1473 | *ompN* | outer membrane protein N, non-specific porin | -5.451 | 0.403 |
| STM1290 | *gapA* | glyceraldehyde-3-phosphate dehydrogenase A | -5.463 | 1.933 |
| STM2324 | *nuoF* | NADH dehydrogenase I chain F | -5.478 | 1.401 |
| STM4009 | *typA* | GTP-binding elongation factor family protein | -5.493 | 2.549 |
| STM3538 | *glgB* | 1,4-alpha-glucan branching enzyme | -5.501 | 1.450 |
| STM1299 | *gdhA* | glutamate dehydrogenase, NADP-specific | -5.508 | 2.147 |
| STM0750 | *ybgF* | putative periplasmic protein | -5.512 | 2.111 |
| STM2674 | *trmD* | tRNA (guanine-7-)-methyltransferase | -5.518 | 2.161 |
| STM1633 | *STM1633* | putative periplasmic binding protein | -5.520 | 1.862 |
| STM3865 | *atpD* | membrane-bound ATP synthase, F1 sector, beta-subunit | -5.531 | 0.591 |
| STM3728 | *rpmB* | 50S ribosomal subunit protein L28 | -5.543 | 3.399 |
| STM3112 | *mltC* | membrane-bound lytic murein transglycosylase C | -5.547 | 1.651 |
| STM0110 | *leuD* | 3-isopropylmalate isomerase (dehydratase), subunit with LeuC | -5.623 | 5.133 |
| STM2782 | *mig-14* | putative transcription activator | -5.676 | 2.257 |
| STM1412 | *ssaL* | Secretion system apparatus | -5.707 | 0.567 |
| STM2194 | *yeiG* | putative esterase | -5.760 | 1.983 |
| STM3867 | *atpA* | membrane-bound ATP synthase, F1 sector, alpha-subunit | -5.781 | 0.955 |
| STM0603 | *ybdL* | putative aminotransferase | -5.782 | 0.798 |
| STM0111 | *leuC* | 3-isopropylmalate isomerase (dehydratase), subunit with LeuD | -5.797 | 2.030 |
| STM2320 | *nuoJ* | NADH dehydrogenase I chain J | -5.802 | 9.301 |
| STM3423 | *rpsE* | 30S ribosomal subunit protein S5 | -5.870 | 2.179 |
| STM3062 | *serA* | D-3-phosphoglycerate dehydrogenase | -5.887 | 1.356 |
| STM0749 | *pal* | tol protein required for outer membrane integrity, uptake of group A colicins, and translocation of phage DNA to cytoplasm | -5.913 | 1.529 |
| STM4151 | *rplJ* | 50S ribosomal subunit protein L10 | -5.916 | 2.359 |
| STM0245 | *yaeC* | putative outer membrane lipoprotein | -5.959 | 0.933 |
| STM3703 | *yibN* | putative Rhodanese-related sulfurtransferases | -5.968 | 1.717 |
| STM3421 | *rplO* | 50S ribosomal subunit protein L15 | -5.969 | 2.326 |
| STM0217 | *tsf* | protein chain elongation factor EF-Ts | -5.985 | 1.933 |
| STM0225 | *hlpA* | histone-like protein, located in outer membrane | -6.028 | 2.559 |
| STM3424 | *rplR* | 50S ribosomal subunit protein L18 | -6.038 | 2.463 |
| STM2443 | *cysU* | ABC superfamily (membrane), thiosulfate transport protein | -6.053 | 1.648 |
| STM4055 | *sodA* | superoxide dismutase, manganese | -6.099 | 1.201 |
| STM3420 | *secY* | preprotein translocase of IISP family, membrane subunit, putative ATPase | -6.100 | 2.060 |
| STM2441 | *cysA* | ABC superfamily (atp_bind), sulfate permease A protein; chromate resistance | -6.114 | 1.617 |
| STM1901 | *aspS* | aspartate tRNA synthetase | -6.114 | 1.539 |
| STM4153 | *rpoB* | RNA polymerase, beta subunit | -6.140 | 2.718 |
| STM1779 | *ipk* | isopentenyl monophosphate kinase | -6.162 | 1.714 |
| STM2272 | *gyrA* | DNA gyrase, subunit A, type II topoisomerase | -6.239 | 1.951 |
| STM2094 | *rfbC* | dTDP-4,deoxyrhamnose 3,5 epimerase | -6.242 | 1.202 |
| STM4154 | *rpoC* | RNA polymerase, beta prime subunit | -6.249 | 2.152 |
| STM0216 | *rpsB* | 30S ribosomal subunit protein S2 | -6.306 | 2.417 |
| STM1418 | *ssaQ* | Secretion system apparatus | -6.308 | 1.516 |
| STM0380 | *ddlA* | D-alanine-D-alanine ligase A | -6.335 | 2.237 |
| STM3380 | *accC* | acetyl CoA carboxylase, biotin carboxylase subunit | -6.356 | 1.665 |
| STM3419 | *rpmJ* | 50S ribosomal subunit protein X | -6.376 | 2.882 |
| STM1088 | *pipB* | Pathogenicity island encoded protein: SPI3 | -6.392 | 1.273 |
| STM0116 | *ilvI* | acetolactate synthase III, valine sensitive, large subunit | -6.397 | 1.595 |
| STM3384 | *yhdG* | putative TIM-barrel enzyme, possibly dehydrogenase | -6.398 | 3.756 |
| STM1191 | *rpmF* | 50S ribosomal subunit protein L32 | -6.552 | 2.504 |
| STM3965 | *metE* | 5-methyltetrahydropteroyltriglutamate-homocysteine S-methyltransferase | -6.662 | 2.554 |
| STM3486 | *aroB* | dehydroquinate synthase | -6.880 | 2.036 |
| STM2241 | *sspH2* | Leucine-rich repeat protein, induced by the SPI-2 regulator ssrA/B | -6.908 | 0.453 |
| STM3431 | *rpsQ* | 30S ribosomal subunit protein S17 | -7.009 | 2.778 |
| STM3452 | *yheO* | putative regulator | -7.020 | 3.297 |
| STM1420 | *ssaS* | Secretion system apparatus: homology with YscS of the secretion system of Yersinia | -7.155 | 2.822 |
| STM4170 | *hupA* | DNA-binding protein HU-alpha (HU-2) | -7.242 | 1.952 |
| STM0890 | *artI* | ABC superfamily (bind_prot), arginine transport system | -7.246 | 2.697 |
| STM4105 | *metF* | 5,10-methylenetetrahydrofolate reductase | -7.304 | 1.362 |
| STM3429 | *rplX* | 50S ribosomal subunit protein L24 | -7.381 | 3.643 |
| STM3425 | *rplF* | 50S ribosomal subunit protein L6 | -7.392 | 5.766 |
| STM1780 | *prsA* | phosphoribosylpyrophosphate synthetase | -7.437 | 1.675 |
| STM3331 | *gltD* | glutamate synthase, small subunit | -7.459 | 3.277 |
| STM0406 | *yajC* | preprotein translocase IISP family, membrane subunit | -7.459 | 1.990 |
| STM3435 | *rplV* | 50S ribosomal subunit protein L22 | -7.502 | 4.246 |
| STM1838 | *yobF* | putative cytoplasmic protein | -7.519 | 3.737 |
| STM3445 | *tufA* | protein chain elongation factor EF-Tu (duplicate of tufB) | -7.547 | 2.824 |
| STM3487 | *aroK* | shikimate kinase I | -7.599 | 2.641 |
| STM2442 | *cysW* | ABC superfamily (membrane), thiosulfate permease W protein | -7.648 | 2.627 |
| STM0958 | *trxB* | thioredoxin reductase | -7.710 | 2.045 |
| STM0637 | *dacA* | D-alanyl-D-alanine carboxypeptidase, penicillin-binding protein 5 | -7.760 | 3.436 |
| STM3875 | *mioC* | initiation of chromosome replication | -7.815 | 4.047 |
| STM3418 | *rpsM* | 30S ribosomal subunit protein S13 | -7.824 | 1.559 |
| STM1475 | *rstA* | response regulator in two-component regulatory system with RstB (OmpR family) | -7.922 | 3.352 |
| STM3665 | *avtA* | valine-pyruvate aminotransferase | -7.924 | 10.405 |
| STM3845 | *STM3845* | putative inner membrane protein | -7.927 | 6.671 |
| STM0112 | *leuB* | 3-isopropylmalate dehydrogenase | -8.049 | 3.274 |
| STM3447 | *rpsG* | 30S ribosomal subunit protein S7, initiates assembly | -8.102 | 2.531 |
| STM0445 | *yajG* | putative lipoprotein | -8.175 | 6.637 |
| STM2946 | *cysH* | 3'-phosphoadenosine 5'-phosphosulfate (PAPS) reductase | -8.295 | 1.240 |
| STM2328 | *nuoA* | NADH dehydrogenase I chain A | -8.330 | 3.426 |
| STM1190 | *yceD* | putative metal-binding | -8.477 | 3.184 |
| STM4146 | *tufB* | protein chain elongation factor EF-Tu (duplicate of tufA) | -8.500 | 3.080 |
| STM3446 | *fusA* | protein chain elongation factor EF-G, GTP-binding | -8.563 | 2.815 |
| STM1378 | *pykF* | pyruvate kinase I (formerly F), fructose stimulated | -8.645 | 1.112 |
| STM2337 | *ackA* | acetate kinase A (propionate kinase 2) | -8.661 | 3.540 |
| STM2267 | *ompC* | outer membrane protein 1b (ib;c), porin | -8.764 | 1.892 |
| STM3433 | *rplP* | 50S ribosomal subunit protein L16 | -8.766 | 2.641 |
| STM2947 | *cysI* | sulfite reductase, alpha subunit, NADPH dependent | -8.794 | 3.462 |
| STM1413 | *ssaM* | Secretion system apparatus | -8.859 | 10.332 |
| STM1394 | *ssaC* | Secretion system apparatus | -8.998 | 2.008 |
| STM2827 | *alaS* | alanyl-tRNA synthetase | -9.017 | 2.565 |
| STM4220 | *lysC* | aspartokinase III, lysine sensitive | -9.030 | 3.943 |
| STM2948 | *cysJ* | sulfite reductase, beta (flavoprotein) subunit | -9.051 | 3.466 |
| STM0981 | *rpsA* | 30S ribosomal subunit protein S1 | -9.087 | 3.529 |
| STM0171 | *yadF* | putative carbonic anhydrase | -9.093 | 3.329 |
| STM3434 | *rpsC* | 30S ribosomal subunit protein S3 | -9.111 | 3.158 |
| STM3290 | *argG* | argininosuccinate synthetase | -9.135 | 3.711 |
| STM1224 | *sifA* | lysosomal glycoprotein (lgp)-containing structures; replication in macrophages | -9.203 | 5.092 |
| STM3427 | *rpsN* | 30S ribosomal subunit protein S14 | -9.244 | 6.734 |
| STM3330 | *gltB* | glutamate synthase, large subunit | -9.255 | 4.047 |
| STM2317 | *nuoM* | NADH dehydrogenase I chain M | -9.290 | 2.858 |
| STM1320 | *ydjN* | part of a kinase, putative domain shared with transporter | -9.428 | 2.528 |
| STM2299 | *yfbG* | paral putative transformylase | -9.445 | 4.475 |
| STM4392 | *priB* | primosomal replication protein N | -9.478 | 4.188 |
| STM3379 | *accB* | acetylCoA carboxylase, BCCP subunit, carrier of biotin | -9.570 | 3.728 |
| STM1414 | *ssaV* | Secretion system apparatus: homology with the LcrD family of proteins | -9.584 | 2.469 |
| STM3539 | *asd* | aspartate-semialdehyde dehydrogenase | -9.725 | 3.556 |
| STM0959 | *lrp* | regulator for lrp regulon and high-affinity branched-chain amino acid transport system; mediator of of leucine response (AsnC family) | -10.374 | 3.780 |
| STM4149 | *rplK* | 50 S ribosomal subunit protein L11 | -10.391 | 3.505 |
| STM3448 | *rpsL* | 30S ribosomal subunit protein S12 | -10.459 | 4.297 |
| STM3345 | *rplM* | 50S ribosomal subunit protein L13 | -10.473 | 4.356 |
| STM2933 | *cysC* | adenosine 5'-phosphosulfate kinase | -10.524 | 4.067 |
| STM1395 | *ssaD* | Secretion system apparatus | -10.683 | 3.491 |
| STM1403 | *sscB* | Secretion system chaparone | -10.812 | 2.019 |
| STM3428 | *rplE* | 50S ribosomal subunit protein L5 | -10.950 | 4.509 |
| STM2298 | *pmrF* | putative glycosyl transferase | -11.090 | 5.390 |
| STM3438 | *rplW* | 50S ribosomal subunit protein L23 | -11.227 | 3.283 |
| STM2326 | *nuoC* | NADH dehydrogenase I chain C,D | -11.325 | 4.811 |
| STM2934 | *cysN* | ATP-sulfurylase, subunit 1 (ATP:sulfate adenylyltransferase) | -11.486 | 3.837 |
| STM0064 | *dapB* | dihydrodipicolinate reductase | -11.573 | 5.262 |
| STM1402 | *sseE* | Secretion system effector | -11.636 | 2.508 |
| STM1222 | *potD* | ABC superfamily (peri_perm), spermidine/putrescine transporter | -11.647 | 2.193 |
| STM3090 | *metK* | methionine adenosyltransferase 1 (AdoMet synthetase) | -11.838 | 4.273 |
| STM0996 | *ycbK* | putative outer membrane protein | -11.861 | 5.599 |
| STM1417 | *ssaP* | Secretion system apparatus | -12.303 | 3.226 |
| STM1415 | *ssaN* | Secretion system apparatus: homology with the YscN family of proteins | -12.649 | 2.690 |
| STM1416 | *ssaO* | Secretion system apparatus | -13.206 | 13.254 |
| STM3417 | *rpsK* | 30S ribosomal subunit protein S11 | -13.571 | 2.784 |
| STM1698 | *STM1698* | putative inner membrane protein | -13.706 | 1.412 |
| STM3468 | *argD* | acetylornithine transaminase (NAcOATase and DapATase) | -13.789 | 6.832 |
| STM2444 | *cysP* | ABC superfamily (bind_prot), thiosulfate transport protein | -13.863 | 5.674 |
| STM1602 | *sifB* | Salmonella translocated effector: translocated by SPI-2 | -14.077 | 5.438 |
| STM1421 | *ssaT* | Secretion system apparatus: homology with YscT of the secretion system of Yersinia | -15.044 | 6.732 |
| STM0877 | *potF* | ABC superfamily (peri_perm), putrescine transporter | -15.212 | 6.283 |
| STM3416 | *rpsD* | 30S ribosomal subunit protein S4 | -15.220 | 4.667 |
| STM3440 | *rplC* | 50S ribosomal subunit protein L3 | -15.669 | 6.541 |
| STM2487 | *purC* | phosphoribosylaminoimidazole-succinocarboxamide synthetase (SAICAR synthetase) | -15.980 | 15.282 |
| STM1070 | *ompA* | putative hydrogenase, membrane component | -16.261 | 4.393 |
| STM4100 | *metB* | cystathionine gamma-synthase | -16.346 | 4.073 |
| STM1530 | *STM1530* | putative outer membrane protein | -16.626 | 5.549 |
| STM2675 | *rimM* | 16S rRNA processing protein | -16.743 | 7.005 |
| STM3437 | *rplB* | 50S ribosomal subunit protein L2 | -16.770 | 6.647 |
| STM3088 | *yqgC* | putative cytoplasmic protein | -17.099 | 12.571 |
| STM2430 | *cysK* | subunit of cysteine synthase A and O-acetylserine sulfhydrolase A | -17.165 | 6.097 |
| STM4163 | *thiE* | thiamin phosphate synthase (thiamine phosphate pyrophosphorylase) | -17.273 | 6.792 |
| STM2676 | *rpsP* | 30S ribosomal subunit protein S16 | -17.645 | 18.593 |
| STM1232 | *purB* | adenylosuccinate lyase | -17.874 | 2.789 |
| STM3430 | *rplN* | 50S ribosomal subunit protein L14 | -18.221 | 8.482 |
| STM3439 | *rplD* | 50S ribosomal subunit protein L4, regulates expression of S10 operon | -18.808 | 8.300 |
| STM3426 | *rpsH* | 30S ribosomal subunit protein S8, and regulator | -18.877 | 8.796 |
| STM4160 | *thiG* | deoxyxylulose-5-P + thi-S-COSH + tyrosine = 4-methyl-5-(beta-hydroxyethyl)thiazole-P + 4-hydroxy-benzyl-alcohol + C1 of tyrosine | -18.954 | 4.299 |
| STM3415 | *rpoA* | RNA polymerase, alpha subunit | -21.431 | 7.807 |
| STM4182 | *metA* | homoserine transsuccinylase | -21.597 | 5.202 |
| STM2080 | *udg* | UDP-glucose/GDP-mannose dehydrogenase | -23.381 | 11.547 |
| STM3436 | *rpsS* | 30S ribosomal subunit protein S19 | -24.251 | 25.229 |
| STM4159 | *thiH* | deoxyxylulose-5-P + thi-S-COSH + tyrosine = 4-methyl-5-(beta-hydroxyethyl)thiazole-P + 4-hydroxy-benzyl-alcohol + C1 of tyrosine | -34.351 | 8.934 |
| STM4164 | *thiC* | 5'-phosphoryl-5-aminoimidazole = 4-amino-5-hydroxymethyl-2-methylpyrimidine-P | -36.901 | 11.882 |
| STM2935 | *cysD* | ATP-sulfurylase, subunit 1 (ATP:sulfate adenylyltransferase) | -47.117 | 21.851 |
| STM3414 | *rplQ* | 50S ribosomal subunit protein L17 | -50.350 | 12.711 |
