# Supplementary material for: Nitric Oxide Antagonizes the Acid Tolerance Response that Protects Salmonella against Innate Gastric Defenses
Source: PLoS One. 2008 Mar 19;3(3):e1833. doi: 10.1371/journal.pone.0001833 (PMC2266805; doi:10.1371/journal.pone.0001833)
Supplement: Table S2 — (0.07 MB DOC) [file pone.0001833.s002.doc]

**Table S2**

**Selected groups of induced *Salmonella* genes in response to RNS under adapting pH 4.4**

| **STM gene ID no.** | **Gene Name** | **Description** | **Fold change** | **SD** |
| --- | --- | --- | --- | --- |

Iron Acquisition

| STM0364 | *foxA* | ferrioxamine receptor | 3.44 | 0.14 |
| --- | --- | --- | --- | --- |
| STM0592 | *fepD* | ABC superfamily (membrane), ferric enterobactin (enterochelin) transporter | 4.35 | 0.23 |
| STM2773 | *iroB* | putative glycosyl transferase, related to UDP-glucuronosyltransferase | 25.24 | 0.03 |
| STM2775 | *iroD* | Similar to enterochelin esterase of E. coli (Fes) | 5.23 | 0.30 |
| STM2776 | *iroE* | putative hydrolase of the alpha/beta superfamily | 4.13 | 0.08 |
| STM2777 | *iroN* | TonB-dependent siderophore receptor protein | 179.20 | 0.00 |
| STM2861 | *sitA* | Salmonella iron transporter: fur regulated | 34.44 | 0.00 |
| STM2862 | *sitB* | Salmonella iron transporter: fur regulated | 18.68 | 0.01 |
| STM2863 | *sitC* | Salmonella iron transporter: fur regulated | 8.53 | 0.03 |
| STM2864 | *sitD* | Salmonella iron transporter: fur regulated | 6.55 | 0.05 |

SOS Response

| STM1998 | *umuD* | error-prone repair: SOS-response transcriptional repressors (LexA homologs, RecA-mediated autopeptidases) | 11.90 | 0.06 |
| --- | --- | --- | --- | --- |
| STM4237 | *lexA* | SOS response regulator, transcriptional repressor (LexA family) | 10.07 | 0.03 |
| STM2684 | *recN* | protein used in recombination and DNA repair | 8.29 | 0.04 |
| STM1997 | *umuC* | error-prone repair: component of DNA polymerase V with UmuD' | 5.32 | 0.07 |
| STM3591 | *uspA* | universal stress protein A | 4.80 | 0.12 |
| STM1162 | *dinI* | DNA damage-inducible protein I, inhibits UmuD processing | 4.45 | 0.14 |
| STM0097 | *polB* | DNA polymerase II and and 3' --> 5' exonuclease | 3.72 | 0.05 |
| STM1369 | *sufA* | putative HesB-like domain | 6.04 | 0.02 |
| STM1882 | *yebG* | DNA damage-inducible gene in SOS regulon, dependent on cyclic AMP and H-NS | 28.73 | 0.01 |

Nitrogen Metabolism

| STM2556 | *hmpA* | dihydropteridine reductase 2 and nitric oxide dioxygenase activity | 72.10 | 0.01 |
| --- | --- | --- | --- | --- |
| STM1762 | *narJ* | nitrate reductase 1, delta subunit, chaperone required for molybdenum cofactor assembly in nitrate reductase 1 | 2.91 | 0.24 |
| STM1569 | *fdnH* | formate dehydrogenase-N, Fe-S beta subunit, nitrate-inducible | 3.03 | 0.14 |
| STM3476 | *nirC* | FNT family, nitrite transport protein | 5.27 | 0.33 |
| STM4277 | *nrfA* | nitrite reductase periplasmic cytochrome c(552) | 3.56 | 0.26 |
| STM4279 | *nrfC* | putative nitrite reductase; formate-dependent, Fe-S centers | 3.14 | 0.15 |

Motility, Adherence and Invasion

| STM2876 | *hilA* | Invasion gene transcriptional activator | 4.47 | 0.41 |
| --- | --- | --- | --- | --- |
| STM2896 | *invA* | Invasion protein | 3.75 | 0.14 |
| STM2895 | *invB* | Surface presentation of antigens: secretory protein | 3.52 | 0.20 |
| STM2894 | *invC* | Surface presentation of antigens: secretory protein | 3.09 | 0.12 |
| STM1091 | *sopB* | *Salmonella* outer protein: homologous to *ipgD* of *Shigella* | 3.05 | 0.15 |
| STM1855 | *sopE2* | Type III-secreted protein effector: invasion associated protein | 4.10 | 0.33 |
| STM2886 | *sicA* | Surface presentation of antigens: secretory protein | 4.13 | 0.11 |
| STM2877 | *iagB* | cell invasion protein | 3.23 | 0.151 |
| STM2865 | *avrA* | putative inner protein | 3.47 | 0.13 |
| STM1174 | *flgB* | flagellar biosynthesis, cell-proximal portion of basal-body rod | 3.60 | 0.177 |
| STM1178 | *flgF* | flagellar biosynthesis, cell-proximal portion of basal-body rod | 5.29 | 0.276 |
| STM1181 | *flgI* | putative flagella basal body protein | 2.57 | 0.201 |
| STM1912 | *flhE* | flagellar protein | 5.77 | 0.299 |
| STM1959 | *fliC* | Flagella biosynthesis; flagellin; filament structural protein | 8.47 | 0.027 |
| STM1960 | *fliD* | Flagella biosynthesis; filament capping protein | 2.91 | 0.115 |
| STM1981 | *fliR* | putative flagellar biosynthetic protein | 4.03 | 0.157 |
| STM2771 | *fljB* | Flagellar synthesis: phase 2 flagellin (filament structural protein) | 5.11 | 0.134 |
| PSLT018 | *pefA* | plasmid-encoded fimbriae; major fimbrial subunit | 4.80 | 0.124 |
| PSLT082 | *traP* | conjugative transfer | 4.16 | 0.176 |
| PSLT084 | *traV* | conjugative transfer: assembly | 3.03 | 0.230 |
| PSLT098 | *traQ* | conjugative transfer: fimbrial synthesis | 3.03 | 0.169 |
| PSLT102 | *traS* | conjugative transfer: surface exclusion | 9.85 | 0.034 |
| PSLT105 | *trbH* | conjugative transfer | 7.99 | 0.061 |
| STM0143 | *hofB* | putative integral membrane protein involved in biogenesis of fimbriae (type IV pilin), protein transport, DNA uptake | 3.10 | 0.211 |
| STM0144 | *ppdD* | putative major component of type IV pilin, prelipin peptidase dependent protein | 7.30 | 0.132 |
| STM0195 | *stfA* | putative fimbrial subunit | 2.76 | 0.143 |
| STM0200 | *stfG* | putative minor fimbrial subunit; putative adhesin | 13.72 | 0.234 |
| STM0300 | *safB* | putative fimbriae assembly chaparone | 6.34 | 0.196 |
| STM0549 | *fimZ* | fimbrial protein Z, putative transcriptional regulator (LuxR/UhpA family) | 3.78 | 0.102 |
| STM0550 | *fimY* | putative regulatory protein | 6.37 | 0.208 |
| STM0552 | *fimW* | putative fimbrial protein | 4.81 | 0.145 |
| STM1140 | *csgF* | curli production assembly/transport component, 2nd curli operon | 5.82 | 0.084 |
| STM1141 | *csgE* | curli production assembly/transport component, 2nd curli operon | 9.51 | 0.363 |
| STM1142 | *csgD* | putative transcriptional regulator (LuxR/UhpA family) | 4.78 | 0.135 |
| STM1143 | *csgB* | minor curlin subunit precursor, nucleator for assembly of adhesive surface organelles | 2.68 | 0.166 |
| STM1144 | *csgA* | curlin major subunit, coiled surface structures; cryptic | 3.75 | 0.155 |
| STM1145 | *csgC* | putative curli production protein | 4.21 | 0.186 |
| STM2997 | *ppdC* | prepilin peptidase dependent protein C, putative component in type IV pilin biogenesis | 5.01 | 0.163 |
| STM3028 | *stdB* | putative outer membrane usher protein | 4.48 | 0.173 |
| STM3029 | *stdA* | putative fimbrial-like protein | 4.57 | 0.283 |
| STM3637 | *lpfD* | long polar fimbrial operon protein | 2.50 | 0.157 |
| STM3640 | *lpfA* | long polar fimbria | 3.57 | 0.188 |
| STM4573 | *stjC* | putative fimbrial chaparone protein | 10.42 | 0.242 |
| STM4593 | *sthB* | putative fimbrial usher protein | 4.99 | 0.371 |
