# Supplementary material for: Nitric Oxide Antagonizes the Acid Tolerance Response that Protects Salmonella against Innate Gastric Defenses
Source: PLoS One. 2008 Mar 19;3(3):e1833. doi: 10.1371/journal.pone.0001833 (PMC2266805; doi:10.1371/journal.pone.0001833)
Supplement: Table S3 — (0.11 MB DOC) [file pone.0001833.s003.doc]

**Table S3**

**Selected groups of repressed *Salmonella* genes in response to RNS under adapting pH 4.4**

| **STM gene ID no.** | **Gene Name** | **Description** | **Fold change** | **SD** |
| --- | --- | --- | --- | --- |

SPI-2 regulon

| STM1224 | *sifA* | lysosomal glycoprotein (lgp)-containing structures; replication in macrophages | -9.20 | 5.09 |
| --- | --- | --- | --- | --- |
| STM1391 | *ssrB* | Secretion system regulator: transcriptonal activator, homologous with degU/uvrY/bvgA | -2.52 | 0.31 |
| STM1393 | *ssaB (spiC)* | Secretion system apparatus | -3.26 | 0.43 |
| STM1394 | *ssaC* | Secretion system apparatus | -9.00 | 2.01 |
| STM1395 | *ssaD* | Secretion system apparatus | -10.68 | 3.49 |
| STM1396 | *ssaE* | Secretion system effector | -3.64 | 2.87 |
| STM1400 | *sseC* | Secretion system effector | -2.36 | 0.16 |
| STM1401 | *sseD* | Secretion system effector | -4.86 | 0.25 |
| STM1402 | *sseE* | Secretion system effector | -11.64 | 2.51 |
| STM1403 | *sscB* | Secretion system chaparone | -10.81 | 2.02 |
| STM1408 | *ssaI* | Secretion system apparatus | -2.84 | 1.28 |
| STM1409 | *ssaJ* | Secretion system apparatus: homology with the yscJ/mxiJ/prgK family of lipoproteins | -3.21 | 0.46 |
| STM1411 | *ssaK* | Secretion system apparatus | -4.63 | 0.31 |
| STM1412 | *ssaL* | Secretion system apparatus | -5.71 | 0.57 |
| STM1413 | *ssaM* | Secretion system apparatus | -8.86 | 10.33 |
| STM1414 | *ssaV* | Secretion system apparatus: homology with the LcrD family of proteins | -9.58 | 2.47 |
| STM1415 | *ssaN* | Secretion system apparatus: homology with the YscN family of proteins | -12.65 | 2.69 |
| STM1417 | *ssaP* | Secretion system apparatus | -12.30 | 3.23 |
| STM1418 | *ssaQ* | Secretion system apparatus | -6.31 | 1.52 |
| STM1420 | *ssaS* | Secretion system apparatus: homology with YscS of the secretion system of Yersinia | -7.15 | 2.82 |
| STM1421 | *ssaT* | Secretion system apparatus: homology with YscT of the secretion system of Yersinia | -15.04 | 6.73 |
| STM1422 | *ssaU* | Secretion system apparatus: homology with YscU of the secretion system of Yersinia | -2.75 | 2.58 |
| STM1602 | *sifB* | Salmonella translocated effector: translocated by SPI-2 | -14.08 | 5.44 |
| STM1631 | *sseJ* | Salmonella translocated effector: regulated by SPI-2 | -4.47 | 0.29 |
| STM2241 | *sspH2* | Leucine-rich repeat protein, induced by the SPI-2 regulator ssrA/B | -6.91 | 0.45 |

Transcriptional-Translational Machinery

| STM0186 | *dksA* | dnaK suppressor protein | -3.60 | 1.14 |
| --- | --- | --- | --- | --- |
| STM0216 | *rpsB* | 30S ribosomal subunit protein S2 | -6.31 | 2.42 |
| STM0217 | *tsf* | protein chain elongation factor EF-Ts | -5.99 | 1.93 |
| STM0242 | *proS* | proline tRNA synthetase | -2.00 | 0.29 |
| STM0648 | *leuS* | leucine tRNA synthetase | -4.48 | 1.02 |
| STM0981 | *rpsA* | 30S ribosomal subunit protein S1 | -9.09 | 3.53 |
| STM1191 | *rpmF* | 50S ribosomal subunit protein L32 | -6.55 | 2.50 |
| STM1334 | *infC* | protein chain initiation factor IF-3 | -2.58 | 0.93 |
| STM1336 | *rplT* | 50S ribosomal subunit protein L20 | -3.88 | 1.02 |
| STM1449 | *tyrS* | tyrosine tRNA synthetase | -3.25 | 0.44 |
| STM1901 | *aspS* | aspartate tRNA synthetase | -6.11 | 1.54 |
| STM2155 | *metG* | methionine tRNA synthetase | -5.10 | 2.19 |
| STM2415 | *gltX* | glutamate tRNA synthetase, catalytic subunit | -2.63 | 0.48 |
| STM2430 | *cysK* | subunit of cysteine synthase A and O-acetylserine sulfhydrolase A | -17.16 | 6.10 |
| STM2440 | *cysM* | cysteine synthase B (O-acetylserine sulfhydrolase B) | -3.03 | 1.26 |
| STM2673 | *rplS* | 50S ribosomal subunit protein L19 | -4.49 | 1.74 |
| STM2674 | *trmD* | tRNA (guanine-7-)-methyltransferase | -5.52 | 2.16 |
| STM2675 | *rimM* | 16S rRNA processing protein | -16.74 | 7.00 |
| STM2676 | *rpsP* | 30S ribosomal subunit protein S16 | -17.65 | 18.59 |
| STM2827 | *alaS* | alanyl-tRNA synthetase | -9.02 | 2.56 |
| STM3040 | *lysS* | lysine tRNA synthetase, constitutive | -4.34 | 0.70 |
| STM3041 | *prfB* | peptide chain release factor RF-2 | -3.74 | 1.01 |
| STM3286 | *infB* | protein chain initiation factor IF-2 | -2.48 | 0.59 |
| STM3331 | *gltD* | glutamate synthase, small subunit | -7.46 | 3.28 |
| STM3345 | *rplM* | 50S ribosomal subunit protein L13 | -10.47 | 4.36 |
| STM3414 | *rplQ* | 50S ribosomal subunit protein L17 | -50.35 | 12.71 |
| STM3415 | *rpoA* | RNA polymerase, alpha subunit | -21.43 | 7.81 |
| STM3416 | *rpsD* | 30S ribosomal subunit protein S4 | -15.22 | 4.67 |
| STM3417 | *rpsK* | 30S ribosomal subunit protein S11 | -13.57 | 2.78 |
| STM3418 | *rpsM* | 30S ribosomal subunit protein S13 | -7.82 | 1.56 |
| STM3419 | *rpmJ* | 50S ribosomal subunit protein X | -6.38 | 2.88 |
| STM3421 | *rplO* | 50S ribosomal subunit protein L15 | -5.97 | 2.33 |
| STM3423 | *rpsE* | 30S ribosomal subunit protein S5 | -5.87 | 2.18 |
| STM3424 | *rplR* | 50S ribosomal subunit protein L18 | -6.04 | 2.46 |
| STM3425 | *rplF* | 50S ribosomal subunit protein L6 | -7.39 | 5.77 |
| STM3426 | *rpsH* | 30S ribosomal subunit protein S8, and regulator | -18.88 | 8.80 |
| STM3427 | *rpsN* | 30S ribosomal subunit protein S14 | -9.24 | 6.73 |
| STM3428 | *rplE* | 50S ribosomal subunit protein L5 | -10.95 | 4.51 |
| STM3429 | *rplX* | 50S ribosomal subunit protein L24 | -7.38 | 3.64 |
| STM3430 | *rplN* | 50S ribosomal subunit protein L14 | -18.22 | 8.48 |
| STM3431 | *rpsQ* | 30S ribosomal subunit protein S17 | -7.01 | 2.78 |
| STM3433 | *rplP* | 50S ribosomal subunit protein L16 | -8.77 | 2.64 |
| STM3434 | *rpsC* | 30S ribosomal subunit protein S3 | -9.11 | 3.16 |
| STM3435 | *rplV* | 50S ribosomal subunit protein L22 | -7.50 | 4.25 |
| STM3436 | *rpsS* | 30S ribosomal subunit protein S19 | -24.25 | 25.23 |
| STM3437 | *rplB* | 50S ribosomal subunit protein L2 | -16.77 | 6.65 |
| STM3438 | *rplW* | 50S ribosomal subunit protein L23 | -11.23 | 3.28 |
| STM3439 | *rplD* | 50S ribosomal subunit protein L4, regulates expression of S10 operon | -18.81 | 8.30 |
| STM3440 | *rplC* | 50S ribosomal subunit protein L3 | -15.67 | 6.54 |
| STM3445 | *tufA* | protein chain elongation factor EF-Tu (duplicate of tufB) | -7.55 | 2.82 |
| STM3446 | *fusA* | protein chain elongation factor EF-G, GTP-binding | -8.56 | 2.81 |
| STM3447 | *rpsG* | 30S ribosomal subunit protein S7, initiates assembly | -8.10 | 2.53 |
| STM3448 | *rpsL* | 30S ribosomal subunit protein S12 | -10.46 | 4.30 |
| STM3481 | *trpS* | tryptophan tRNA synthetase | -3.43 | 0.37 |
| STM3655 | *glyS* | glycine tRNA synthetase, beta subunit | -3.76 | 1.11 |
| STM3656 | *glyQ* | glycine tRNA synthetase, alpha subunit | -5.10 | 1.84 |
| STM3728 | *rpmB* | 50S ribosomal subunit protein L28 | -5.54 | 3.40 |
| STM3742 | *spoT* | bifunctional : (p)ppGpp synthetase II; also guanosine-3',5'-bis pyrophosphate 3'-pyrophosphohydrolase | -3.21 | 1.01 |
| STM3840 | *rnpA* | RNase P, protein component (protein C5), processes tRNA, 4.5S RNA | -2.92 | 1.42 |
| STM3877 | *asnA* | asparagine synthetase A | -2.36 | 0.80 |
| STM4027 | *rbn* | tRNA processing exoribonuclease BN | -2.08 | 0.44 |
| STM4146 | *tufB* | protein chain elongation factor EF-Tu (duplicate of tufA) | -8.50 | 3.08 |
| STM4149 | *rplK* | 50 S ribosomal subunit protein L11 | -10.39 | 3.51 |
| STM4150 | *rplA* | 50S ribosomal subunit protein L1, regulates synthesis of L1 and L11 | -4.26 | 1.34 |
| STM4151 | *rplJ* | 50S ribosomal subunit protein L10 | -5.92 | 2.36 |
| STM4152 | *rplL* | 50S ribosomal subunit protein L7/L12 | -3.23 | 1.02 |
| STM4153 | *rpoB* | RNA polymerase, beta subunit | -6.14 | 2.72 |
| STM4154 | *rpoC* | RNA polymerase, beta prime subunit | -6.25 | 2.15 |
| STM4334 | *efp* | elongation factor P (EF-P) | -3.83 | 1.51 |
| STM4393 | *rpsR* | 30S ribosomal subunit protein S18 | -4.32 | 0.99 |
| STM4394 | *rplI* | 50S ribosomal subunit protein L9 | -3.53 | 0.66 |
| STM4475 | *valS* | valine tRNA synthetase | -3.09 | 0.84 |

Cysteine Biosynthesis

| STM2430 | *cysK* | subunit of cysteine synthase A and O-acetylserine sulfhydrolase A | -17.16 | 6.10 |
| --- | --- | --- | --- | --- |
| STM2440 | *cysM* | cysteine synthase B (O-acetylserine sulfhydrolase B) | -3.03 | 1.26 |
| STM2441 | *cysA* | ABC superfamily (atp_bind), sulfate permease A protein; chromate resistance | -6.11 | 1.62 |
| STM2442 | *cysW* | ABC superfamily (membrane), thiosulfate permease W protein | -7.65 | 2.63 |
| STM2443 | *cysU* | ABC superfamily (membrane), thiosulfate transport protein | -6.05 | 1.65 |
| STM2444 | *cysP* | ABC superfamily (bind_prot), thiosulfate transport protein | -13.86 | 5.67 |
| STM2933 | *cysC* | adenosine 5'-phosphosulfate kinase | -10.52 | 4.07 |
| STM2934 | *cysN* | ATP-sulfurylase, subunit 1 (ATP:sulfate adenylyltransferase) | -11.49 | 3.84 |
| STM2935 | *cysD* | ATP-sulfurylase, subunit 1 (ATP:sulfate adenylyltransferase) | -47.12 | 21.85 |
| STM2946 | *cysH* | 3'-phosphoadenosine 5'-phosphosulfate (PAPS) reductase | -8.30 | 1.24 |
| STM2947 | *cysI* | sulfite reductase, alpha subunit, NADPH dependent | -8.79 | 3.46 |
| STM2948 | *cysJ* | sulfite reductase, beta (flavoprotein) subunit | -9.05 | 3.47 |

Oxidative Stress

| STM1165 | *grxB* | glutaredoxin 2 | -5.01 | 1.26 |
| --- | --- | --- | --- | --- |
| STM3702 | *grxC* | glutaredoxin 3 | -4.06 | 1.28 |
| STM4055 | *sodA* | superoxide dismutase, manganese | -6.10 | 1.20 |
| STM1318 | *katE* | catalase; hydroperoxidase HPII(III), RpoS dependent | -3.46 | 1.96 |
| STM3161 | *metC* | cystathionine beta-lyase (beta-cystathionase) | -3.13 | 0.53 |
| STM4101 | *metL* | aspartokinase II in bifunctional enxyme: aspartokinase II; homoserine dehydrogenase II | -4.97 | 1.45 |
| STM4188 | *metH* | B12-dependent homocysteine-N5-methyltetrahydrofolate transmethylase, repressor of metE and metF | -4.40 | 1.64 |

Respiratory Chain

| STM0439 | *cyoE* | protohaeme IX farnesyltransferase (haeme O biosynthesis) | -2.59 | 0.50 |
| --- | --- | --- | --- | --- |
| STM0440 | *cyoD* | cytochrome o ubiquinol oxidase subunit IV | -2.53 | 0.72 |
| STM0441 | *cyoC* | cytochrome o ubiquinol oxidase subunit III | -2.45 | 0.65 |
| STM0442 | *cyoB* | cytochrome o ubiquinol oxidase subunit I | -3.02 | 1.04 |
| STM0443 | *cyoA* | cytochrome o ubiquinol oxidase subunit II | -2.50 | 0.83 |
| STM0733 | *sdhD* | succinate dehydrogenase, hydrophobic subunit | -2.25 | 0.96 |
| STM0735 | *sdhB* | succinate dehydrogenase, Fe-S protein | -3.32 | 1.32 |
| STM2316 | *nuoN* | NADH dehydrogenase I chain N | -3.88 | 1.70 |
| STM2317 | *nuoM* | NADH dehydrogenase I chain M | -9.29 | 2.86 |
| STM2318 | *nuoL* | NADH dehydrogenase I chain L | -3.97 | 0.90 |
| STM2320 | *nuoJ* | NADH dehydrogenase I chain J | -5.80 | 9.30 |
| STM2322 | *nuoH* | NADH dehydrogenase I chain H | -4.44 | 1.69 |
| STM2323 | *nuoG* | NADH dehydrogenase I chain G | -4.75 | 0.92 |
| STM2324 | *nuoF* | NADH dehydrogenase I chain F | -5.48 | 1.40 |
| STM2325 | *nuoE* | NADH dehydrogenase I chain E | -5.06 | 1.47 |
| STM2326 | *nuoC* | NADH dehydrogenase I chain C,D | -11.32 | 4.81 |
| STM2327 | *nuoB* | NADH dehydrogenase I chain B | -4.52 | 1.39 |
| STM2328 | *nuoA* | NADH dehydrogenase I chain A | -8.33 | 3.43 |
| STM3867 | *atpA* | membrane-bound ATP synthase, F1 sector, alpha-subunit | -5.78 | 0.95 |
| STM3868 | *atpH* | membrane-bound ATP synthase, F1 sector, delta-subunit | -4.86 | 0.95 |
| STM3869 | *atpF* | membrane-bound ATP synthase, F0 sector, subunit b | -4.74 | 1.16 |
| STM3871 | *atpB* | membrane-bound ATP synthase, F0 sector, subunit a, important for FO assembly | -4.29 | 0.51 |
| STM3872 | *atpI* | membrane-bound ATP synthase subunit, F1-F0-type proton-ATPase | -2.61 | 0.49 |
| STM3865 | *atpD* | membrane-bound ATP synthase, F1 sector, beta-subunit | -5.53 | 0.59 |
| STM3866 | *atpG* | membrane-bound ATP synthase, F1 sector, gamma-subunit | -5.38 | 0.69 |
