# Supplementary material for: Nitric Oxide Antagonizes the Acid Tolerance Response that Protects Salmonella against Innate Gastric Defenses
Source: PLoS One. 2008 Mar 19;3(3):e1833. doi: 10.1371/journal.pone.0001833 (PMC2266805; doi:10.1371/journal.pone.0001833)
Supplement: Table S4 — (0.04 MB DOC) [file pone.0001833.s004.doc]

**Table S4**

**Primers**

| **Mutation constructed** | **Primer sequence** |
| --- | --- |
| *ΔphoP::FRT* | F:5'- CAGGATTCAGGTCACCAGGTCGATGCCGCAGAAGATGCCAGGGAAGCTGATTACTACCTTGCTGGAGCTGCTTCGAAGTT |
|  | R:5'-CGCATCCGGATACAGCTGAAGCATCAGCGAATCTTTGCTGACCACTTTACCGTTGTTACGTTCCGGGGATCCGTCGACCT |
| *ΔphoQ:FRT* | F:5'- GTGGCGCTGGTCGGCTATAGCGTAAGTTTTGATAAAACCACCTTTCGTTTGCTGCGCGGCGCTGGAGCTGCTTCGAAGTT |
|  | R:5'-CGCAGAAATCAGGTTATCTAACAACGGCGCGACGGGATGCAGTTCGCGGCTTAACAACACTTCCGGGGATCCGTCGACCT |
| *ΔlpxO::FRT* | F:5'-GCCGGAACGACGCGGGAGGATGTGATCAACAGATTTGAACTGCTCAGGACGCTCGCGTGCTGGAGCTGCTTCGAAGGTT |
|  | R:5'-TTTGCGCTCCAGCACTCTGTGTAACGGCCCCCACTGCAGTATCCACTCCCTGAACTCGCTTTCCGGGGATCCGTCGACCT |
| *Δfur::FRT* | F:5'-  gttggctcttcgaaagatttacacttatttagtcgcgtcatcgtgcgcgtgctcgtcttc |
|  | R:5'-  acatctgcgagagacttgcggttttcatttcggcatggcagtcctataatgatacgcatt |
| *ΔrpoS::km* | F:5'-  catgatttaaatgaagacgcggaatttgatgagaacggagctggagctgcttcgaagtt |
|  | R:5'-  gccttcaacctgaatctgacgaacacgttcacgcgtaagttccggggatccgtcgacct |
| *phoP::3xFLAG-FRT* | F:5'-  ACCGTACGCGGACAAGGATATCTTTTTGAATTGCGCGACTACAAAGACCATGACGG |
|  | R:5'-  CGACAGCGGCAGAAAATGGCGAGCAAATTTATTCATCATATGAATATCCTCCTTAG |
| *phoQ::3xFLAG-FRT* | F:5'-  CCCGTATGGAGGTCGTTTTTGGCCGACAGCATCCCACACAGAAAGAGGAAgactacaaagaccatgacgg |
|  | R:5'-  agtggcgttcaagaaagtcgggccagttaagagttaattgcatatgaatatcctccttag |
| *phoQ* complement | F^b^:5'-ACGGATGGATCCGTATCCGCAGGCTGGTATC |
|  | R^b^:5'-AGACTGCTCGAGAACCGATCCAGACCGTCAT |
| **RT-PCR** | **Primer sequence** |

| *rplN* | F:5'-GAGTTACCGGCCCAAAAATA |
| --- | --- |
|  | R:5'-CACCAAGAAGGGTGTTCGT |

|  | Hyb:5'-6-FAM^TM^-TAACAGCGAGCAGCCTATCGGTACG-BHQ^TM^-1* |
| --- | --- |
| *phoP* | F:5'-CTTGGATCCGGATTCATGCTGGCAGTTTT |
|  | R:5'-TGGAAGCTTTCCAGGTCATTTAAGAACAAAGAA |
| *phoQ* | F:5'-CTTGGATCCCGAGAGCGGCTTACAGAGAT |
|  | R:5'-TGGAAGCTTAGGGAGGGGGTAGTCTACGA |

| *mig-14* | F:5'-CCCTATACCGGGAGGTGTTT |
| --- | --- |
|  | R:5'-GGCAAGAAGCAGCGTAAATC |
| *phoN* | F:5'-ATCAGCAGCAACAATGCAAC |
|  | R:5'-TTCCAGGTTTCTGGCGTATC |
| *fliA* | F:5'-CTTGGATCCGGCGACAGATGATATGGTCA |
|  | R:5'-TGGAAGCTTCCCATTTTTACGCGATTCTC |
| *hmpA* | F:5'-CTTGGATCCTTAATGCTATCGCGGCCTAC |
|  | R:5'-TGGAAGCTTTCAAAGCTGGTGATCAGTGC |
| *rpoD* | F:5'-GTGGCTTGCAATTCCTTGAT |
|  | R:5'-AGCATCTGGCGAGAAATACG |

*****Dual-labeled oligonucleotide probes contain both the fluorescent dye 6-carboxyfluorescein (6-FAM^TM^) and Black Hole Quencher^TM^ 1 (BHQ^TM^-1).
